# Supplementary material for: Nutritional Counseling Based on Mindful Eating for the Eating Behavior of People Living with Overweight and Obesity: A Randomized Clinical Trial
Source: Nutrients. 2024 Dec 20;16(24):4388. doi: 10.3390/nu16244388 (PMC11676100; doi:10.3390/nu16244388)
Supplement: Supplementary file 1 [file nutrients-16-04388-s001.zip › nutrients-3344174-supplementary.pdf]

MINISTRY OF HEALTH OF BRAZIL

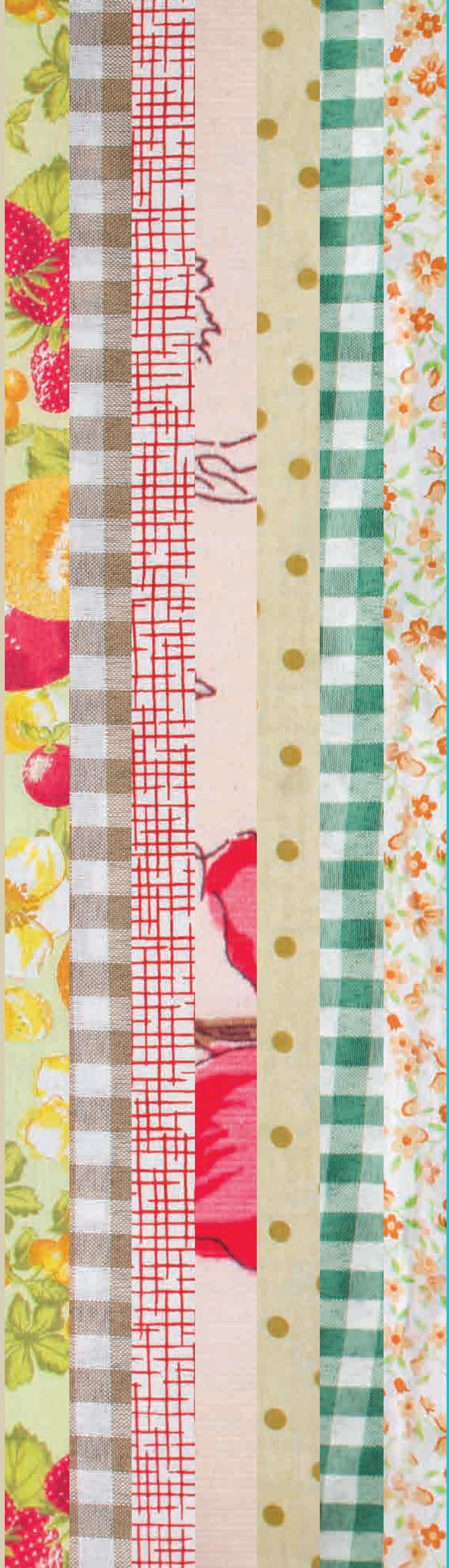

DIETARY GUIDELINES FOR THE BRAZILIAN POPULATION

Brasília — DF
2015

MINISTRY OF HEALTH OF BRAZIL
Secretariat of Health Care
Primary Health Care Department

DIETARY GUIDELINES FOR THE BRAZILIAN POPULATION

Brasília — DF
2015

2015 Ministry of Health of Brazil.

This work is provided under the terms of this Creative Commons Public License – Attribution – NonCommercial –ShareAlike 4.0 International Public License. It allowed the partial or total reproduction of this work provided that the source is mentioned

The Ministry of Health's institutional collection may be accessed, in its totality, at the Virtual library of the Ministry of Health of Brazil: <www.saude.gov.br/bvs>.

Print run: 1st edition – 2015 – Online version

Formulation, distribution and information:
MINISTRY OF HEALTH OF BRAZIL
Secretariat of Health Care
Primary Health Care Department
Edifício Premium, SAF Sul, Quadra 2,
lotes 5/6, bloco II, subsolo
CEP: 70070-600 – Brasília/DF
Fone: (61) 3315-9031
E-mail: dab@saude.gov.br
Site: dab.saude.gov.br

General editor:
Eduardo Alves Melo

Technical Coordination:
Patrícia Constante Jaime
General Coordination of Food and Nutrition – Ministry of Health

Technical Formulation:
Carlos Augusto Monteiro
Center for Epidemiological Research in Nutrition and Health, University of São Paulo

Technical Collaboration:
General Coordination of Food and Nutrition – Ministry of Health

Ana Carolina Feldenheimer da Silva
Ana Luisa Souza de Paiva
Bruna Pitasi Arguelhes
Fernanda Rauber
Gisele Ane Bortolini
Kelly Poliany de Souza Alves
Kimielle Cristina Silva
Lorena Toledo de Araújo Melo
Mara Lucia dos Santos Costa
Renata Guimarães Mendonça de Santana

Center for Epidemiological Research in Nutrition and Health, University of São Paulo (NUPENS/USP)

Ana Paula B. Martins
Carla Adriano Martins
Carlos Augusto Monteiro (Coordinator)
Daniela Silva Canella
Denise C. Coitinho
Enrique Jacoby
Francine Lima
Geoffrey Cannon
Jean-Claude Moubarac
Josefa Maria F. Garzillo
Larissa Galastri Baraldi
Maluh Barciotte
Maria Laura da Costa Louzada
Rafael Moreira Claro
Regina Rodrigues
Renata Bertazzi Levy
Semíramis Martins Álvares Domene

Pan-American Health Organization

Ana Carolina Feldenheimer da Silva
Janine Giuberti Coutinho

Editorial Coordination:
Laeticia Jensen Eble
Marco Aurélio Santana

Graphic Design and artwork:
Sávio Marques

Fotografias:
Department of Primary Care Collection
University of São Paulo Collection
Luciana Melo

Tradução:
Carlos Augusto Monteiro

Normalization:
Daniela Ferreira Barros da Silva – Editora MS/CGDI

Printed in Brazil

Cataloguing Data

Brazil. Ministry of Health of Brazil. Secretariat of Health Care. Primary Health Care Department.
Dietary Guidelines for the Brazilian population / Ministry of Health of Brazil, Secretariat of Health Care, Primary Health Care Department ; translated by Carlos Augusto Monteiro. – Brasília : Ministry of Health of Brazil, 2015.
150 p. : il.

Translation of: Guia Alimentar para a População Brasileira (2ª edição)
Access mode: World Wide Web: <http://189.28.128.100/dab/docs/portaldab/publicacoes/guia_alimentar_populacao_ingles.pdf>
ISBN 978-85-334-2242-1

1. Policy on Food and Nutrition. 2. Diet. 3. Dietary Guidelines.

CDU 612.3

Source Catalogin - General Coordination for Documents and Information - MS Publishing House - OS 2015/0116

Index titles:
Portuguese: Guia Alimentar para a População Brasileira
Spanish: Guía alimentaria para la población brasileña

CONTENTS

Preface 5
Preamble 7
Introduction 11
Chapter 1. Principles 15
Chapter 2. Choosing foods 25
Chapter 3. From foods to meals 53
Chapter 4. Modes of eating 91
Chapter 5. Understanding and overcoming obstacles 103
Ten steps to healthy diets 125
How to know more 131
Annex A – Process of elaboration of the newedition of the "Dietary Guidelines for the Brazilian Population" 143

PREFACE

During the last decades, Brazil has undergone several political, economic, social and cultural transformations that in turn resulted in changes in the ways of life of the population. The expansion of social policies in health, education, labour and employment and social assistance contributed to reducing social inequalities and allowed the country to grow in an inclusive manner. Rapid demographic, epidemiological and nutritional transition were also observed, and as a consequence other indicators were also influenced such as a higher life expectancy and a reduced number of offspring per woman, as well as important changes in health and food consumption patterns of the population.

The main diseases that currently affect the Brazilians are no longer acute but chronic. Despite the impressive decrease in malnutrition in children, micronutrient deficiencies and chronic malnutrition are still prevalent in vulnerable population groups, such as Indians, Maroons, and children and women living in vulnerable areas. At the same time, Brazil is experiencing a significant increase of overweight and obesity in all age groups and chronic diseases are the leading cause of death among adults. Now, one in two adults and one in three Brazilian children are overweight.

To tackle this scenario, there is an increasing need to expand intersectoral actions that resonate positively on the various determinants of health and nutrition. In this context, the health sector plays an important role in promoting adequate and healthy eating, which is already an expressed commitment in the National Food and Nutrition Policy and the National Health Promotion Policy. Promoting healthy eating in the Sistema Único de Saúde (SUS) – the Brazilian national health system – must be based on the dimensions of encouragement, support and protection of health, and must combine initiatives focused on healthy public policies, on creating healthy environments,

on developing personal skills and on reorienting health services from the perspective of health promotion.

The Dietary Guidelines for the Brazilian Population, published in 2006, presented the first official dietary guidelines for our population. Given the social changes experienced by Brazilian society, which impacted people´s health and nutrition conditions, the presentation of new recommendations became necessary. The second edition of the Dietary Guidelines for the Brazilian Population was submitted to a public consultation process that allowed its broad debate by various sectors of society and guided the construction of the final version which is presented here.

Assuming the rights to health and healthy food as general assumptions, the Guide is an official document that addresses the principles and recommendations of a healthy diet for the Brazilian population, representing a tool to support food and nutrition educational activities in SUS and also in other sectors. Considering the multiple determinants of feeding practices and the complexity and challenges that are involved in the shaping of current food systems, the Food Guide reinforces the commitment of the Ministry of Health to contribute to the development of strategies for the promotion and realization of the human right to adequate food.

Ministry of Health of Brazil

PREAMBLE

The World Health Organization (WHO) recommends, in its Global Strategy on Diet, Physical Activity and Health, that governments formulate and periodically revise national guidelines on food and nutrition. Revised and updated versions should take into account changes in population dietary patterns and states of health and disease, and also progress of scientific knowledge. These Guidelines are aimed at supporting food and nutrition education actions and national food and nutrition programmes and policies in Brazil.

Creation of dietary guidelines is part of a set of several national intersectoral actions that aim to improve the standards of diet and nutrition of the population and contribute toward promoting health. WHO therefore proposes that governments provide information and guidance to facilitate healthier food choices and habits, taking into account national and local food cultures, written for everybody and presented clearly and attractively.

As part of the Brazilian government's responsibility to promote the nation's health and food and nutrition security, the Ministry of Health published Dietary Guidelines for the Brazilian Population - Promoting Healthy Eating in 2006. These were the first official national dietary guidelines. They became a reference for people, families, communities, health professionals and government at all levels in promoting proper and healthy eating.

In line with WHO's recommendation for periodic revision, in 2011 the Ministry of Health began the process of developing a new edition of the Dietary Guidelines for the Brazilian Population. The decision was included as one of the goals of the Multi-Year Plan and of the First National Plan for Food and Nutrition Security, for the period 2012-2015.

The Dietary Guidelines for the Brazilian Population are part of the general strategy to promote adequate and healthy eating, which in turn is part of the Brazilian national policy for food and nutrition.

Adequate and healthy diet is a basic human right. This right implies ensuring permanent and regular access, in a socially fair manner, to food and ways of eating that satisfy the social and biological requirements of everybody. It also takes into account special dietary needs, and the needs to be culturally appropriate, and allow for differences in gender, race, and ethnicity. Adequate and healthy diet should be accessible both physically and financially, and harmonious in quantity and quality, meeting the needs of variety, balance, moderation, and pleasure. Furthermore, it should derive from sustainable practices of production and distribution.

The promotion of an adequate and healthy diet amounts to a set of strategies that aim to give everybody, personally and socially, ways in which to eat well in biological, social, cultural, economic and political aspects, while also paying attention to the sustainable use of natural resources and the protection of the environment. It is also a priority within the National Policy of Health Promotion, and as such, is meant to be implemented by the managers and professionals of the Brazilian National Health Service (SUS, the Unified Health System) in partnership with colleagues in other relevant sectors, emphasising the participation of all.

Improved and expanded accessibility and quality of the Brazilian national primary health care services network in recent years created the opportunity to encourage and support the inclusion of health promotion practices in the work of health teams throughout the country. Other policies and plans developed within the Unified Health System, such as the National Policy for Health Education of the General Public and the Strategic Action Plan for addressing chronic non-communicable diseases in Brazil, amplify this opportunity.

In an intersectoral context, the preparation of this new edition of the dietary guidelines occurs amid the strengthening of the institutionalisation of the National Food and Nutrition Security Policy, triggered by the publication of the Law for Food and Nutrition Security, and from the acknowledgement and inclusion of the right to food as a social right in the Federal Constitution.

The Law for Food and Nutrition Security establishes the System for Food and Nutrition Security through which the federal government, in partnership with organised civil society, formulates and implements policies, plans, programmes, and actions designed to assure the human right to adequate food This means, the right of every person to have uninterrupted physical and economic access to adequate food, or access to the means for obtaining food, without compromising other fundamental rights, such as those to health and education.

Two other documents for intersectoral public policies also relate to the need for effective health promotion and adequate and healthy diets. These are The Framework of Reference for Food and Nutrition Education and The Framework of Reference for General Public Education. These Dietary Guidelines are influenced by these two documents, so as to support work in food and nutritional education within the health sector and in other sectors.

Thus, these Dietary Guidelines for the Brazilian Population are designed to support and encourage healthy eating practices personally and collectively and also to support policies, programmes, and actions whose purpose is to encourage, protect, and promote the good health and the food and nutrition security of the whole Brazilian population

General Coordination of Food and Nutrition
Ministry of Health of Brazil, Brasília

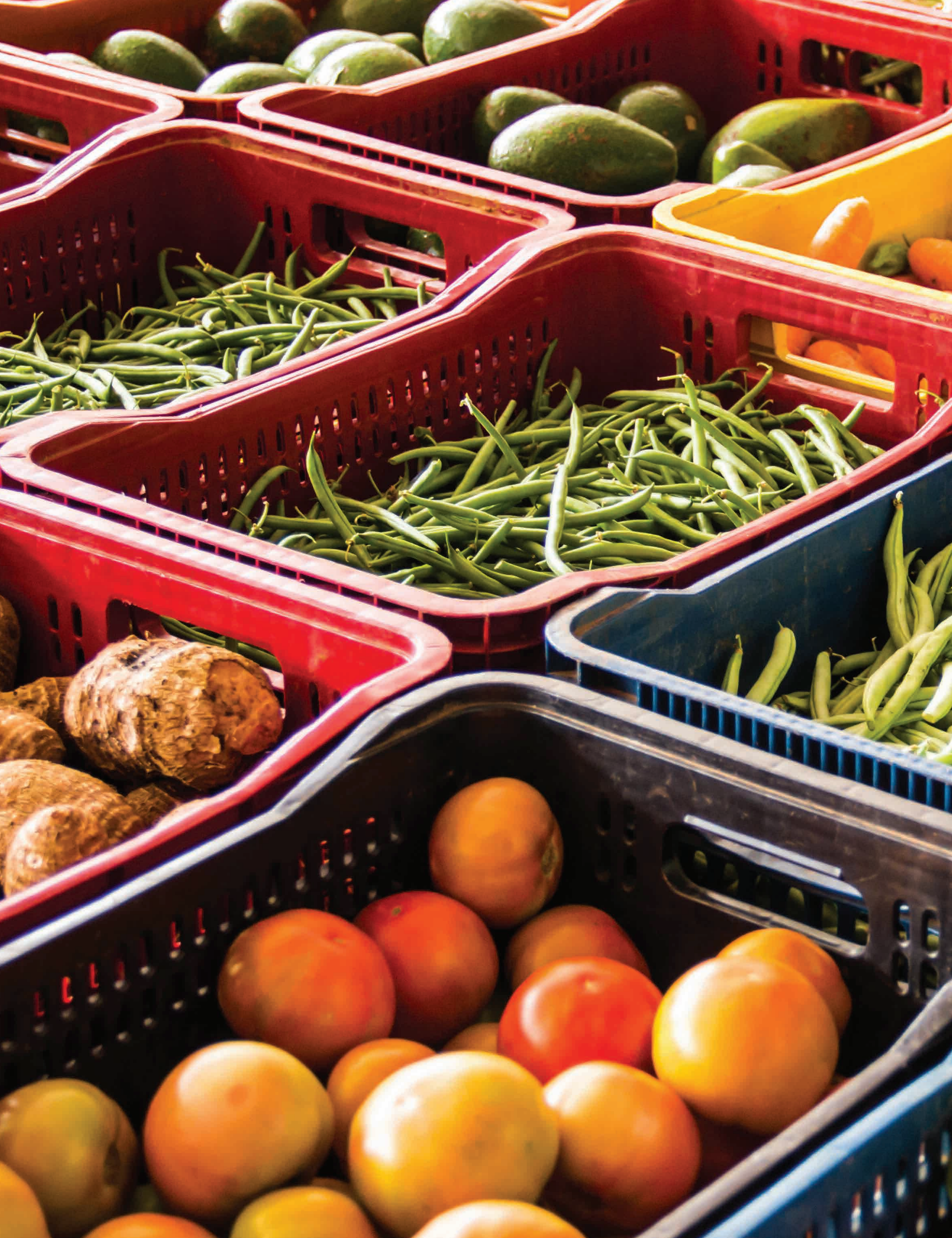

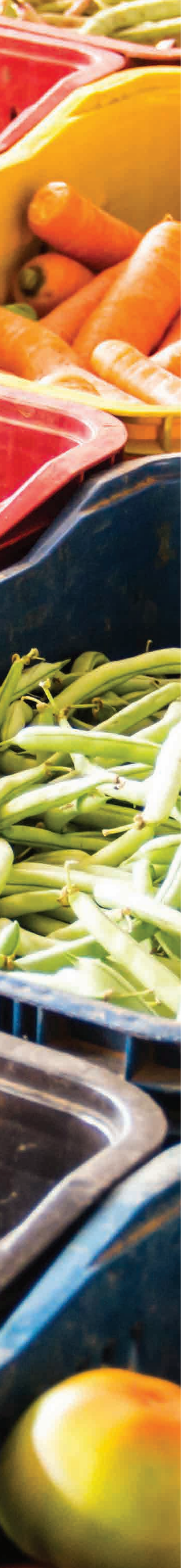

INTRODUCTION

The Dietary Guidelines for the Brazilian Population contain a full set of information and recommendations about eating. Their purpose is to promote the health and well-being of people, families, communities, and the whole Brazilian population, now and in future. They replace a previous version published in 2006.

These Guidelines are for all Brazilians. Some of these Brazilians will be workers whose jobs involves health promotion activities, such as health professionals, community workers, educators, capacity building trainers, as well as other professionals. These workers will be crucial with regard to the dissemination of the content of these Guidelines and to ensure that it is understood by all, including people who have difficulties in reading.

The hope is that these Guidelines will be used in people's homes, in health facilities, in schools, and in all other places concerned with health and its promotion, such as community centres, social assistance reference centres, and headquarters of social movements.

Although the focus of these Guidelines is to promote health and prevent diseases, their recommendations may be useful to those suffering from specific diseases. However, in this case, it is imperative that dieticians adapt the recommendations to the specific conditions of each person, supporting health professionals in the organisation of nutritional care.

Specific dietary guidance for children under the age of 2, consistent with the general recommendations of these Guidelines, is available in other publications of the Ministry of Health.

WHAT YOU WILL FIND IN THESE GUIDELINES

Chapter 1, 'Principles', specifies five shaping principles. The first states that diet, as well as involving nutrients, is about foods, meals, and eating modes, and also includes social and cultural aspects of dietary practices. The second states that sound dietary recommendations are tuned to changes in food supplies and population health patterns. The third acknowledges the interdependence between healthy diets and the social and environmental sustainability of the food system. The fourth states that reliable recommendations on diet come from a range of sources of evidence. The fifth states that dietary guidelines should enlarge people's choice of and right to adequate and healthy diets.

Chapter 2, 'Choosing foods', presents general recommendations about food choices. These recommendations, following the principles of these Guidelines, propose that natural or minimally processed foods, of different types and in large varieties, and predominantly of plant origin, are the basis of healthy diets.

Chapter 3, 'From foods to meals', provides guidance on how to combine foods in the form of meals. Its recommendations are derived from freshly prepared dishes and meals actually consumed by a substantial part of the Brazilian population.

Chapter 4, 'Modes of eating', addresses the circumstances - time, focus, place, and company - which influence how foods are metabolised by the body and also the pleasure afforded by eating.

Chapter 5 'Understanding and overcoming obstacles', identifies barriers in the way of healthy diets – information, supply, cost, culinary skills, time, advertising - and indicates how these can be surmounted, by people as consumers, family members, and as citizens.

Then follows Ten Steps to Healthy Diets, a self-contained summary of the recommendations of these Guidelines.

Finally, 'How to know more' recommends further reading, chapter by chapter.

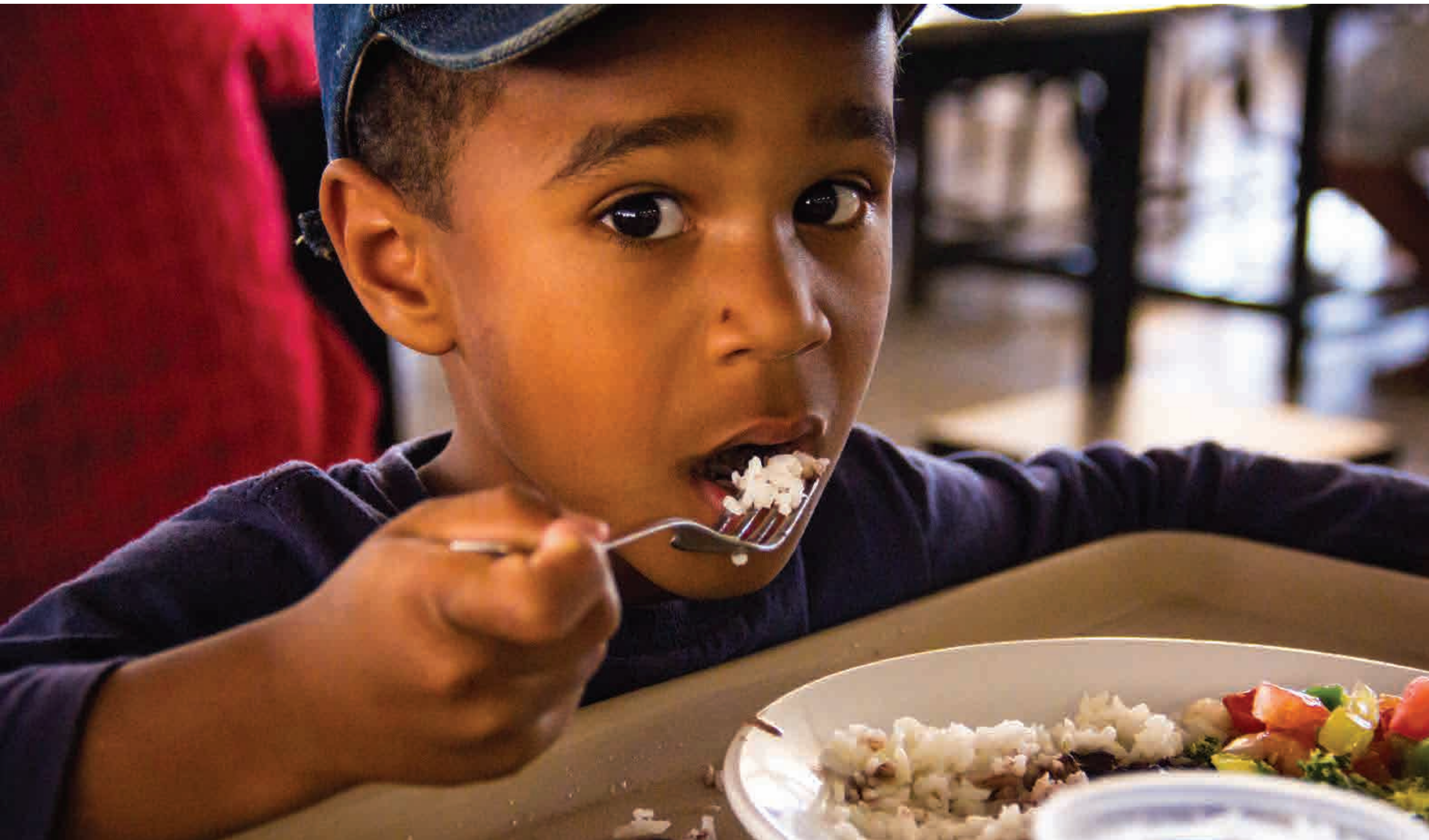

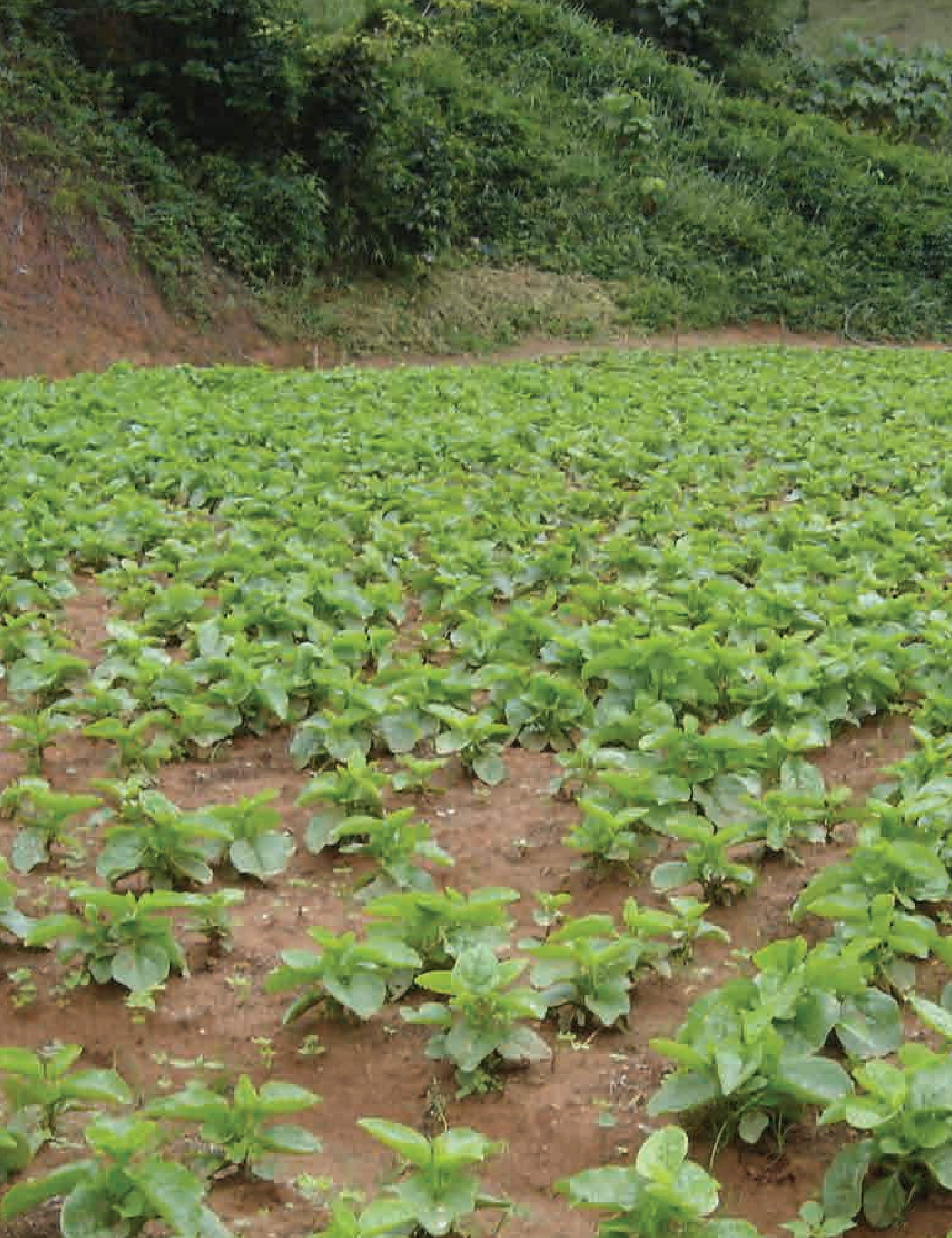

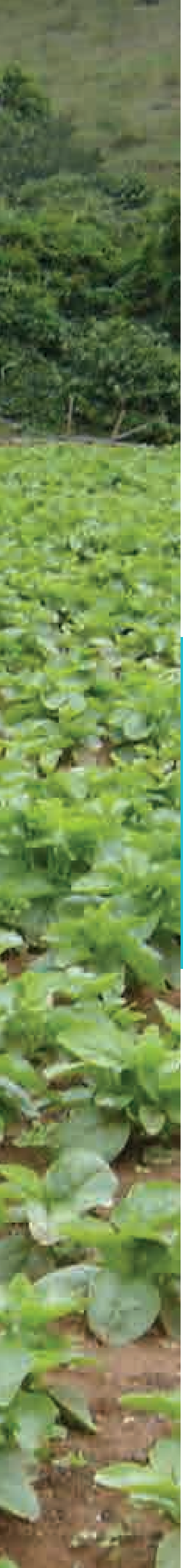

CHAPTER 1.
PRINCIPLES

All structured human activity is implicitly or explicitly guided by principles. Dietary guidelines are not an exception to this rule. The principles that shape these Guidelines are presented in this chapter.

DIET IS MORE THAN INTAKE OF NUTRIENTS

Diet refers to intake of nutrients, and also to the foods that contain and provide nutrients. Diet also refers to how foods are combined and prepared in the form of meals, how these meals are eaten, and also to cultural and social dimensions of food choices, food preparation and modes of eating, all of which affect health and well-being

Nutrients are vital and essential for good health. Equally important are foods that provide nutrients and other bioactive compounds, meals which result from the preparation and combination of foods, ways of eating and commensality, and the several cultural and social dimensions of diets.

Modern nutrition science emerged with the identification and isolation of nutrients present in foods and the study of the effects of these individual nutrients on the incidence of specific diseases. These studies were crucial for the formulation of policies and programmes designed to prevent specific nutritional deficiencies (such as of protein, and individual vitamins and minerals) and also cardiovascular

diseases inasmuch as these are caused by excessive consumption of sodium or some types of dietary fat.

However, the effect of individual nutrients was increasingly proving to be an inadequate explanation of the relationship between diet and health. Several studies show, for example, that protection against heart disease and certain types of cancer gained by consumption of substantial amounts of fruits or vegetables is not repeated with interventions based on medicines or supplements that contain such individual nutrients found in those foods. These studies indicate that the beneficial effect is from the food itself, and from the combinations of nutrients and other chemical compounds that are part of the food's matrix, more so than from individual nutrients.

Other studies show that the health benefits of traditional eating patterns, like the so-called "Mediterranean diet," is less due to individual foods and more due to the overall quality and balance of the foods that make up these patterns, and also to how they are prepared and eaten. Similarly, there is evidence that the contexts of the consumption of food—such as eating alone or on the sofa in front of the television, in contrast with sharing a meal, seated at a dining room table with family or friends— affect which foods are consumed, and in what quantities.

Specific foods, and dishes and meals made by combining and preparing these foods, in addition to ways of eating, are all part of the culture of a society. They shape the senses of personal identity, of self-determination, of belonging within a family and society, as well as the pleasure given by food, and thus to states of well-being.

For such reasons, these Guidelines take into consideration nutrients, foods, combinations of foods, dishes and meals, and the social and cultural dimensions of eating and dietary patterns.

DIETARY RECOMMENDATIONS NEED TO BE TUNED TO THEIR TIMES

Dietary recommendations should respond to changes in food supplies and in patterns of population health and well-being

Dietary patterns are now rapidly changing in most countries, particularly in economically emerging countries. The main changes involve displacing natural or minimally processed foods of plant origin (such as rice, beans, cassava, potatoes, root and leafy vegetables) and the preparation of meals based on these foods with industrialised food products that are ready for consumption. These changes, which have been intense in Brazil, have various ill-effects, including an imbalance in the supply of nutrients and an excessive intake of dietary energy.

In most countries, and again, particularly in economically emerging countries like Brazil, the rates of obesity and diabetes have been rapidly increasing. Other chronic diet-related diseases, such as hypertension (high blood pressure), heart diseases and some common cancers, have also been increasing. Previously viewed as problems which only affected older people, nowadays many of these diet-related diseases afflict young adults and even teenagers and children.

In contrast, undernutrition has been declining globally, although there are wide variations between countries and still high prevalence in most impoverished countries notably in Africa and parts of Asia. In Brazil, as a result of successful public policies focused on income distribution, eradication of absolute poverty, and expansion of access to basic health care, sanitation, and education to the population, the decline in undernutrition and infectious diseases associated with these conditions was remarkable over the last years. With the continuation of these public policies and the improvement of programmes for the control of specific micronutrient deficiencies among vulnerable groups of the population, undernutrition is expected to be under control within the near future.

The recommendations in these Guidelines are therefore designed to promote adequate and healthy diets in Brazil, and thus accelerate the decline of undernutrition, and check and reduce rates of obesity and diet-related chronic diseases.

HEALTHY DIETS DERIVE FROM SOCIALLY AND ENVIRONMENTALLY SUSTAINABLE FOOD SYSTEMS

Dietary recommendations need to take into account the impact of the means of production and distribution of food on social justice and environmental integrity

Depending on their characteristics, the production and the distribution of foods can be socially and environmentally sustainable, promoting justice and protection of the living and physical world, or else may generate social inequalities and threats to natural resources and biodiversity.

Factors affecting the social sustainability of food systems include the size and use of farms, the freedom of farmers to choose seeds, fertilisers and ways to control pests and diseases, working conditions and exposure to occupational hazards, the nature and number of

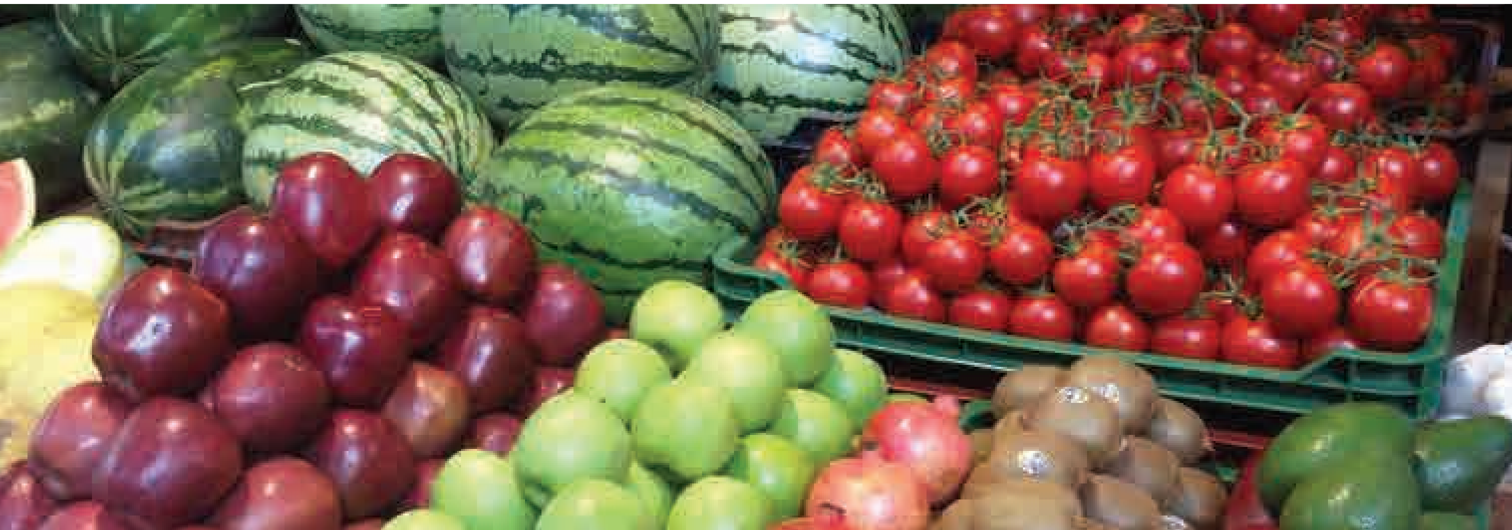

intermediaries between farmers and consumers, the fairness of the trading system, employment generation and the sharing of profit between capital and labour.

Factors affecting environmental sustainability include the techniques employed for soil conservation, use of organic or synthetic fertilisers, the planting of conventional or genetically modified seeds, chemical or biological control of pests and diseases, intensive or extensive forms of stockbreeding, the degree of use of antibiotics, production and treatment of wastes and residues, conservation of forests and biodiversity, intensity and nature of food processing, the distance between producers and consumers, transportation, and the amount of water and energy consumed.

In most parts of the world, the means of production and distribution of food has been changing, in ways that jeopardise the equitable distribution of wealth, the autonomy of farmers, the generation of employment and income opportunities, and the protection of natural resources and biodiversity, as well as production of safe and healthy food.

Long established sustainable food systems that favour family farming, traditional effective farming techniques and soil management, intensive use of labour, intercropping of various foods combined with the rearing of animals, minimal food processing done by farmers and by local industries, and supply systems based on small traders and municipal and local markets, are losing strength. They are being displaced by industrialised food systems. These include monocultures, very large farms that produce one or a few crops as raw materials for the manufacture of ultra-processed foods or for the feed used in the intensive production of animals. These industrial systems demand more and more land, intensive technology and mechanisation, heavy consumption of water and fuel, and use of chemical fertilisers, genetically modified seeds, pesticides, and antibiotics, and long-distance transportation. Such systems also have huge distribution networks that determine prices in ways that are unfair to producers and also to consumers.

In such ways, these Guidelines consider the means by which food is produced, distributed, and sold, favouring those which are socially and environmentally sustainable.

DIFFERENT SOURCES OF KNOWLEDGE INFORM SOUND DIETARY ADVICE

Diet has various dimensions and a complex relationship with population health and well-being. Therefore, the evidence required to construct recommendations on diet is generated from different sources of knowledge

Knowledge acquired from evidence produced by experimental and clinical studies is one vital base for dietary recommendations. This knowledge informs how different components of food – including nutrients and other chemical compounds with biological activity – interact with human physiology . Recent researches have demonstrated the presence of various chemical compounds with biological activity in foods, highlighting the presence of antioxidant and anti-inflammatory properties compounds in foods such as fruits, vegetables, nuts and fish. The interaction between nutrients and other compounds with biological activity is another area in which important scientific discoveries have been made.

Population studies on food and nutrition are critical for determining the practical relevance of the knowledge obtained from experimental and clinical studies, and also for generating hypotheses for investigation by such studies. Coupling population science with social studies such as from anthropological research gives insight into the evolution and adaptation of food supplies and dietary patterns. Such information is essential to ensure that recommendations are consistent, appropriate, and feasible, and relate well with the nature and culture of population dietary patterns.

Traditional dietary patterns, evolved and adapted often for very many generations, are also vital evidence. These amount to vast repositories of knowledge about the types and varieties of plants and animals best adapted to climate and terrain and other environmental factors, to techniques of production that have proved to be most productive and sustainable, and to the combination of foods and culinary preparations

that make meals beneficial and enjoyable. All this represents an essential natural experiment that needs to inform guidance on nutrition and on health in all senses.

So, the recommendations of these Guidelines are based on the evidence generated by a whole range of experimental, clinical, population, and social studies, and also on the knowledge implicit in the creation and development of traditional dietary patterns.

DIETARY GUIDELINES BROADEN AUTONOMY IN FOOD CHOICES

Access to reliable information on characteristics and determinants of healthy diets contributes towards people, families, and communities increasing their autonomy in making good food choices; it also contributes to leading them to demand compliance with the human right to adequate food.

Increasing autonomy in making choices of food involves the strengthening of people personally and as members of families and communities. This enables them to be agents able to protect their own health and that of people close to them, and also to act so as to affect social, environmental and other external determinants of health.

The constitution of autonomy for healthier eating choices depends on people personally, and also the environments where people live. In other words, it depends on people's ability to make choices, govern and produce their own lives, and also the external conditions, including the form of organisation of society and its laws, cultural values and access to educational and health services.

Adopting a healthy diet is not merely a matter of personal choice. Many factors—whether of a physical, economic, political, cultural, or social nature—can positively or negatively influence eating patterns. For

example, living in neighbourhoods or areas where there are markets that sell quality fruits and vegetables makes it more feasible to adopt healthy eating patterns. Other factors may hinder the adoption of these patterns, such as the higher cost of some minimally processed foods in comparison to the cost of ultra-processed products, the need to eat meals in places where healthy food options are not offered, and the intense exposure to advertising of unhealthy foods.

Thus, tools and strategies for food and nutrition education should support people, families, and communities in order to assist them in adopting eating habits that promote health. These should also give them the necessary skills to make decisions and transform reality, as well as to enforce the human right to adequate food. It is crucial that actions toward food and nutrition education be developed by the different public sectors, including those responsible for health, education, social development, agricultural development, and housing.

These Guidelines have been developed with the objective of facilitating access for people, families, and communities to knowledge on characteristics and determinants of healthy eating, enabling them to expand their autonomy to making better choices for themselves, reflect on everyday situations, seek changes in themselves and the environment they live in, contribute to ensuring food and nutrition security for all, and demand compliance with the human right to adequate food.

THE FIVE PRINCIPLES THAT SHAPE THESE GUIDELINES

Diet is more than intake of nutrients

Diet refers to intake of nutrients, and also to the foods that contain and provide nutrients. Diet also refers to how foods are combined and prepared in the form of meals, how these meals are eaten, and also to cultural and social dimensions of food choices, food preparation and modes of eating, all of which affect health and well-being

Dietary recommendations need to be tuned to their times

Dietary recommendations should respond to changes in food supplies and in patterns of population health and well-being.

Healthy diets derive from socially and environmentally sustainable food systems

Dietary recommendations need to take into account the impact of the means of production and distribution of food on social justice and environmental integrity.

Different sources of knowledge inform sound dietary advice

Diet has various dimensions and a complex relationship with population health and well-being. Therefore, the evidence required to construct recommendations on diet is generated from different sources of knowledge.

Dietary guidelines broaden autonomy in food choices

Access to reliable information on characteristics and determinants of healthy diets contribute toward people, families, and communities increasing their autonomy in making good food choices; it also contributes to leading them to demand the compliance to the human right to adequate food.

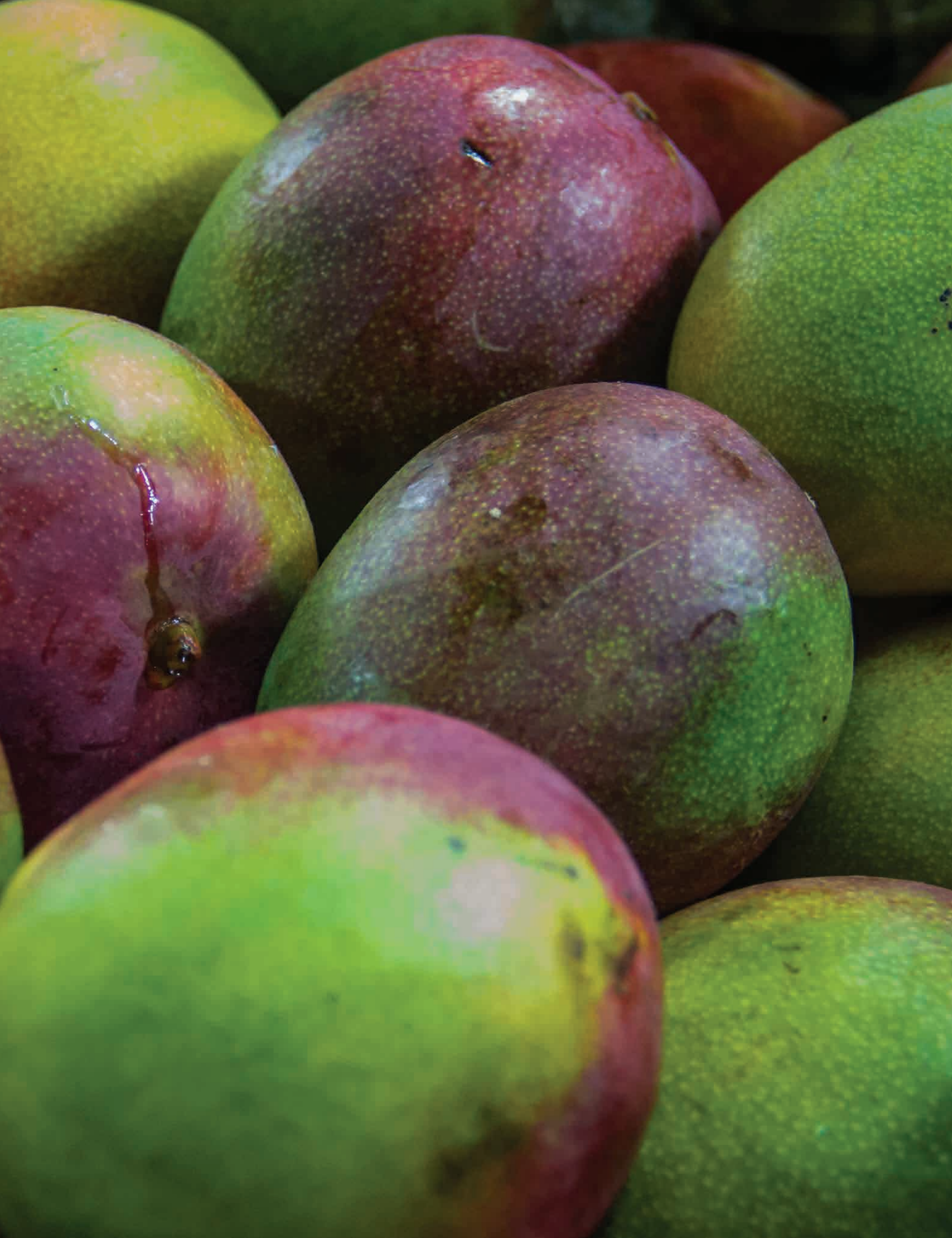

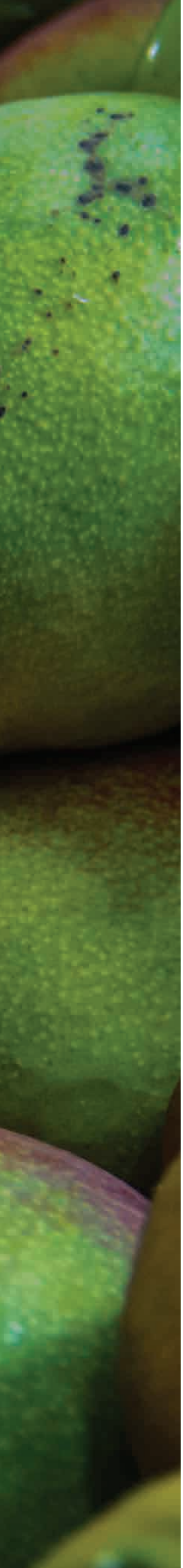

CHAPTER 2. CHOOSING FOODS

This chapter contains general recommendations to guide the choice of foods that make up nutritious, delicious and culturally appropriate diets, which also support and encourage socially and environmentally sustainable food systems. These recommendations follow the principles set out in the previous chapter, and, like all other recommendations of these Guidelines, aim at maximising the health and well-being of everybody, both now and in the future.

The recommendations of this chapter focus on the types of processing undergone by food prior to its purchasing, preparation, and consumption. As will be seen further ahead, the types of processing used in the production of a food largely determines its nutrient profile and its sensory qualities, in addition to influencing what other foods it will be consumed with, under what circumstances (when, where, and with whom), and even what quantities will be consumed. The social and environmental impact of the production of foods is also influenced by the type of processing used.

Four food categories, defined according to the type of processing used in food production, are addressed by the recommendations of this chapter.

The first category is of natural or minimally processed foods. Natural foods are those obtained directly from plants or animals (such as green leaves and fruits, or eggs and milk) and purchased for consumption without having undergone any alteration following their removal from nature. Minimally processed foods are natural foods which have been somewhat altered before being purchased. Examples include grains that are dried, polished, or ground as grits or

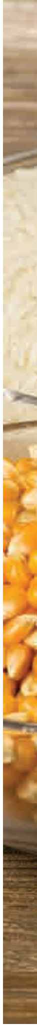

flour; roots, tubers and vegetables that are washed; cuts of meat that are cooled or frozen; and pasteurised milk.

The second category is of products that are extracted from natural foods or from nature itself, and used for seasoning and cooking food for the creation of culinary preparations. Examples include oils, fats, sugar, and salt.

The third category is of products that are manufactured essentially with the addition of salt or sugar to natural or minimally processed foods. Examples include canned and bottled vegetables or fruits, cheeses, and breads.

The fourth category is of products whose manufacture involves several stages and various processing techniques and ingredients, many of which are used exclusively by industry. Examples include packaged salty oily snacks, confectionery, soft drinks, sweetened breakfast cereals, packaged biscuits and cakes, chicken and fish nuggets or sticks, pre-prepared packaged pizzas, and instant noodles.

What follows, are recommendations for the consumption of the four food categories addressed by these Guidelines. Each category is defined and listed in some detail, together with reasons for the recommendations.

NATURAL OR MINIMALLY PROCESSED FOODS

Make natural or minimally processed foods the basis of your diet.

Natural or minimally processed foods, in great variety, mainly of plant origin, are the basis for diets that are nutritious, delicious, appropriate, and supportive of socially and environmentally sustainable food systems.

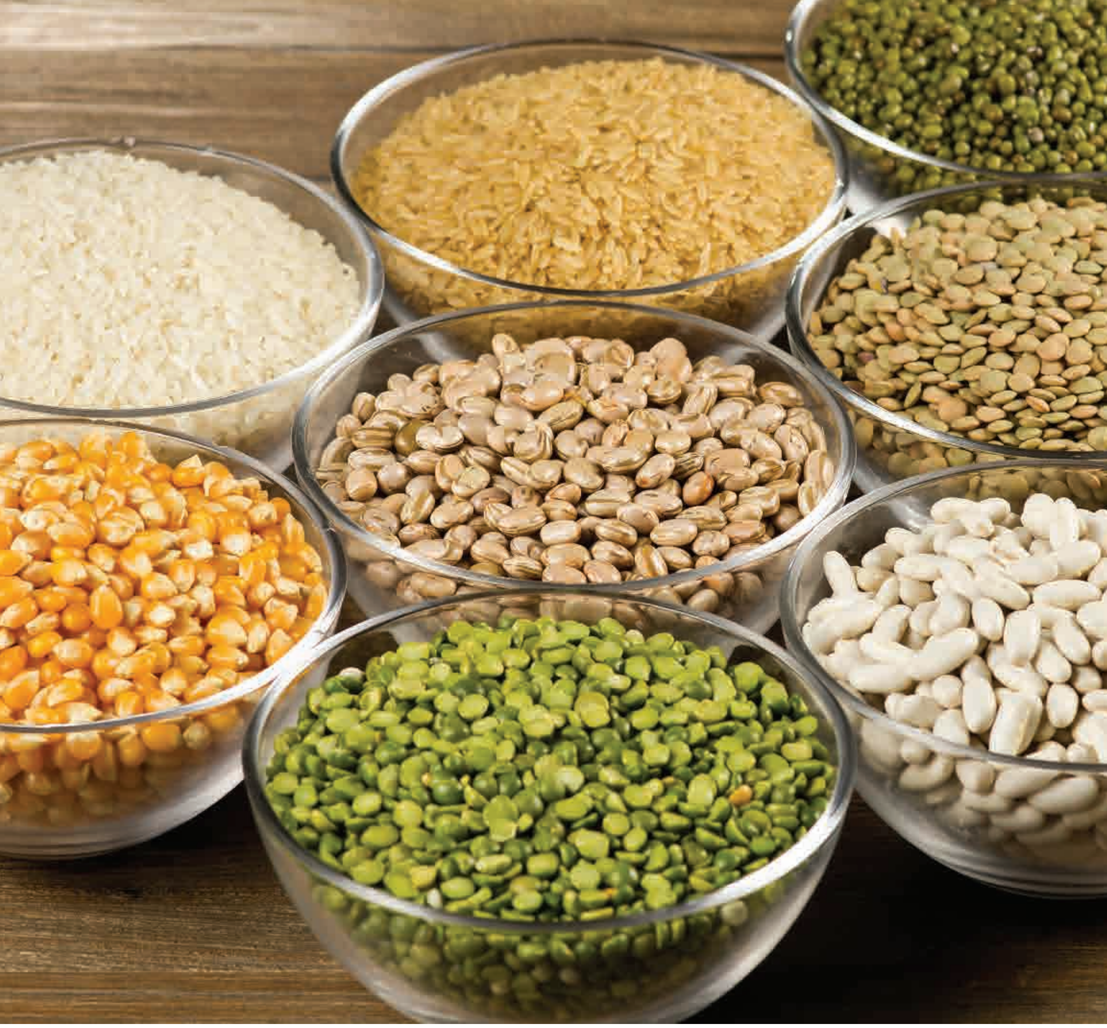
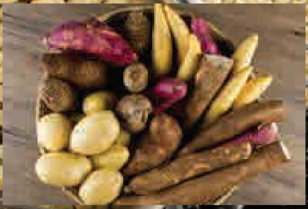
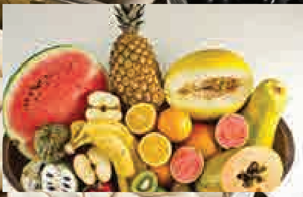
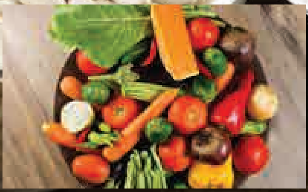
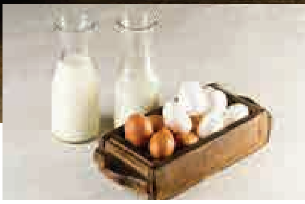
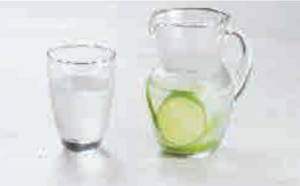
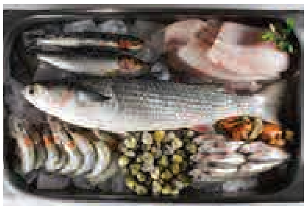
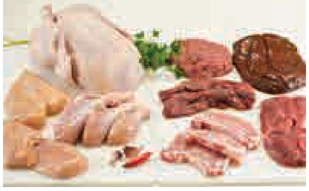

Natural or minimally processed foods include very many varieties of grains, roots and tubers, vegetables, fruits, fish, meat, milk, eggs, and also water.

As we have mentioned, natural foods are obtained directly from plants or animals and are acquired for consumption without having undergone any change after leaving nature.

The purchasing of natural foods is limited to a few varieties of foods such as fruits, vegetables, greens, roots, tubers, and eggs. Furthermore, it is common that even these foods undergo some changes prior to being acquired, like being washed, having inedible parts removed, or being refrigerated. Other foods, like rice, beans, and meat are commonly purchased after they have been dried, packaged, pasteurised, cooled, or

frozen. Grains like wheat and corn, and roots such as cassava, tend to be ground and consumed as pasta, tortillas and tapioca. Milk can be fermented and consumed in the form of yogurts and curds.

Cleaning, removal of inedible parts, drying, packaging, pasteurising, cooling, freezing, grinding, and fermentation are examples of minimal processes that transform natural foods into minimally processed foods. In all minimal processing, there is no addition of salt, sugar, oils, fats or other substances to the food.

Natural foods tend to deteriorate quickly and so need to be minimally processed before being acquired and consumed. Minimal processes preserve foods and make it possible to store them. Minimal processes can also decrease stages of food preparation (cleaning and removing inedible parts) or facilitate their digestion, or render them more palatable (grinding or fermentation).

In some cases, minimal processing techniques, such as the industrial refining of grains, reduce the nutrient content of foods. In such cases, a better choice is less processed foods like brown rice and whole grain flour. However, the benefits of minimal processing usually exceed possible disadvantages.

The table below defines the natural or minimally processed foods and lists examples. Following are the justifications that support the recommendation of these Guidelines to make these foods the basis of healthy diets.

NATURAL OR MINIMALLY PROCESSED FOODS

What are they?

Natural foods are obtained directly from plants or animals and do not undergo any alteration following their removal from nature. Minimally processed foods are natural foods that have been submitted to cleaning, removal of inedible or unwanted parts, fractioning, grinding, drying, fermentation, pasteurisation, cooling, freezing, or other processes that may subtract part of the food but which do not add oils, fats, sugar, salt or other substances to the original food.

Examples

Natural, packaged, cut, chilled or frozen vegetables, fruits, potatoes, cassava, and other roots and tubers; bulk or packaged white, parboiled and wholegrain rice; whole or separated corn; grains of wheat and other cereals; cassava, corn, or wheat grits and flours; dried or fresh pasta made from wheat flour and water; all types of beans; lentils, chickpeas, and other legumes; dried fruits, fruit juices fresh or pasteurised without added sugar or other substances; nuts, peanuts, and other oilseeds without salt or sugar; fresh and dried mushrooms and other fungi; fresh and dried herbs and spices; fresh, frozen, dried beef, pork, poultry and other meat and fish; pasteurised, 'long-life' and powdered milk; fresh and dried eggs, yoghurt without sugar; and tea, herbal infusions, coffee, and tap, spring and mineral water.

Reasons to base diets on many varieties of natural or minimally processed foods mainly of plant origin

BIOLOGICAL AND CULTURAL REASONS

The amount of energy per unit of weight (energy or calorie density) and the quantity of nutrients per unit of energy (nutrient density) of different types of natural or minimally processed foods vary very greatly.

Animal foods are usually good sources of proteins, vitamins and minerals, but contain no dietary fibre, and when fatty are energy-dense and contain excessive amounts of unhealthy saturated fats. Substantial consumption of such foods increases the risk of several chronic diseases.

Foods of plant origin are often good sources of dietary fibre and several nutrients, and almost all are low in energy density. But they need to be consumed in combination to supply an adequate range of nutrients.

In fact, apart from breast milk during the first six months of life, no food alone gives human beings the nutrient content that their body requires. This explains why the human species has evolved so as to be able to consume a wide variety of foods. It also explains why several societies and traditional food systems were established by combining foods of plant origin with nutrient profiles that complement each other, together with small amounts of food of animal origin.

Examples of combinations of plant foods are grains with legumes (common in Mexican cuisine, and in Brazil represented by rice and beans), cereals with vegetables (common in Asian cuisines and found in the Brazilian state of Pará in rice with jambu leaves), tubers with legumes (common in African countries and present in Brazilian tutu based on beans with cassava grits), and cereals or tubers with fruits (common in various cuisines and present in the rice with pequi fruit from Goiás and in cassava grits with the açaí berry from the Amazon).

In most traditional cuisines, meat, fish, eggs and other animal foods are consumed sparingly, as part of dishes and meals that are based on plant foods. Animal foods add flavour and also enhance the flavour of grains, beans, vegetables and tubers, and improve the nutritional composition of the overall diet. Use

of plant foods with intense flavours, such as garlic, onion, peppers, and herbs like basil and coriander, has a similar purpose.

Combination of a plentiful variety of foods of plant origin with small quantities of foods of animal origin results in nutritious, delicious and appropriate diets, which therefore are satisfying biologically, sensorially, and culturally.

SOCIAL AND ENVIRONMENTAL REASONS

Choosing diets based on a variety of foods of plant origin with sparing amounts of foods of animal origin implies the choice of a food system that is relatively equitable, and less stressful to the physical environment, for animals and biodiversity in general.

Food supplies and dietary patterns based on rice, beans, corn, cassava, potatoes, vegetables and fruits are socially beneficial. They encourage family farming and local economies, and living and producing in solidarity. They also promote biodiversity and reduce the environmental impact of food production and distribution.

Reduced consumption and thus production of animal foods will reduce emissions of the greenhouse gases responsible for global warming, of deforestation caused by creation of new grazing areas for cattle, and of intensive use of water. It will also reduce the number of intensive animal production systems, which are particularly harmful to the environment. The crowding of animals, characteristic of these systems, stresses the animals, increases animal wastes, requires systematic use of antimicrobial drugs, pollutes and contaminates groundwater, reservoirs, lakes and rivers, and generates diseases of animals that transmit to humans. Intensive production requires vast amounts of animal feed produced by monoculture systems producing soybeans and corn. Like all intensive agriculture, these also require intensive use of water, and of chemical pesticides and fertilisers that contaminate sources of water, degrade soil, increase pest resistance and reduce biodiversity. The use of genetically modified seeds in intensive agriculture is also a cause for environmental concern.

Foods of plant or animal origin derived from food systems that

promote a sustainable use of the natural resources are called organic and agro-ecological based foods. These food systems produce foods free of contaminants, protect biodiversity, contribute to a fairer distribution of productive lands and the creation of work, and respect and improve knowledge and traditional forms of production.

The more the people seek for organic and agro-ecological based foods, the greater will be the support received by agro-ecologic family farmers, and the more socially and environmentally sustainable food systems will become.

Although nutritionally balanced diets could be made up solely from natural or minimally processed foods, diets all over the world and throughout history have been and still are made from a combination of foods with culinary ingredients like oils, fats, salt and sugar. These products are used in home or restaurant kitchens for seasoning and cooking natural or minimally processed foods and to create with them delicious as well as nourishing dishes and meals. The second recommendation of this chapter applies to this category of products.

OILS, FATS, SALT AND SUGAR

Use oils, fats, salt, and sugar in small amounts for seasoning and cooking foods and to create culinary preparations.

As long as they are used in moderation in culinary preparations based on natural or minimally processed foods, oils, fats, salt, and sugar contribute toward diverse and delicious diets without rendering them nutritionally unbalanced.

Plant oils such as those made from soybeans, corn, sunflowers, or olives, fats such as butter and coconut fat, and sugar, are industrial products extracted from foods. Salt is extracted from nature.

All these products are used by people, in kitchens, to season and cook natural or minimally processed foods and to create with them varied and enjoyable dishes and meals. They are rarely consumed in the absence of such foods.

Thus, oils and fats are used in the cooking of rice and beans, the grilling and roasting of vegetables and meat, the frying of eggs, potatoes and cassava, and in preparation of soups and broths. Oils are also added to vegetable salads and green salads as seasoning. Salt is used as seasoning in all these preparations. It is also used in the preparation of homemade vegetable preserves and is added to the dough of flour and water used to make pies and homemade breads. Sugar is used to create homemade sweets made of fruits, milk, and eggs, and in the preparation of cakes and pies made of wheat, corn or cassava flour.

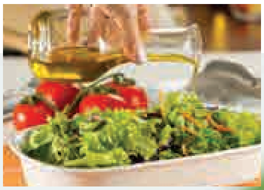

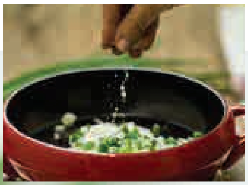

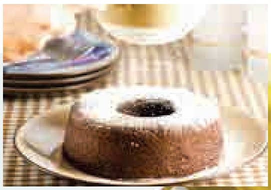

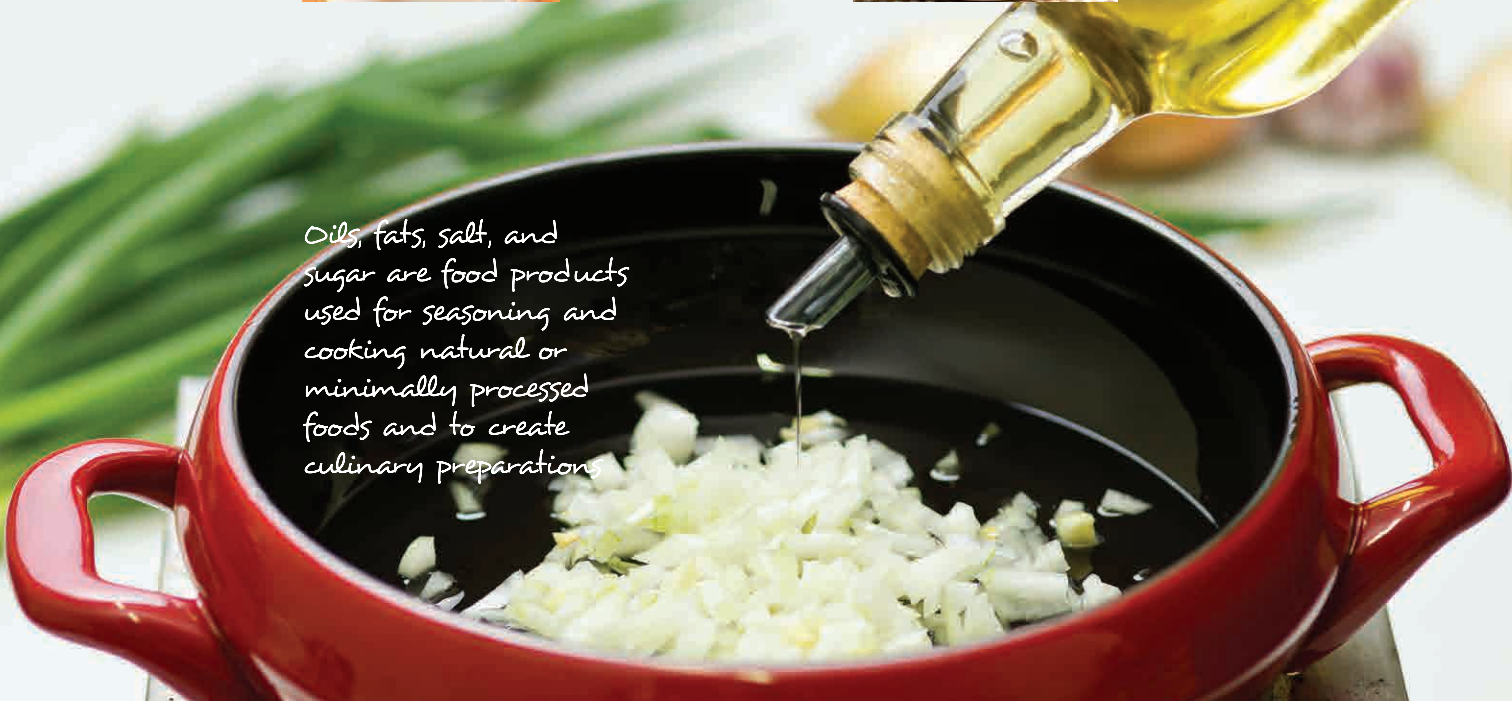

OILS, FATS, SALT, AND SUGAR

What are they?
They are products extracted from natural foods or from nature by processes such as pressing, grinding, crushing, pulverising, and refining. They are used in homes and restaurants to season and cook food and thus create varied and delicious dishes and meals of all types, including broths and soups, salads, pies, breads, cakes, sweets, and preserves.

Examples

Oils made from soybeans, corn, sunflower, or olives; butter, lard, coconut fat; white, brown and other types of sugar; refined or coarse salt.

REASONS WHY OILS, FATS, SALT, AND SUGAR SHOULD BE USED IN SMALL AMOUNTS

Oils, fats, and sugar are energy-dense. Oils and fats contain six times more calories per unit of energy than cooked grains, and twenty times more than cooked vegetables. Sugar is five or ten times more energy-dense than most fruits.

Oils, fats, sugar, and salt contain high amounts of dietary constituents that in small amounts are harmless but in large amounts are harmful. As well as being energy-dense, various oils and fats contain substantial amounts of saturated fats, which increase the risk of heart diseases. When consumed in large amounts, free sugar from any source including table sugar increases the risk of dental caries as well as obesity, and other chronic diseases. The sodium in salt is an essential nutrient but only in small amounts. In large amounts, it increases the risk of heart diseases.

However, as these products are essentially used to season and cook food, their impact on the nutritional quality of diets depends on the amount used in freshly prepared dishes and meals.

It is true that oils, fats, salt, and sugar tend to be quite easily accessible. They can be stored for a long time and are usually not expensive.

This encourages their excessive use. Yet, if used with restraint and appropriately combined with natural or minimally processed foods, they enable the creation of varied, delicious, and nutritionally balanced freshly prepared dishes and meals.

As will be seen in the next chapter of these Guidelines, natural or minimally processed foods and culinary preparations made from these foods plus oils, fats, salt, and sugar make diets having considerably superior nutritional quality than diets based on processed or ultra-processed foods, which are referred to in the next two recommendations.

PROCESSED FOODS

Limit the use of processed foods, consuming them in small amounts as ingredients in culinary preparations or as part of meals based on natural or minimally processed foods.

The ingredients and techniques used in the manufacture of processed foods—such as vegetables in brine, fruits in syrup, cheeses, and breads - unfavourably alter the nutritional composition of the foods from which they are derived.

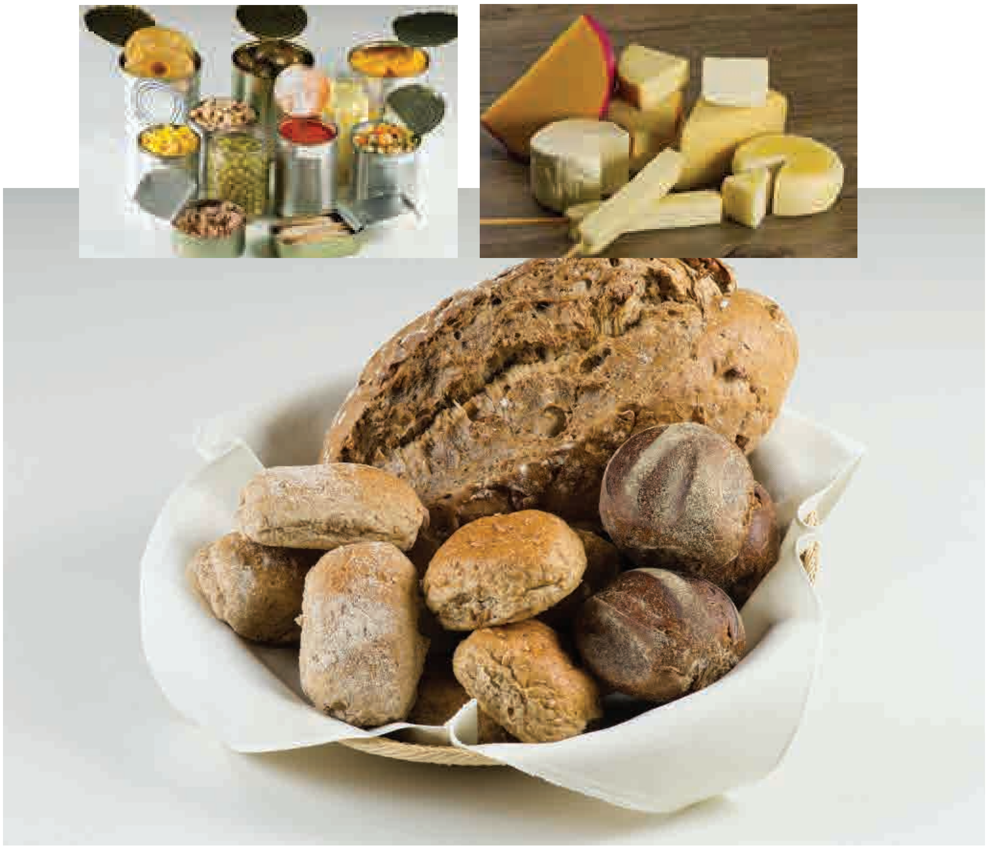

Processed foods include canned vegetables and fruits, cheeses, and breads made from wheat flour, yeast, water, and salt.

Processed foods are relatively simple and originally ancient products manufactured essentially with the addition of salt or sugar (or another substance of culinary use, such as oil or vinegar) to a natural or minimally processed food. The processing techniques used in the manufacture of these products are similar to culinary methods and include cooking, drying, fermentation, canning and bottling, and other preservation methods like salting, pickling, curing, and smoking. A processed food is usually easily recognisable as a modified version of the original food.

Processed foods include canned foods preserved in salt or vinegar or by pickling, fruits preserved in sugar, meat that is salted, smoked or cured, fish canned in salt or oil, cheeses made from milk, salt and fermenting agents, and breads made of wheat flour, yeast, water and salt.

In all such examples, the purpose of industrial processing is to increase the duration of natural or minimally processed foods and, frequently, to render more enjoyable.

Processed foods are often consumed as ingredients in dishes, as with cheese added to pasta and salted meats added to beans. At other times, as with breads and canned fish, processed foods are part of meals based on natural or minimally processed foods. However, processed foods may displace natural or minimally processed foods, such as when sandwiches are consumed instead of freshly prepared dishes. This use of processed foods is not recommended in these Guidelines.

PROCESSED FOODS

What are they?

Processed foods are products manufactured by industry with the use of salt, sugar, oil or other substances added to natural or minimally processed foods to preserve or to make them more palatable. They are derived directly from foods, and are recognised as versions of the original foods. They are usually consumed as a part of or as a side dish in culinary preparations made using natural or minimally processed foods.

Examples

Vegetables such as carrots, cucumbers, peas, heart-of-palm, onions, and cauliflower preserved in salt or vinegar, or by pickling; tomato extract or concentrates (with salt and/or sugar); fruits in sugar and candied fruits; beef jerky and bacon; canned sardine and tuna; other salted, smoked or cured meat or fish; cheeses; and breads made of wheat flour, yeast, water, and salt.

REASONS TO LIMIT CONSUMPTION OF PROCESSED FOODS

Processed foods retain the basic identity and most of the nutrients of the foods from which they are derived. But the added ingredients and the processing methods used in their manufacture compromise their nutritional composition.

The addition of salt and sugar, in quantities generally greater than used in culinary preparations, transforms the original food into a source of nutrients whose excessive consumption is associated with heart disease, obesity, and other chronic diseases.

The loss of water in the manufacturing of processed foods and the addition of sugar or oil transform foods with low or medium energy density, such as milk, fruits, fish, and wheat, into foods with high energy density, such as cheeses, sugared fruits, canned fish in oil, and breads. Diets that have high energy density increase the risk of obesity.

Therefore, the consumption of processed foods should be limited to small quantities, either as ingredients in culinary preparations, or as a side dish in meals based on natural or minimally processed foods. They should not displace freshly prepared meals. When processed

foods are chosen, it is important to consult the labels on the foods to opt for those with a lower content of salt or sugar.

ULTRA-PROCESSED FOODS

Avoid ultra-processed foods

Because of their ingredients, ultra-processed foods—such as packaged snacks, soft drinks, and instant noodles—are nutritionally unbalanced. As a result of their formulation and presentation, they tend to be consumed in excess, and displace natural or minimally processed foods. Their means of production, distribution, marketing, and consumption damage culture, social life, and the environment.

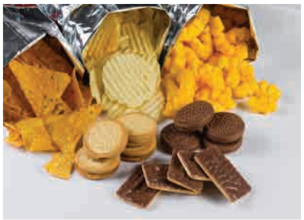
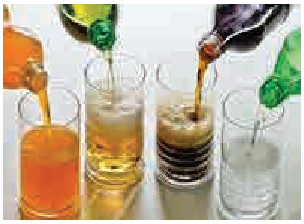
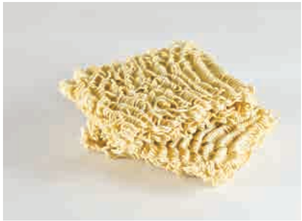

Ultra-processed foods include biscuits, packaged snacks, soft drinks, and instant noodles

The manufacturing of ultra-processed foods, generally done by large industries, involves several stages of processing techniques and many ingredients, including salt, sugar, oils and fats, and several substances for exclusive industrial use.

Ingredients for industrial use, common in ultra-processed foods, include soy and milk proteins, meat extracts, substances obtained from the additional processing of oils, fats, carbohydrates, and proteins, and substances synthesised in laboratories from food substrates or other organic sources, like petroleum and coal. Many of these synthesised substances serve as food additives whose function is to extend the duration of the ultra-processed foods or, more frequently, to give them hyper-attractive colour, flavour, aroma, and texture.

When natural or minimally processed foods are present, they represent a tiny proportion of all the ingredients found in ultra-processed foods.

Industrial processes employed in the manufacture of ultra-processed foods include those only used by industry, such as extrusion of corn flour to make packaged snacks, as well as industrial versions of culinary techniques, such as pre-processing with frying.

Ultra-processed foods include confectionery, drinks that are sweetened with sugar or artificial sweeteners, powders for juices, sausages and other products that are derived from meat and animal fat, pre-prepared frozen dishes, dried products such as cake mix, powdered soup, instant noodles, ready-seasonings, and an infinity of new products that arrive at the markets every year including packaged snacks, morning cereals, cereal bars, and ‘energy’ drinks. Breads and baked goods become ultra-processed foods when, in addition to wheat flour, yeast, water, and salt, their ingredients include substances such as hydrogenated vegetable fat, sugar, starch, whey, emulsifiers, and other additives.

A practical way of distinguishing ultra-processed foods from processed foods is to consult the list of ingredients, which by law, should provide labels on packaged foods that possess more than one ingredient. A high number of ingredients (usually five or more) and the presence of ingredients whose names are not familiar and are not used in culinary preparations (hydrogenated vegetable fat, interesterified oils, fructose syrup, protein isolates, bulking agents, thickeners, emulsifiers, colorants, flavour enhancers, and several other types of additives) identify ultra-processed foods.

Unlike processed foods, a majority of ultra-processed foods are consumed throughout the day, displacing foods like fruits, milk, and water or, as main meals, instead of culinary preparations. Therefore, these products tend to limit the consumption of natural or minimally processed foods.

ULTRA-PROCESSED FOODS

What are they?

Ultra-processed foods are industrial formulations made entirely or mostly from substances extracted from foods (oils, fats, sugar, starch, and proteins), derived from food constituents (hydrogenated fats and modified starch), or synthesised in laboratories from food substrates or other organic sources (flavour enhancers, colours, and several food additives used to make the product hyper-palatable). Manufacturing techniques include extrusion, moulding, and pre-processing by means of frying.

Examples

Fatty, sweet or salty packaged snacks, biscuits (cookies), ice-creams, candies and confectionery in general; cola, soda, and other soft drinks; sweetened juices and 'energy' drinks; sweetened breakfast cereals; cakes and cake mix, and cereal bars; sweetened and flavoured yogurts and dairy drinks; canned, packaged, dehydrated and other 'instant' soups, noodles, and seasonings; pre-prepared meat, fish, vegetables, pizza and pasta dishes, burgers, hot dogs, sausages, poultry and fish 'nuggets' and 'sticks' and other animal products made from remnants; sliced bread, hamburger or hot dog breads, sweet breads, and baked products in general made with ingredients such as hydrogenated vegetable fat, sugar, yeast, whey, emulsifiers, and other additives.

REASONS TO AVOID CONSUMPTION OF ULTRA-PROCESSED FOODS

There are many reasons to avoiding the consumption of ultra-processed foods. These reasons are related to the nutritional composition of these products, the characteristics that link them to the excessive consumption of dietary energy, and the impact that the ways in which they are produced, distributed, marketed and consumed creates on culture, social life and the environment.

Ultra-processed foods have an unbalanced nutritional composition.

The main ingredients of ultra-processed foods make them fatty or sugary, or both. It is common that these products have high content of sodium because of the addition of a lot salt. The addition of salt is necessary to extend the duration of the products and intensify their flavour or even to disguise undesired flavours that result from additives or from substances generated from the techniques involved in the ultra-processing.

For ultra-processed foods to last longer and not become prematurely rancid, they are often prepared with fats that resist oxidation. These fats, however, tend to obstruct arteries that carry blood within our bodies. Particularly common in ultra-processed foods are vegetable oils with a high content of saturated fats and hydrogenated fats. Apart from being high in saturated fats, hydrogenated fats also contain trans fats.

Ultra-processed foods tend to have a very low content of dietary fibre, which protects against heart diseases, diabetes, and some common cancers. The absence of fibre is a result of the lack of or the limited presence of natural or minimally processed foods in these products. This same reason also results in ultra-processed foods having low contents of vitamins, minerals, and other substances with biological activity, which are naturally present in natural or minimally processed foods.

Ultra-processed foods are now often reformulated and advertised as if they are healthy, being labelled as for example ‘light’ or ‘diet’, or low in fat or sugar, or free from trans fats, or high in fibre or vitamins and minerals. These adjustments may improve the products which however remain ultra-processed and unhealthy, and certainly not good alternatives to natural or minimally processed foods and freshly made dishes and meals.

Therefore, in summary, the unbalanced nutritional composition of the ingredients of ultra-processed foods favours heart disease, diabetes, and common cancers, and contributes towards increasing the risk of nutritional deficiencies. Furthermore, although each additive used in these products have to pass tests and be approved by health authorities, the long-term and cumulative effects on health of being exposed to various additives are not always known.

Ultra-processed foods promote excessive consumption of dietary energy

Ultra-processed foods disturb mechanisms located in the digestive system and the brain that ensure that the intake and expenditure of dietary energy is balanced. These mechanisms tend to underestimate the energy contained in ultra-processed foods, with the result that the sense of satiety occurs only after excess consumption. Excess dietary energy is stored as body fat. The result is obesity.

The high quantity of energy per gram is one of the main features of ultra-processed foods which can disturb the regulation of energy balance. The energy density of ultra-processed foods ranges from about 2.5 calories per gram (most baked products) to about 4 calories per gram (cereal bars), and may reach up to 5 calories per gram in the case of packaged snack products and filled biscuits. Such energy density is 2 to 5 times higher than that of common freshly prepared dishes, such as the traditional Brazilian mix of two servings of rice to one serving of beans.

Other attributes common to many ultra-processed foods can also compromise the mechanisms that signal satiety and control the appetite, thus further favouring the involuntary consumption of calories and increasing the risk of obesity.

- **Hyper-palatable.** Ultra-processed foods are formulated with combinations of oils, fats, sugars, salt and additives, so as to

be hyper-palatable, and sometimes habit-forming or even in effect addictive. The advertising of some of these products correctly identifies them as 'irresistible'.

- **Designed to be consumed mindlessly.** Most ultra-processed foods are made ready-to-consume, without plates, cutlery or tables. They are eaten at home while watching television, at a desk at work, or while walking in a street. These characteristics are often advertised as desirable.
- **Gigantic sizes.** The cost to the manufacturer of the ingredients of ultra-processed foods is usually low and often trivial. So they are commonly sold in 'super-sizes' with discounted prices. This induces over-consumption.
- **'Liquid calories'.** Sweetened drinks 'deceive' the body's mechanisms that signal satiety. All such drinks – colas, other soft drinks, 'energy' drinks, fruit juices with added sugars, other fruit drinks – are therefore very likely to be over-consumed.

Ultra-processed foods damage culture, social life, and the environment

The reasons described until here, seen in their totality, would alone be sufficient to justify the recommendation for avoiding the consumption of ultra-processed foods. Their ingredients and the processing techniques used in their manufacture make them intrinsically unhealthy.

However, there are still other reasons to avoid ultra-processed foods. These arise from the impact of their production, distribution, marketing, and consumption on culture, social life, and the environment. These reasons also, indirectly, affect human health and well-being.

Impact on culture. Brands, packages, labels, and the contents of ultra-processed foods tend to be identical throughout the world. A type of soft drink made by one giant manufacturer is essentially the same the world over. Types of burger made by various manufacturers are much the same everywhere. Leading brands are promoted often using the same

entertainers, models, music and slogans everywhere, including on television, the internet and social media. They are disseminated by means of intensive and aggressive advertising campaigns, including the launching of hundreds of new products every year, which leads to a false sense of diversity. Because of these campaigns, genuine food cultures come to be regarded as uninteresting. All this creates a sense especially to children and young people that the culture and identity of their own country, region, ethnicity and tradition including food culture and patterns, are boring. Young people especially are being induced by major manufacturers, in effect acting in concert, to have a false sense of belonging in a superior, modern, high cost and expense consumer culture.

Impact on social life. Ultra-processed foods are formulated and packaged to be ready-to-consume without any preparation. This makes meals and sharing of food at table unnecessary. Ultra-processed foods can be consumed anytime, anywhere, often when being entertained or when working, walking in a street, driving, or talking on a phone. These are mostly isolated situations, which are disguised by advertisements suggesting that such products promote social interaction, which they do not.

Impact on environment. The need for cheap oils, sugar and other raw materials for ultra-processed foods creates monocultures and farms producing for export and not for local consumption. Intensive farming of raw materials is dependent on pesticides and intensive use of fertilisers and water. The manufacture and distribution of most ultra-processed foods involves long transport routes, and thus excess use of non-renewable energy and water, and emission of pollutants. This all results in environmental degradation and pollution, loss of biodiversity, and draining and loss of water, energy and other natural resources. Production and consumption also causes creation of vast amounts of waste and garbage, dumped in disgusting and dangerous landfill sites. Overall, ultra-processed foods are a serious threat to the sustained survival of the planet.

For all the reasons described above, ultra-processed foods should be avoided.

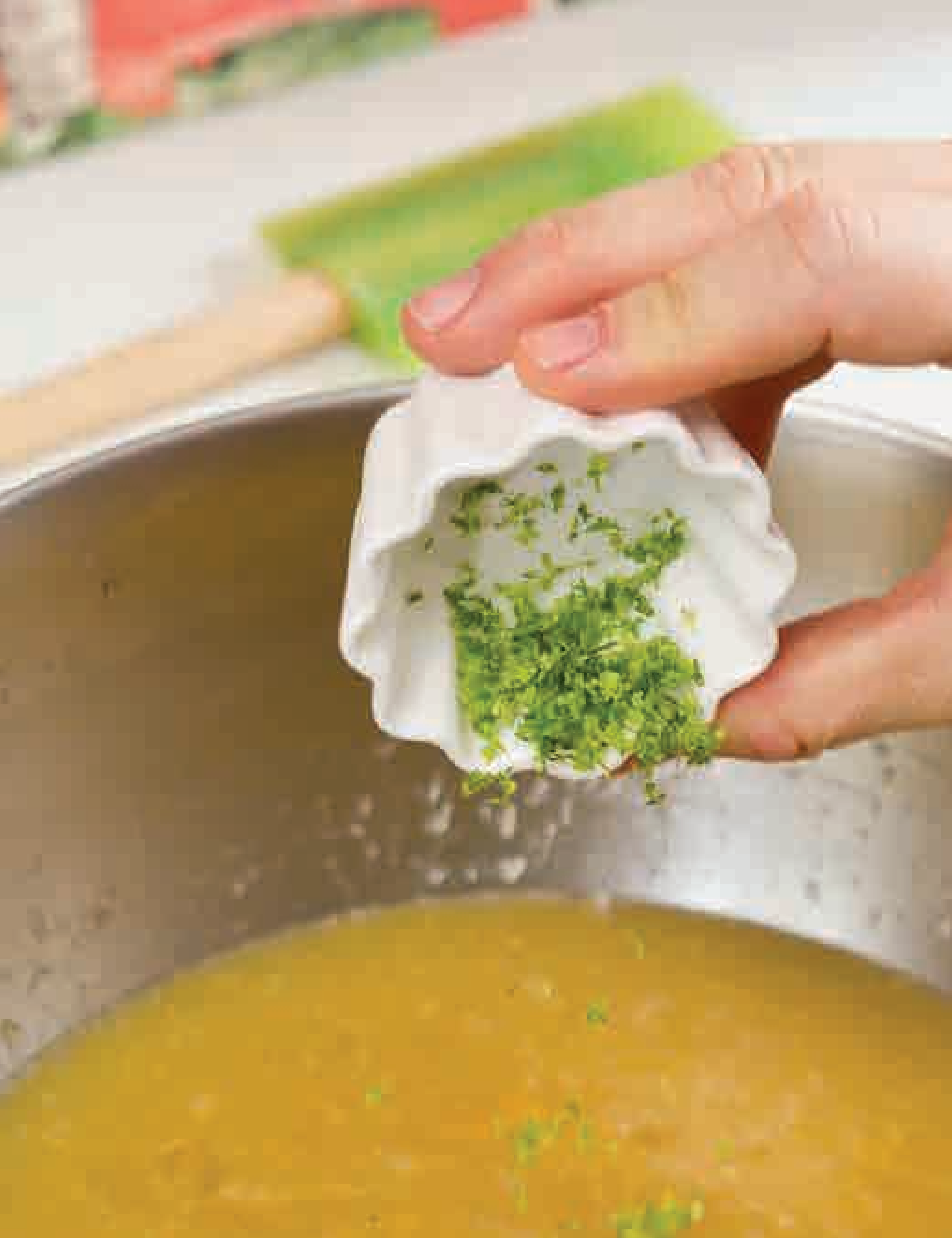

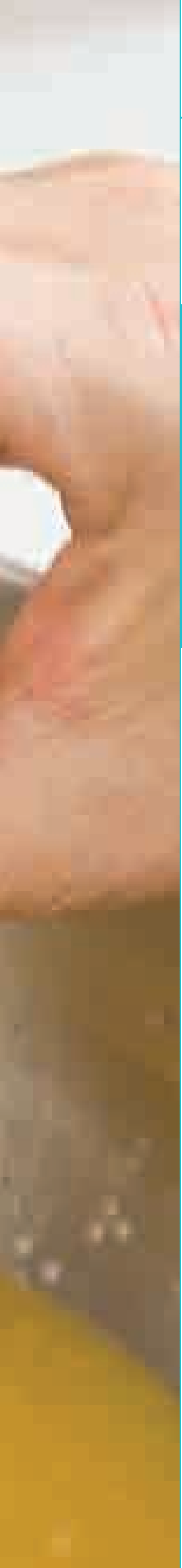

THE GOLDEN RULE

ALWAYS PREFER NATURAL OR MINIMALLY PROCESSED FOODS AND FRESHLY MADE DISHES AND MEALS TO ULTRA-PROCESSED FOODS

Like all golden rules, the overall rule here is easy to remember and follow: always prefer natural or minimally processed foods and freshly made dishes and meals to ultra-processed foods.

In other words, opt for water, milk, and fruits instead of soft drinks, dairy drinks, and biscuits, do not replace freshly prepared dishes (broth, soups, salads, sauces, rice and beans, pasta, steamed vegetables, pies) with products that do not require culinary preparation (packaged soups, instant noodles, pre-prepared frozen dishes, sandwiches, cold cuts and sausages, industrialised sauces, ready-mixes for cakes), and stick to homemade desserts, avoiding industrialised ones.

FINALLY

The four recommendations and the golden rule—highlighted yet again at the end of this chapter—are universal, and therefore can be applied everywhere. Specific orientations for the Brazilian population, developing and detailing the recommendations, are in the following chapter.

FOUR RECOMMENDATIONS AND ONE GOLDEN RULE

Make natural or minimally processed foods the basis of your diet

Natural or minimally processed foods, in great variety, mainly of plant origin, are the basis for diets that are nutritious, delicious, appropriate, and supportive of socially and environmentally sustainable food systems

Use oils, fats, salt, and sugar in small amounts for seasoning and cooking foods and to create culinary preparations

As long as they are used in moderation in culinary preparations based on natural or minimally processed foods, oils, fats, salt, and sugar contribute toward diverse and delicious diets without rendering them nutritionally unbalanced.

Limit the use of processed foods, consuming them in small amounts as ingredients in culinary preparations or as part of meals based on natural or minimally processed foods

The ingredients and techniques used in the manufacture of processed foods—such as vegetables in brine, fruits in syrup, cheeses and breads - unfavourably alter the nutritional composition of the foods from which they are derived.

Avoid ultra-processed foods

Because of their ingredients, ultra-processed foods —such as packaged snacks, soft drinks, and instant noodles—are nutritionally unbalanced. As a result of their formulation and presentation, they tend to be consumed in excess, and displace natural or minimally processed foods. Their means of production, distribution, marketing, and consumption damage culture, social life, and the environment.

The golden rule. Always prefer natural or minimally processed foods and freshly made dishes and meals to ultra-processed foods

Opt for water, milk, and fruits instead of soft drinks, dairy drinks, and biscuits Do not replace freshly prepared dishes (broth, soups, salads, sauces, rice and beans, pasta, steamed vegetables, pies) with products that do not require culinary preparation (packaged snacks and soups, instant noodles, pre-prepared frozen dishes, sandwiches, cold cuts and sausages, industrialised sauces, ready-mixes for cakes), and stick to homemade desserts, avoiding industrialised ones.

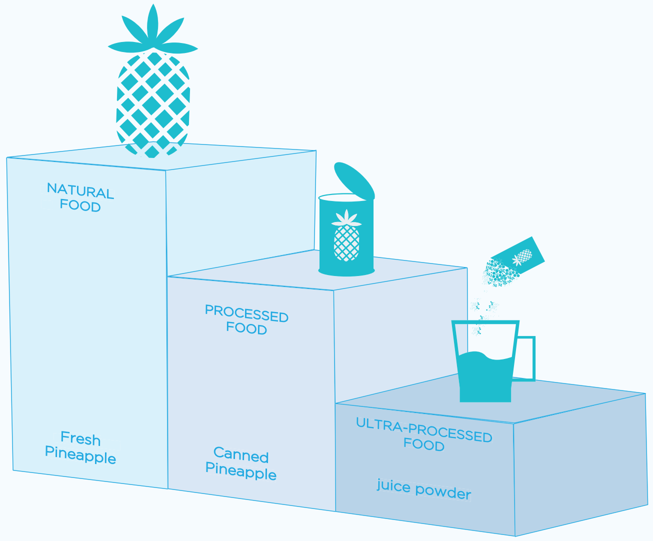
NATURAL FOOD
Fresh Pineapple
PROCESSED FOOD
Canned Pineapple
ULTRA-PROCESSED FOOD
juice powder

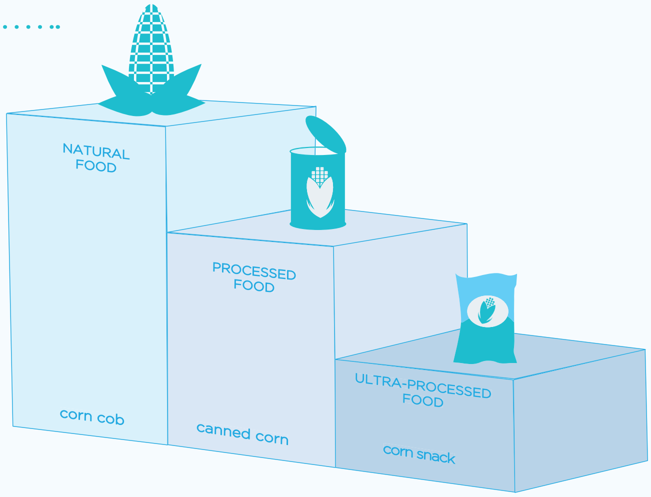
NATURAL FOOD
corn cob
PROCESSED FOOD
canned corn
ULTRA-PROCESSED FOOD
corn snack

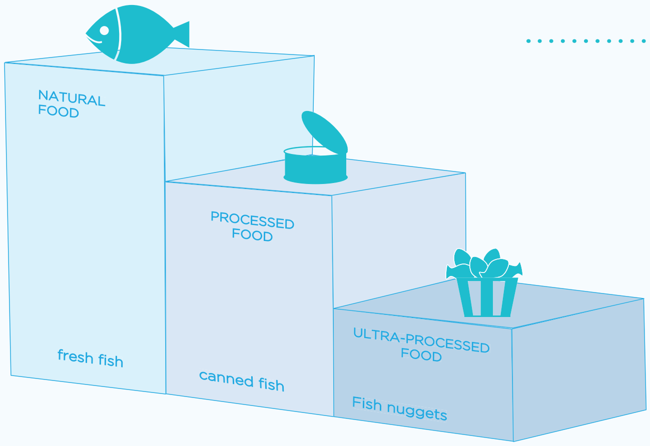
NATURAL FOOD
fresh fish
PROCESSED FOOD
canned fish
ULTRA-PROCESSED FOOD
Fish nuggets

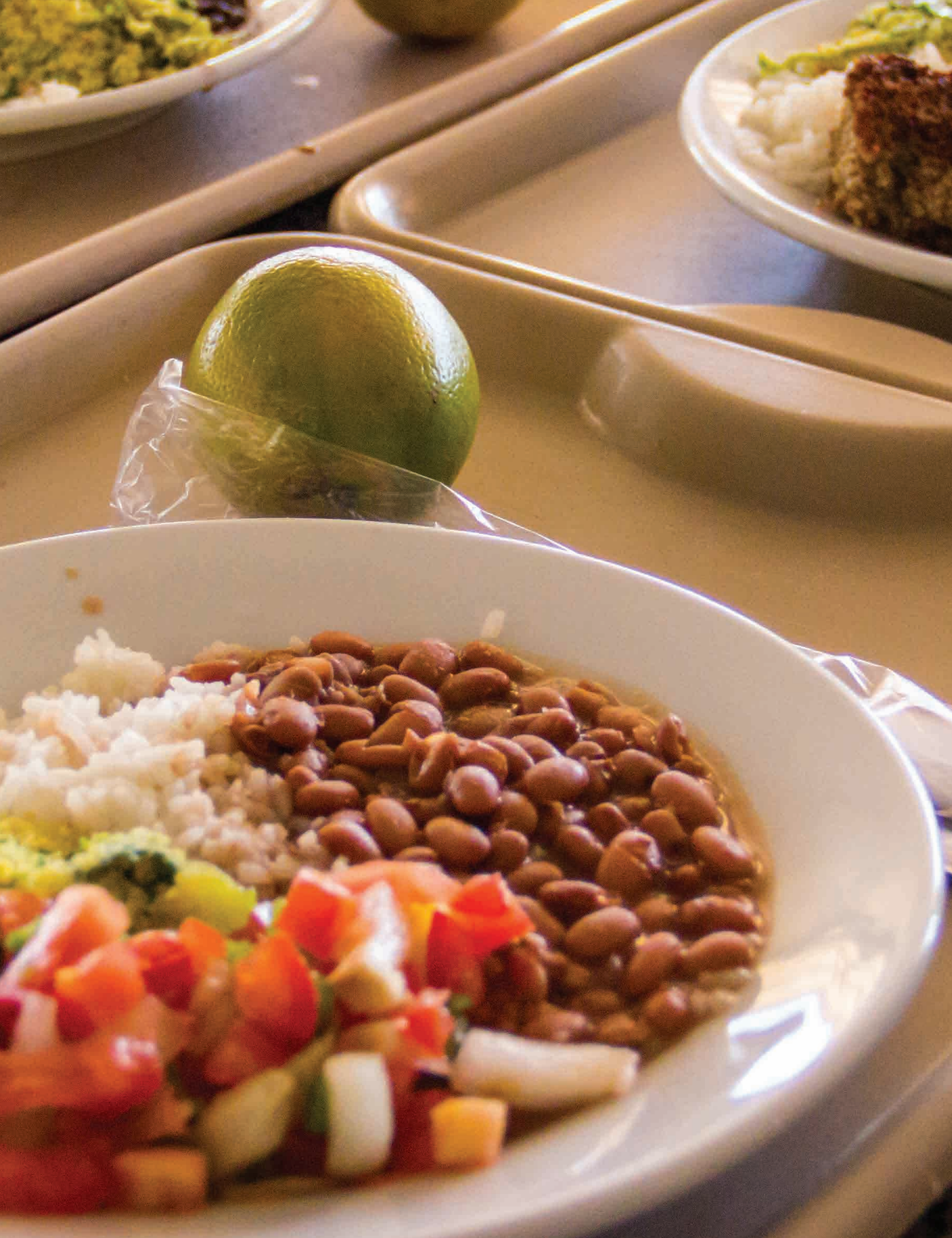

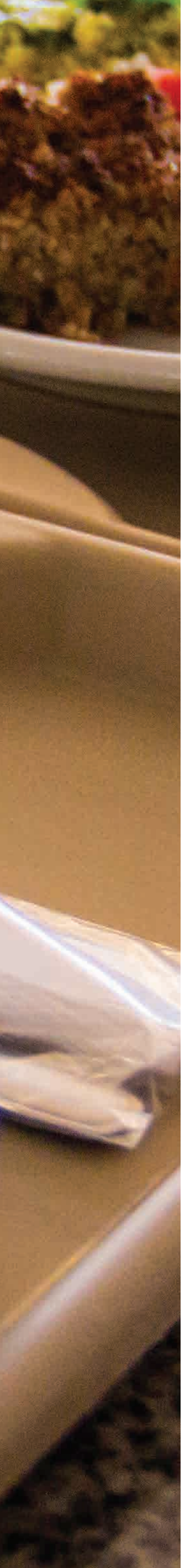

CHAPTER 3. FROM FOODS TO MEALS

The previous chapter of these Guidelines presented general recommendations about food choices aiming at creating diets that are nutritionally balanced, enjoyable, culturally appropriate, and supportive of socially and environmentally sustainable food systems.

Essentially, the basis of such diets consists of a great variety of natural or minimally processed foods, predominantly of plant origin, and of culinary preparations made with these foods Processed foods can be used as long as they are consumed in small quantities and are part of or a side dish in culinary preparations based on natural or minimally processed foods. Ultra-processed foods should be avoided.

This chapter offers specific guidance for the Brazilian population on how to combine foods in the form of meals. As will be detailed in the first section of the chapter, this guidance is based on the dietary patterns of Brazilians who give priority to natural or minimally processed foods in their meals. The following section provides examples of meals consumed by these Brazilians, and the final section shows how these examples can be varied.

BRAZILIAN DIETARY PATTERNS

The characteristics of Brazilian dietary patterns described below are the result of special analyses of the official national Household Budget Survey (HBS) conducted by the Brazilian Institute of Geography and Statistics (IBGE) between May 2008 and May 2009. These analyses were a part of the work done in preparation of these Guidelines.

The 2008–2009 HBS made a detailed study of the diet of a national representative sample of Brazilians aged 10 or older. The sample was drawn from all the regions of the country and has included urban and rural areas and all socio-economic classes. All the foods consumed by over 30,000 individuals during two days of the week, at home and also out of the home, were carefully registered.

Although previous studies conducted by IBGE indicate increasing consumption of ultra-processed foods the 2008–2009 HBS showed that, on average, natural or minimally processed foods and culinary preparations made with these foods still made up almost two-thirds of the total dietary energy intake in Brazil.

Rice and beans alone made up almost a quarter of total dietary energy, followed by beef or pork (red meats), chicken, milk, roots and tubers (mostly cassava and potatoes), fruits, fish, vegetables, and eggs.

Among processed or ultraprocessed foods, those which provide more calories are breads and sandwiches, industrialized cakes, sweet biscuits and sweets in general, sodas, "snack packs", milk drinks, sausages and other processed meats and cheeses.

Analyses of the 2008–2009 HBS, which are of great importance to the recommendations of these Guidelines, indicate that, in Brazil, the share of the diet made of natural or minimally processed foods and culinary preparations of these foods is nutritionally far superior to the share of the diet made of processed foods and ultra-processed foods.

The superiority of the natural or minimally processed foods is particularly evident with regard to nutrients whose content in the Brazilian diet, according to the World Health Organization (WHO) recommendations, is considered insufficient, as in the case of dietary fibre and some vitamins and minerals, or excessive, as in the case of sugar and saturated fats and trans fats.

The 2008–2009 HBS also

reveals that one fifth of the Brazilian population (or about 40 million people of all ages) still widely base their meals on natural or minimally processed foods. These Brazilians, who consume at least 85% of their daily energy from natural or minimally processed foods and dishes and meals based on these foods, nearly meet WHO recommendations for the consumption of proteins, fats (several types), sugar, and dietary fibre. Furthermore, their content of vitamins and minerals is most often well above the average level in Brazil.

Small changes in the diets of the Brazilians who eat most natural or minimally processed foods — such as eating more vegetables and less red meat — would render the nutritional profile of their overall diet practically ideal. The diets of these Brazilians will be used as the basis for the meal options shown in the following section of this chapter.

HEALTHY MEAL OPTIONS

This section describes examples of healthy meals. The examples provided were all extracted from the groups of Brazilians whose consumption of natural and minimally processed foods and their culinary preparations correspond to at least 85% of the total energy intake. One attempt was made to represent in the examples both genders, all age groups (age 10 or above), all classes of income, urban and rural settings, and the five big regions of the country.

The examples are of the three main meals of the day: breakfast, lunch, and dinner. Among the Brazilians who base their meals on natural or minimally processed foods, these three meals provide about 90% of the total calories consumed throughout the day.

Upon selecting examples, to meet the desired regular consumption of vegetables (poorly consumed in Brazil), lunches and dinners where there was a presence of at least one of these foods were selected. On the other hand, red meats (excessively consumed in all of Brazil) appear in only one third of the selected lunches and dinners.

The meals illustrated here are of course not in any

sense 'set menus' or rigid recommendations. Variations among the combinations presented are crucial. These variations substituting types of foods with similar nutritional composition and culinary use (such as replacing beans for lentils or chickpeas, potatoes for cassava or yam, and okra for eggplant or pumpkin) render the meal even healthier, because varieties within the same food group imply a greater diversity in the supply of nutrients.

The variations among the foods of the same group are also pleasing to the senses: they create diversity of flavours, aromas, colours, and textures. They also allow for regional and personal preferences.

There is no emphasis on the amount of each food or the total calories in each meal. This omission is deliberate since the nutritional needs of people, particularly with regard to calories, are very variable depending on age, sex, size (weight and height), and level of physical activity. Furthermore, there is considerable variability between people regarding how they distribute their foods through their daily meals. The control of body weight (not the counting of calories) is a simple and efficient way to determine whether the amount of food being consumed is suitable.

BREAKFAST

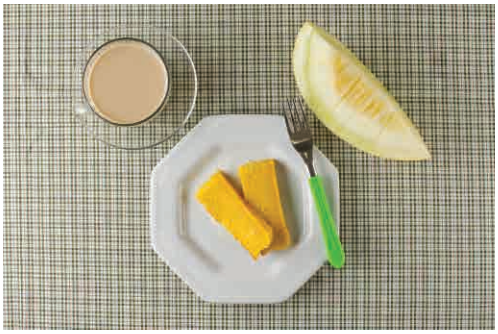

Coffee and milk, corn cake, and melon

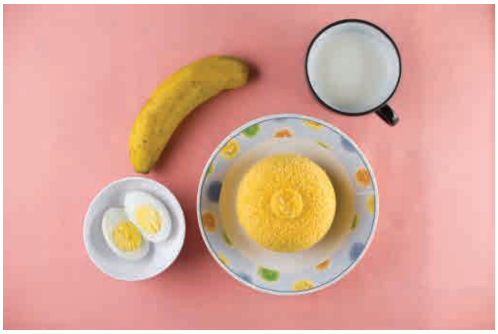

Milk, couscous, eggs, and banana

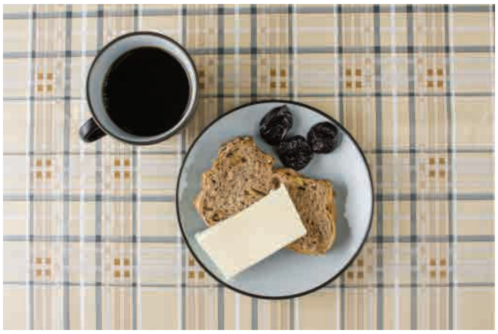

Coffee, whole wheat bread, cheese and prune

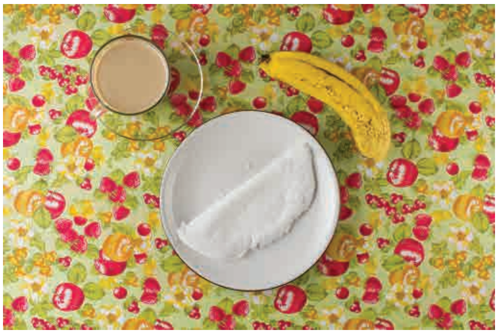

Coffee with milk, tapioca (made from cassava), and banana

Here are shown the breakfasts of eight Brazilians selected among those who base their meals on natural or minimally processed foods.

Fruits and coffee with milk are a constant part of the first meal of the day. With regard to other foods, there is a great variety, with several culinary preparations based on cereals or cassava and, in one of the examples, the consumption of eggs. The variety reflects regional preferences shown through the consumption of tapioca, corn couscous, and corn cake.

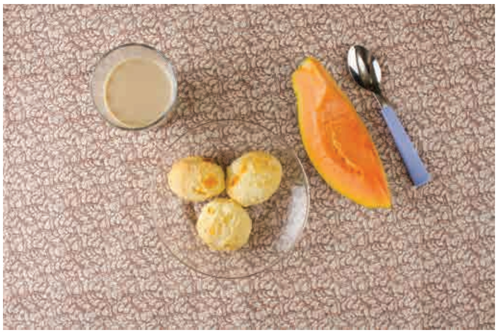

Coffee and milk, cheese bread and papaya

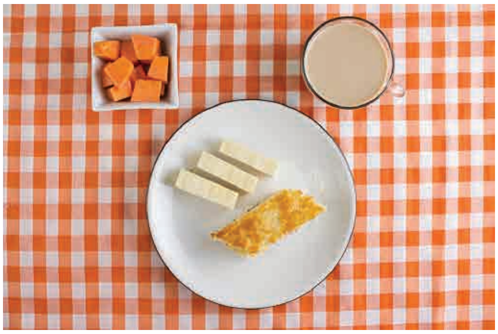

Coffee and milk, cassava cake, cheese and papaya

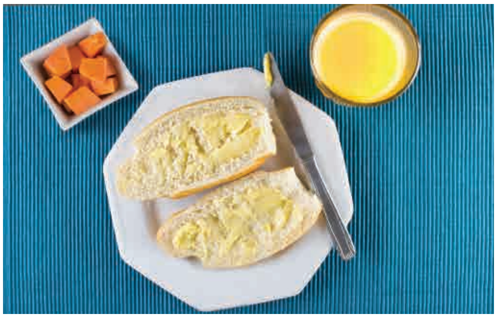

Orange juice, French bread with butter and papaya

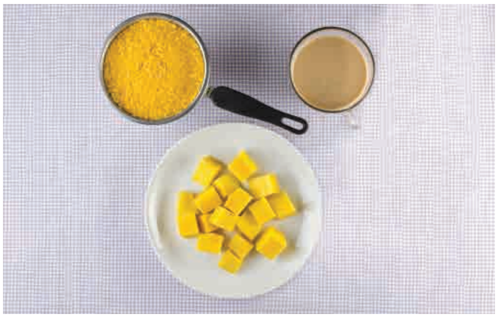

Coffee and milk, couscous and mango

Breads and cheese are part of breakfast in some of the examples, showing how processed foods can be integrated into meals based on natural or minimally processed foods.

LUNCH

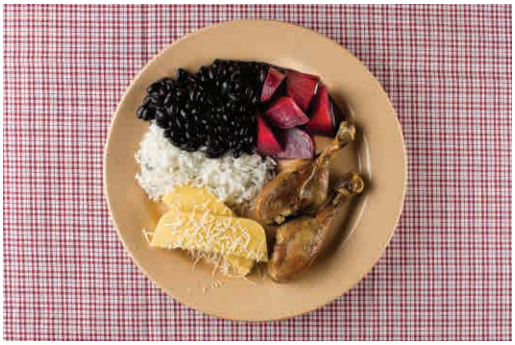

Rice, beans, baked chicken leg, beetroot and cornmeal with cheese

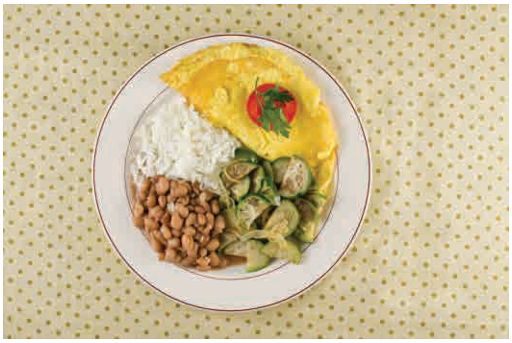

Rice, beans, omelette and sautéed jilo

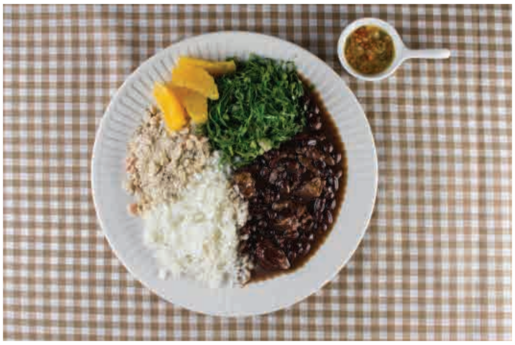

Feijoada, rice, onion and tomato vinagrette, cassava flour, sautéed cole and orange

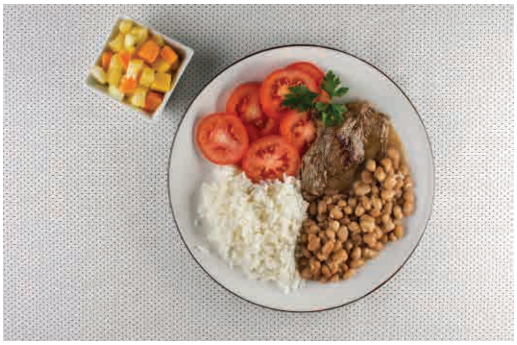

Tomato salad, rice, beans, grilled beef and fruit salad

Here are shown the lunches of eight Brazilians selected as above.

The combination of rice and beans is present in almost all the selected lunches. This reflects a basic aspect of the eating habits of the great majority of Brazilians who opt for natural or minimally processed foods – and indeed, most of the Brazilian population in general.

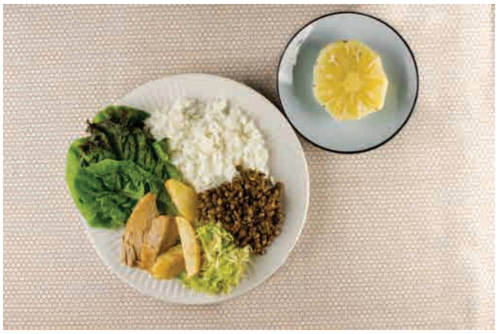

Lettuce, rice, lentils, gammon, potatos, cabbage and pineapple

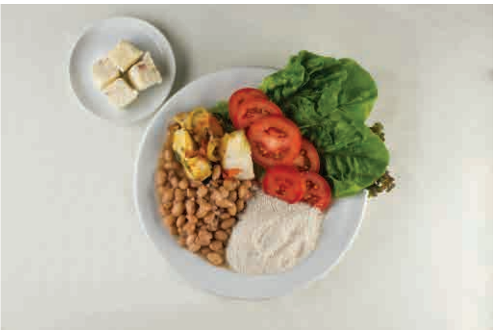

Lettuce, tomato, beans, cassava flour, fish and coconut sweet

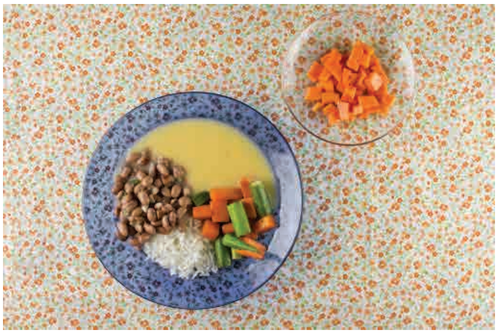

Rice, beans, corn angu, pumpkin, okra and papaya

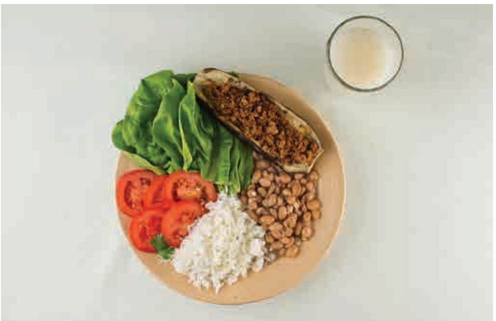

Lettuce and tomato, rice, beans, eggplant and cupuacu juice

In one example, lentils are used instead of beans. In another example, beans are served together with cassava flour (not rice). In two examples, preparations based on corn (corn mush and polenta) are served together with rice and beans.

As mentioned before, vegetables are present in all the examples of lunch, although this is not a common practice in Brazil. To demonstrate the possibilities of increasing and diversifying the consumption of vegetables, examples were sought where different varieties of these foods (lettuce, tomatoes, onions, kale, cabbage, beetroot, squash, okra, eggplant, scarlet eggplant, courgette) are prepared in different ways, like raw, in salads, or in dishes cooked in different ways.

Red meats (beef or pork) are restricted to one third of the meals presented, giving priority to lean cuts and grilled or roasted preparations.

Aiming to present food choices for replacing red meats, meals were selected where there was the presence of grilled, roasted, or stewed preparations of chicken or fish, eggs (omelettes), or vegetables (squash with okra).

Fruits and homemade desserts complete the meals. Processed foods are sometimes used as ingredients, as the cheese in corn polenta.

DINNER

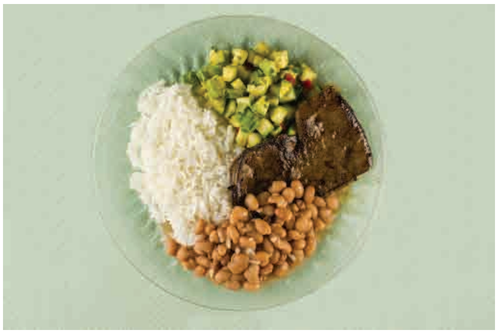
Rice, beans, bovine liver and sautéed zucchini

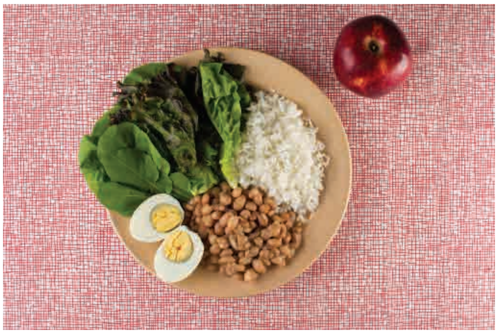
Green leaf salad, rice, beans, eggs and apple

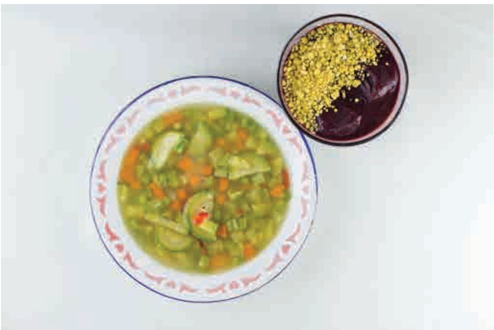
Vegetable soup, cassava flour and acai berry

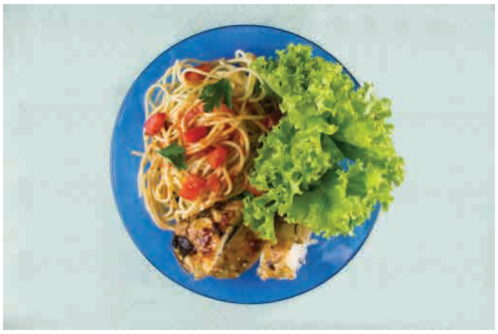
Green leaf salad, pasta and chicken

Here are shown the dinners of eight Brazilians selected as above.

As with the lunches, most of the dinners include rice and beans. In one example, beans with rice is replaced by cassava flour with açaí, and in another example, by pasta served with chicken.

Vegetables are part of every meal, raw in the form of salads, or cooked and served as a side dish, with rice and beans, or within soups.

Red meats are used again in one third of the meals. For the remaining meals, chicken, fish, eggs, and several types of preparations of vegetables serve as options for replacing red meats.

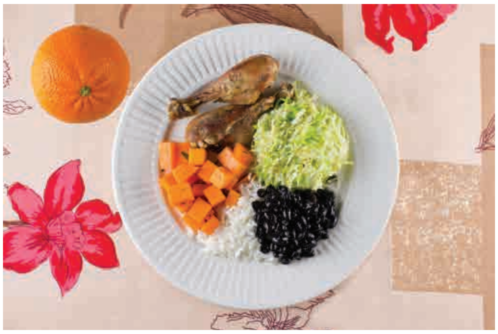

Rice, beans, chicken leg, cabbage, pumpkin and orange

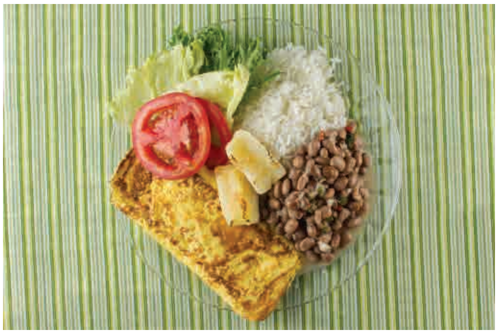

Lettuce, tomato, rice, beans, omelette, and cassava

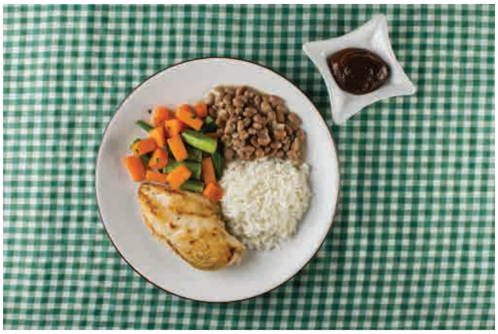

Rice, beans, chicken breast, pumkin with okra and jenipapo jam

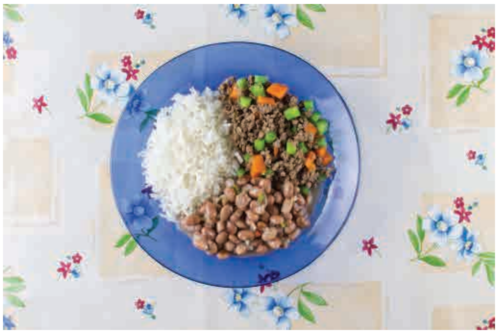

Rice, beans, ground meat with vegetables

Fruits are desserts or part of the dinner, as with açaí mixed with cassava grits.

The appropriate use of processed foods to complement (and not replace) meals that are prepared using natural or minimally processed foods is exemplified by the jenipapo jam dessert.

SMALL MEALS

In addition to the main meals (breakfast, lunch, and dinner), some people might find the need to eat or simply have the habit of eating at other times of the day. Children and teenagers, because of being in their growing phases, usually require one or more small meals. However, the same need might also occur with people during other phases of their lives.

In the case of small meals, the choice of foods should also follow the general recommendations of these Guidelines: opting for natural or minimally processed foods, limiting the consumption of processed foods, and avoiding ultra-processed foods. Fresh or dried fruit are excellent alternatives, as well as milk, yogurt, or nuts. These foods have high nutrient content and great power of satiety, in addition to being practical to carry and consume.

It is very important to plan ahead what will be consumed during small meals, especially when not at home, for example at work or at school. To refrain from consuming ultra-processed foods, due to lack of options,

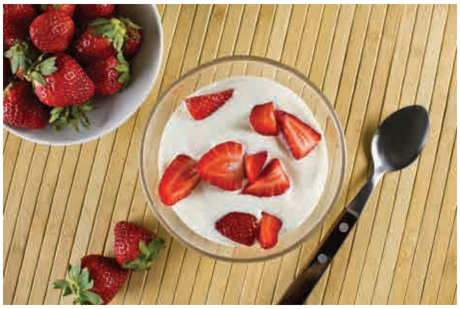

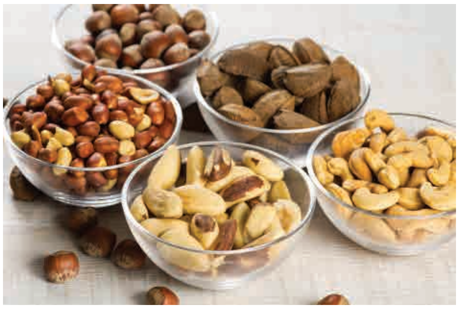

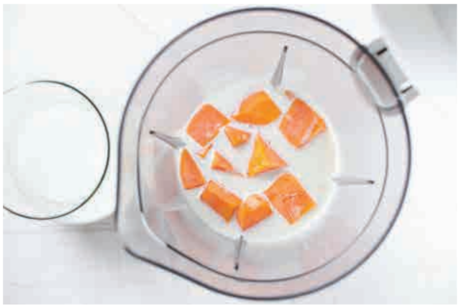

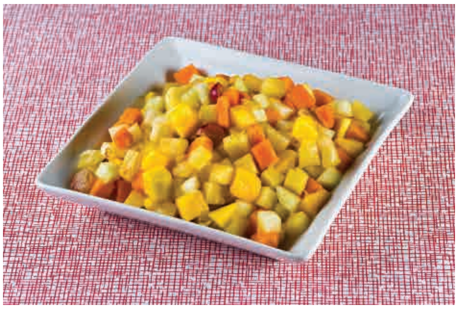

pack and take fresh or dried fruits, or natural or minimally processed foods, or a favoured dish.

FOR MORE OPTIONS

As previously said, the meals exemplified in this chapter show options for combining foods that can be transformed into many other options with the substitution among foods that belong to a same food group.

To assist in making meals, here below are the major food groups commonly consumed in Brazil. Each group includes varieties of foods that have similar culinary use and nutritional profile. Foods within each group are listed, with varieties, culinary uses, and nutrient composition.

BEANS

Chickpea in salads

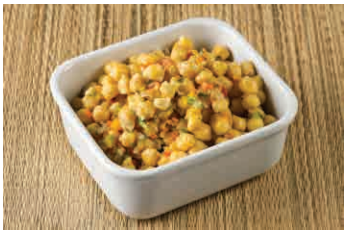

Black beans

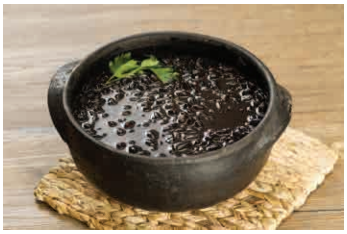

This group includes several types of beans and other foods from the group of legumes, such as peas, lentils, and chickpeas.

There are many varieties of beans in Brazil: black, white, mulatinho, carioca, black-eyed peas, broad beans, cowpeas, among many others. Other legumes, which have similar nutritional properties and culinary uses, are peas, lentils, and chickpeas. Varying the choices of beans and other legumes is good nutritional practice and makes meals more varied and enjoyable.

The combination of rice and beans is the most popular in the country; however, there are many other preparations made with beans that are appreciated by Brazilians, such as tutu à mineira, black-eyed peas, feijoada (bean and meat stew), bean soup, acarajé, among

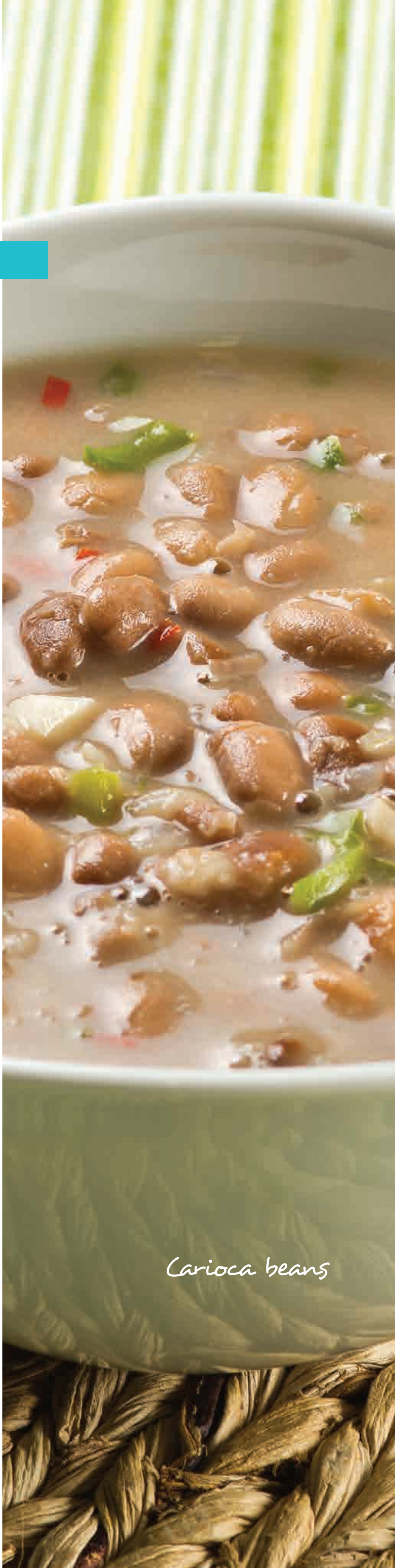

Carioca beans

many others. Cooked white beans, black-eyed peas, peas, lentils, and chickpeas are also consumed in salads.

The preparation of beans (and other legumes) can be quite time-consuming. Strategies that shorten the cooking time include washing the beans and letting them soak in water for a few hours before cooking (discard the water in which the beans are soaked and use fresh water to cook it). Another good alternative is to cook the beans in a pressure cooker. Furthermore, beans that are cooked in a greater quantity during a single preparation can be stored in the freezer for use in preparations throughout the week.

As in all food preparations, special attention should be given to the use of oil and salt in the preparation of beans. Use any standard vegetable oil— soy corn, sunflower, or others—but always in the smallest possible amount so as to not excessively increase the dietary energy of the preparation. The same guidance applies to the amount of salt. Keep it to a minimum in order not to render the content of sodium excessive.

To reduce the amount of oil and salt added to the beans, and the occasional use of salted meat, prepare the beans with generous amounts of onions, garlic, and favoured herbs and spices like bay leaves, parsley, chives, pepper, or coriander. If you cook the beans with other foods such as carrots and green beans, they will also add flavour and colour to the preparation.

Beans, as well as all other legumes, are sources of proteins, dietary fibre, vitamin B complex, and minerals, such as calcium, zinc, and iron. The high content of fibre and the moderate amount of calories per gram, make these foods satisfying, which checks overeating.

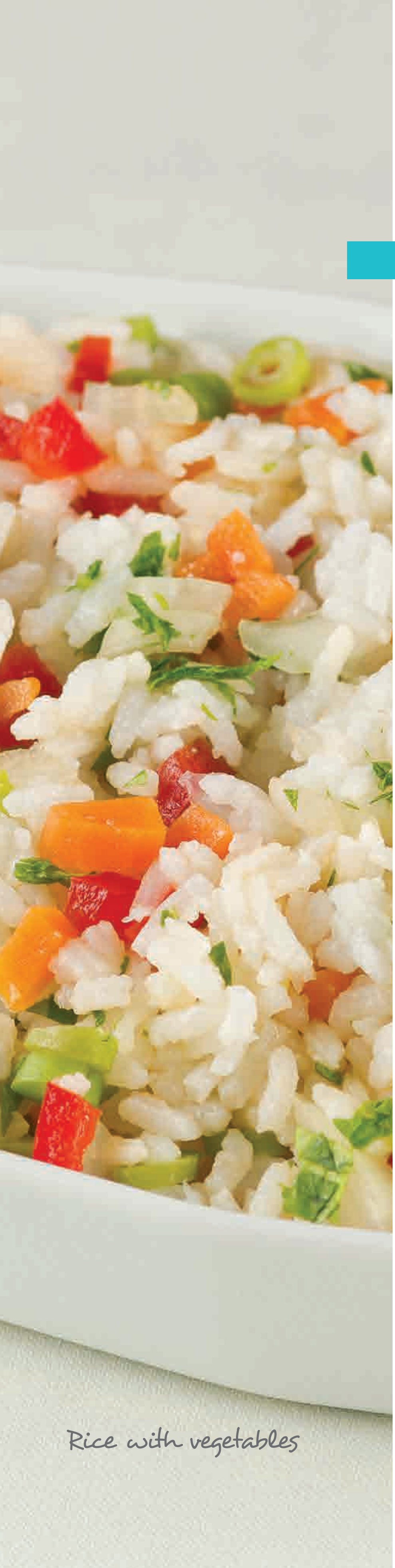

Rice with vegetables

CEREALS

Cornmeal with tomato sauce

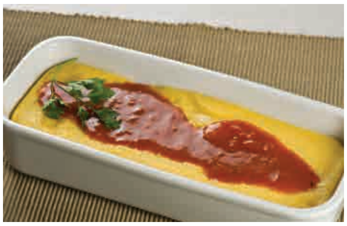

Pasta with tomato and fresh herb sauce

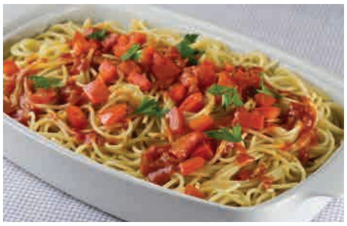

This group includes rice, corn (including grains, grits and flour), wheat (including grains, grits, flour, and also pasta and breads), and other cereals, such as oats and rye.

RICE

Rice is the main cereal in Brazil. It is most commonly served with beans, but, being an extremely versatile food, it is also consumed with vegetables, eggs, and meat, as well as with several types of risotto, Greek rice, cuxá rice, carreteiro rice, galinhada, and Maria Izabel. It is also the basis of arroz doce, a traditional Brazilian dessert.

As with beans, only small amounts of oils and salt should be used in the preparation of rice, which can be made more delicious with garlic, onion and herbs.

CORN

Corn (maize) is also an important cereal in Brazil. It is eaten in the form of cooked whole cobs or kernels, or in the form of creams or soups. Corn dishes include porridges and desserts, such as canjica, mungunzá, porridge, mingau (gruel), curau and puddings. Corn flour used to make couscous, polenta, farofa, corn cake, corn mush, fish sauce, and xerém, consumed at lunch and dinner and, in some regions, also at breakfast.

WHEAT

The wheat consumption in Brazil is mainly through flour. However, wheat grain is sometimes used in salads, in hot preparations with vegetables, or in soups (wheat canjica). Tabule (broken wheat salad) is prepared with olive oil, tomatoes, onions, mint, and parsley.

Wheat flour has multiple culinary uses in Brazil. With vegetables, eggs, and meat, and added oil and salt, it is used to prepare savoury pies. Sweet pies and cakes are prepared with wheat flour, oil, sugar, milk, eggs, and fruits. Homemade breads are made with wheat flour, water, salt, and yeast, which is used to ferment the flour. Wheat flour is also used for breading meats and vegetables.

As described in these Guidelines, all types of pasta made from wheat flour and water (with or without the addition of eggs) are culinary preparations when made at home and are minimally processed foods when industrially produced.

Quick preparation and many types and uses are the main culinary features of pasta. Several types of pasta are eaten simply with garlic and olive oil or with sauces made from tomatoes, courgettes, ground meat, and many other foods. Adding grated cheese to pasta preparations is very common and this is a good example of the appropriate integration of processed foods into freshly prepared dishes and meals.

The so-called “instant pasta” (or instant noodles) is a typical ultra-processed product, as can be seen by reading the long list of ingredients on the product label. Therefore, its consumption should be avoided.

As also described in these Guidelines, industrialised breads

can be processed or ultra-processed. They are processed when they are made with the same ingredients used in the preparation of homemade breads. As processed foods, they should be consumed in small quantities and as part of meals with natural or minimally processed foods.

Breads that, in addition to flour, water, salt and yeast, contain other ingredients such as hydrogenated vegetable fat, sugar, starch, whey, emulsifiers, and other additives are ultra-processed foods and as such should be avoided.

Rice, corn, wheat, and all cereals are important sources of carbohydrates, dietary fibre, vitamins (especially B complex), and minerals. Combined with beans or other legumes, cereals are also an excellent source of quality proteins.

White rice and white wheat flour have lower amounts of dietary fibre and micronutrients, which are lost in the refining process. Less processed versions of these foods, such as brown rice and whole-wheat flour, are preferable. Parboiled rice (husked and polished after being immersed in water) is also a good alternative, being close to wholegrain rice in nutritional quality, and having an aroma, flavour and texture similar to white rice.

ROOTS AND TUBERS

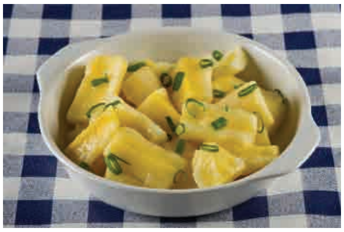

Boiled cassava with chive and or parsley

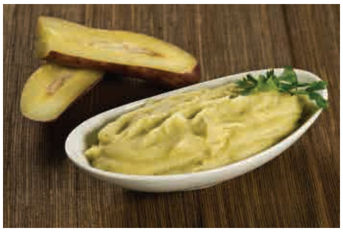

Sweet potato purée

This group includes cassava, potatoes, sweet potatoes, baroa potatoes, inhame (taro), and cará (water yam).

Roots and tubers are very versatile foods and can be eaten cooked, roasted, in stews, or as purees. Brazilians often consume them at lunch and dinner, along with beans and rice, vegetables, and meats. In some regions of Brazil, cassava and sweet potatoes are consumed at breakfast as bread substitutes. Cassava is also used in the preparation of homemade desserts including cakes and puddings.

Cassava consumed in the form of grits or flour is frequently a side dish with fish, vegetables, açaí and several other foods. Cassava flour is also used as an ingredient in recipes for porridge, couscous, tutu, drover beans, and farofa. In the North and Northeast regions of the country, it often replaces rice when eaten with beans.

The starch extracted from cassava is used for the preparation of tapioca and pão de queijo. In some regions of Brazil, tapioca replaces bread for breakfast.

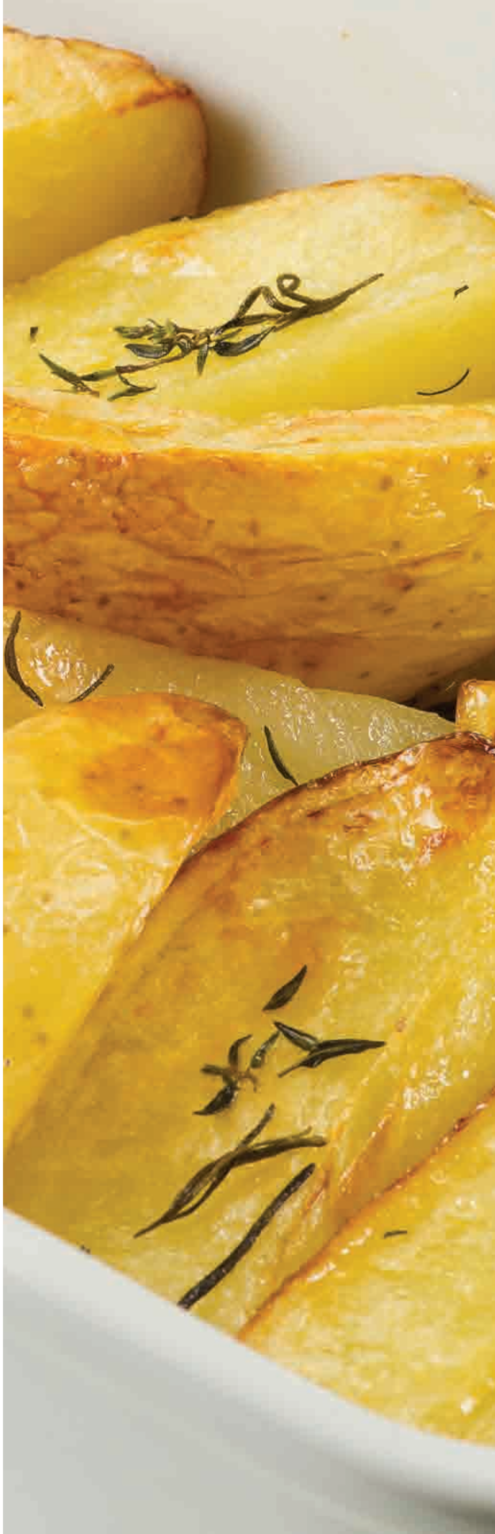

Cooked potato with rosemary

As with all foods, roots and tubers should be prepared and cooked with only small amounts of oil and salt. They are best boiled or baked because when fried they absorb a lot of oil or fat. Again, plentiful use of peppers, onions and garlic, and herbs, make root and tuber preparations more delicious.

Roots and tubers are also rich sources of complex carbohydrate and dietary fibre. Some also are good sources of some vitamins and minerals.

VEGETABLES

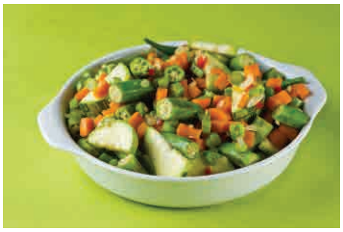

Mix of sautéed vegetables

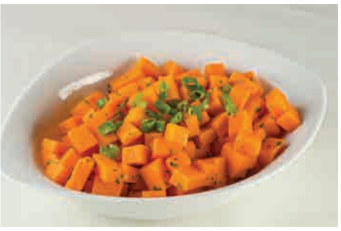

Sautéed pumpkin with onions, chive and or parsley

In Brazil, there is a huge variety of vegetables: squash, zucchini, eggplant, beetroot, carrots, cucumber, bell peppers, okra, tomatoes, onions, garlic, maxixe, jiló, lettuce, chard, watercress, cabbage, endive, broccoli, mustard, chicory, chayote, kale, spinach, gueroba, jurubeba, catalonha, ora-pro-nobis, among others. Varieties within the same type of vegetables are frequent and vary regionally, such as the varieties of squash that are named paulista, japanese, baianinha, pescoço, garota. Lettuce may be americana, romana, roxa (purple), lisa (smooth) or crespa (curly).

Many vegetables are sold throughout the year all over the country. Varieties that are local or in season are cheaper and have more flavour. Organic vegetables and those

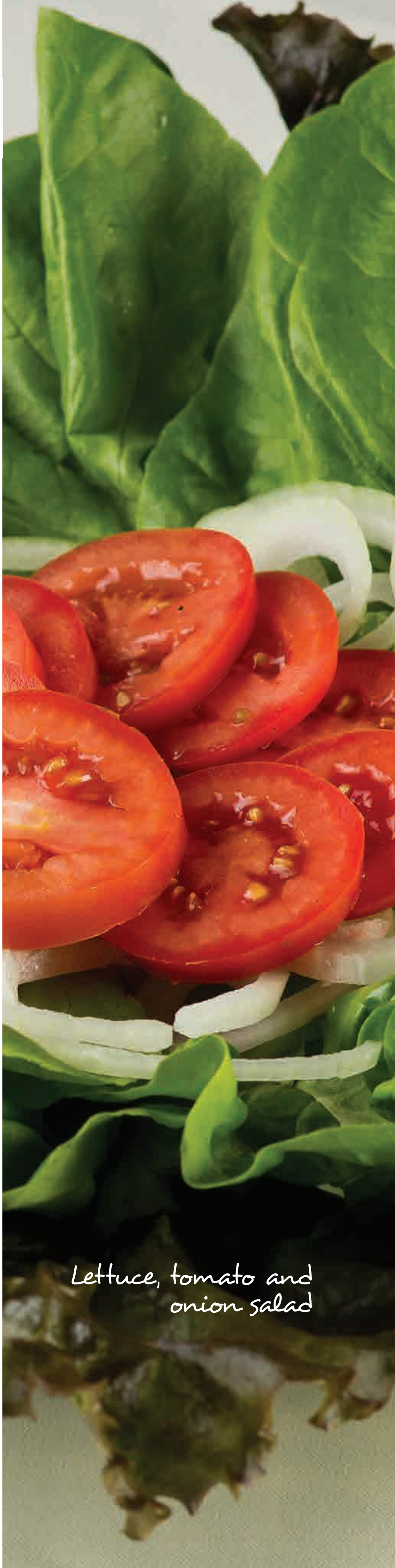

Lettuce, tomato and onion salad

that are produced agro-ecologically are particularly flavoursome, as well as being produced in ways that protect the environment.

Vegetables are consumed as salads, in hot preparations (boiled, sautéed, baked, au gratin, breaded, and stews) or in soups. Some are stuffed, or made into purees, or breaded. Some have more flavour when cooked (like squash) or sautéed (like cabbage). Others are eaten raw, in the form of salads (lettuce, endives, chicory, pepper, cucumber and tomatoes, as examples). Others such as carrots and onions can be prepared in different ways (boiled, steamed, sautéed, or raw). Vegetables can also be added into rice dishes, sauces and farofas and used as pie fillings.

The recommendation of adding small amounts of salt and oil, and generous use of natural herbs and spices, also applies to vegetables. The use of lemon in salads helps reduce the need of additional salt or oil.

Microorganisms that cause disease can contaminate vegetables; therefore, it is very important to properly sanitize them, especially when eaten raw. Therefore, before being prepared and consumed, all vegetables should be washed in running water and then to be quite safe, placed in a basin with water containing sodium hypochlorite, which can be purchased in supermarkets and retail shops. The label for sodium hypochlorite states the amount that should be used and how long vegetables should be soaked in it. Vinegar solution soaks do not have the same ability to eliminate microorganisms that can contaminate vegetables.

Vegetables are very healthy foods. They are excellent sources of several vitamins and minerals, and therefore very important for the prevention of micronutrient deficiencies. In addition to being a source of dietary fibre, they provide, in general, several nutrients while being low in dietary energy, making them ideal in the prevention of obesity, and chronic diseases associated with this condition, such as diabetes and heart disease. Numerous antioxidants in vegetables protect against some cancers. For their exceptional nutritional properties and extensive culinary versatility, this food group is an excellent alternative to red meat in Brazil.

Vegetables canned or bottled in a solution of water and salt and sometimes vinegar, such as carrots, cucumbers, or onions (as well as peas or potatoes) are processed foods. Like other processed foods, they preserve most of their nutrients as when they are raw but contain excessive amounts of sodium, which is why their consumption should be limited.

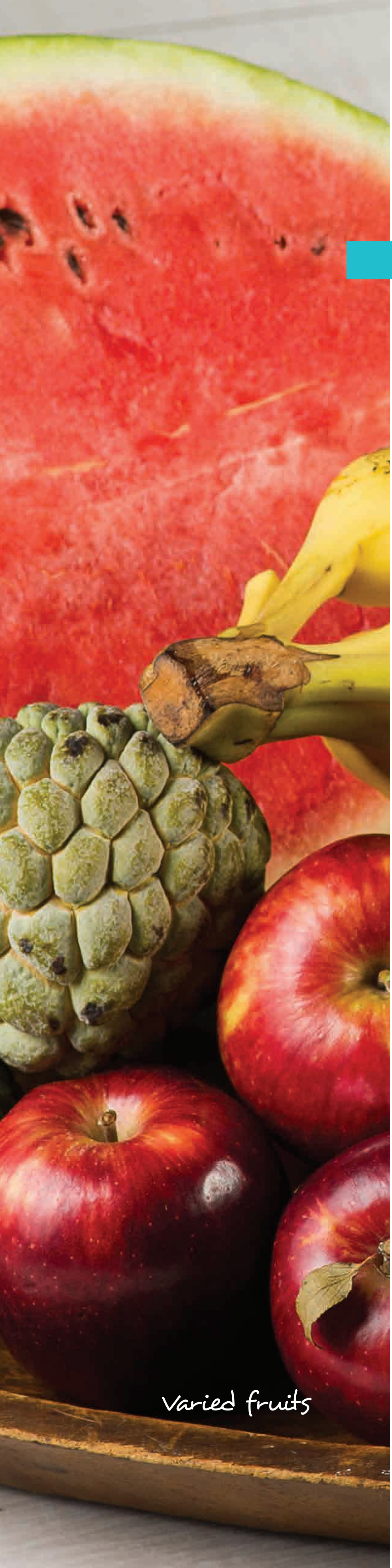

Varied fruits

FRUITS

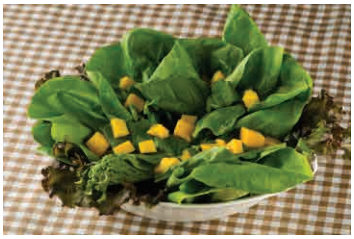

Green leaf salad with mango

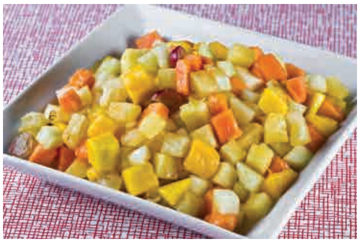

Fruit salad

Brazil has a vast variety of fruits, some found only in or near Brazil, many native, and many others originally imported from other tropical and also temperate countries. In alphabetical order, these include abiu, açaí, acerola, apple, araça, araticum, atemoia, avocado, bananas, bacuri, blackberries, breadfruit, cagaita, cajá, caqui, cacao, ciriguela, cupuaçu, fig, grapes, guava, graviola, jabuticaba, jackfruit, jenipapo, lemon, lime, mountain apples, murici, oranges, papaya, passion fruit, pequi, pineapple, pitanga, pitomba, plums, pomegranates, tamarinds, tangerines.

Like vegetables, fruits that are local or in season are the best choices, as are fruits produced agro-ecologically.

Fruits can be eaten fresh or dried, as part of the main meal or as small meals. They are an important breakfast component, and at

lunch and dinner, they can be eaten in the form of salads or as a dessert. In some regions of Brazil, fruits are consumed with fish and cassava flour (açaí) or with rice and chicken (pequi).

Especially when eaten with the peel, fruits, like vegetables, need to be cleaned.

Like vegetables, fruits are very healthy foods. They are an excellent source of dietary fibre, vitamins and minerals, and several compounds that contribute to the prevention of many diseases.

Fruit juices do not always provide the same benefits of the whole fruit. Fibre and many nutrients may be lost in juice preparation and its satiety power is always lower than the whole fruit. Therefore, it is always best to consume the entire fruit.

Whole fruits with added sugar, such as candied fruits and canned fruits in syrup, are processed foods. As such, they retain most of their nutrients, but the processing excessively increases their sugar content. Like other processed foods, they should be consumed in small quantities, as part of culinary preparations, or in meals where natural or minimally processed foods predominate. Candied fruits, for example, can be part of pies and cakes, and fruits in syrup may occasionally be consumed as desserts.

Industrially manufactured juices and fruit-based drinks are generally made from fruit extracts and refined sugar from concentrated grape or apple (consisting primarily of sugar), or artificial sweeteners are added. Frequently, preservatives, flavourings, and other additives are also added. As such they are ultra-processed foods, to be avoided.

NUTS

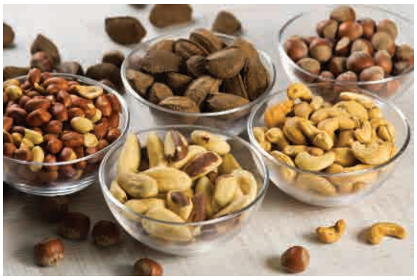

Nuts

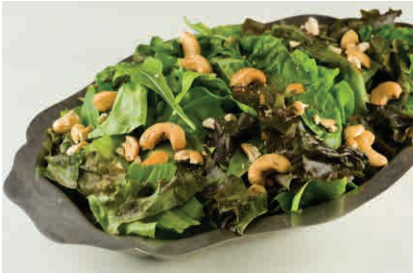

Green leaf salad with cashew nuts

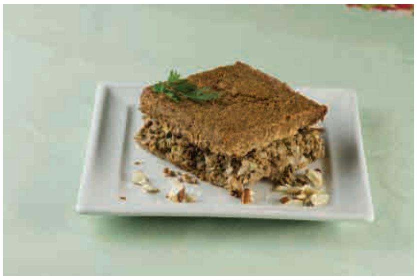

Ground beaf kibbeh with walnuts

Nuts, as understood in these Guidelines, include cashew, baru and Brazil nuts, as well as walnuts, peanuts, and almonds.

Nuts have many culinary uses. They are used as ingredients in salads, sauces, and various savoury and sweet dishes (farofas, paçocas, pé de moleque) and are also added to fruit salads. Since they require little or no preparation, they are excellent choices for small meals.

Nuts contain plenty of dietary fibre, vitamins, minerals and other protective bioactive compounds, and are also high in healthy (unsaturated) fats.

Nuts with added salt or sugar become processed foods. As such, their consumption should be limited.

MILK AND CHEESE

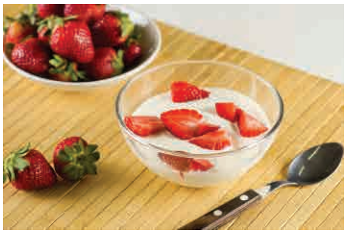

Natural yogurt with fruits

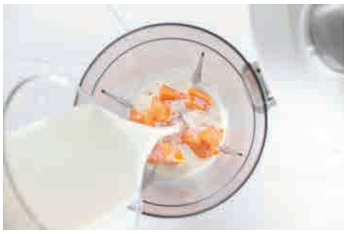

Papaya smoothie with cow milk

This group includes minimally processed foods such as cow's milk, cheese curds, plain yogurt, and processed foods such as cheeses.

In Brazil, cow's milk is often consumed pure, with fruit or with coffee in the first meal of the day. It is also an ingredient in creams, pies, cakes, and other sweet and savoury dishes. The consumption of natural yoghurt and other fermented milk foods is increasing in Brazil. Cheeses are mainly consumed as part of dishes based on minimally processed foods, as in pasta with tomato sauce or polenta made with corn flour.

Milk and plain yoghurt are good sources of proteins, and some vitamins and minerals

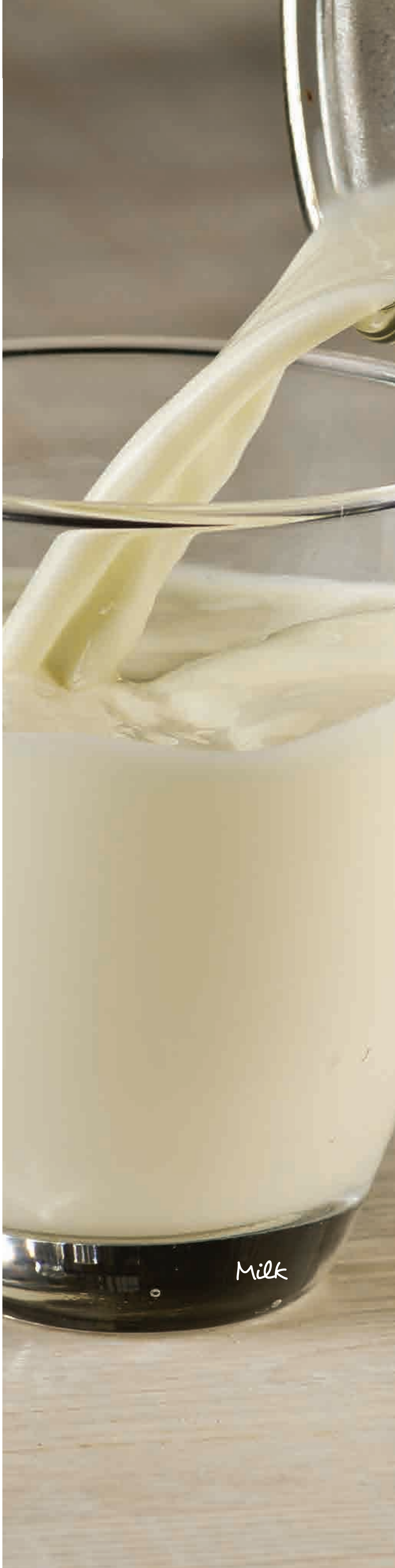

Milk

(in particular vitamin A and calcium). When they are whole, they are also high in fat, particularly unhealthy fats (saturated fat). Versions without fat or with less fat (skimmed or semi-skimmed) may be more appropriate for adults.

Cheeses are also rich in proteins, vitamin A, and calcium. However, besides the very high content of saturated fats, they are products with high energy density(due to loss of water during processing) and high sodium concentration (due to the addition of salt). Therefore, cheeses, like all processed foods, should always be consumed in small amounts as part of culinary preparations or as side dishes in meals with mainly natural or minimally processed foods.

Milk drinks and yogurts that have been sweetened, coloured, and flavoured are ultra-processed foods, and as such should be avoided.

MEAT AND EGGS

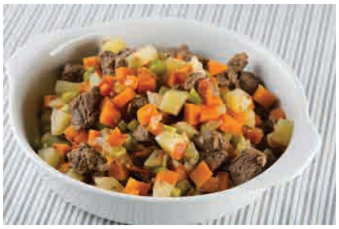
Meat stew with potatoes and vegetables

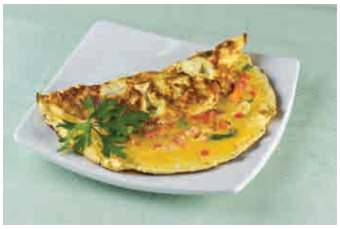
Vegetable omelette

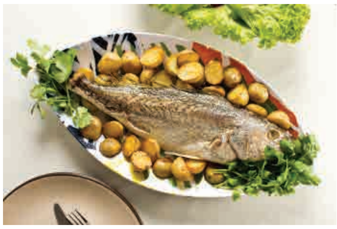
Cooked fish

This group includes beef, pork, goat and lamb (all known as red meat), poultry and fish, and all types of eggs. Various types of meats and eggs are commonly consumed in Brazil with rice and beans or other foods of plant origin; they are valued because of the flavour they add to the meal. They also have in common the fact that they are rich in proteins, vitamins, and minerals. Red meat, poultry, fish, and eggs are considered separately in this section.

RED MEAT

Beef and pork, as well as other red meats, are greatly appreciated in Brazil and are eaten very often in all regions of the country. Some red meat cuts are eaten grilled with salt. Others are used to make stews or are consumed with baked potatoes, cassava, vegetables, and assorted seasonings; they can also be ground to prepare sauces or toppings. The best way to prepare fatty cuts is by roasting, grilling, or sautéing, while less fatty cuts may be used in stews.

As with the foods above, red meat should be prepared with the least possible amount of oil and salt. One way to reduce the use of salt in the meat seasoning is to use herbs such as thyme, sage, and rosemary.

All red meats are excellent sources of high-quality proteins and many have a high content of micronutrients, particularly iron, zinc, and vitamin B12. However, they tend to be high in fats, particularly saturated fats, that when consumed in excess, increases the risk of heart disease and numerous other chronic diseases. High consumption of red (and processed) meats also increase the risk of bowel cancer.

POULTRY

Poultry, especially chicken, is also enjoyed throughout Brazil. Poultry is part of traditional Brazilian cuisine such as in galinhada mineira, galinhada goiana, galinha guisada, galinha à cabidela and pato no tucupi.

Poultry cuts are also prepared in various ways. Cuts with more fat, such as drumstick, thigh, and wings should be baked or grilled and those with less fat can be boiled or stewed. As with other foods, the use of natural seasonings reduces the amount of salt added to poultry cuts.

Poultry, like red meat, is a good source of proteins and vitamins and minerals. But it is also high in unhealthy fats. However, the fat in poultry is mostly in the skin, which is best discarded.

FISH

Although Brazil has a large coastline and numerous large rivers, in most regions fish supply is very small and prices are relatively high compared with those of red meats and poultry. This helps to explain the low fish consumption frequency in the country.

Fish are eaten baked, grilled, stewed (moqueca), or cooked. Their juices can be used to make pirão (a thickened fish sauce made also with cassava flour) or serve as a filling for pies. Culinary preparations of fish with vegetables like peppers, tomatoes, and

onions or with fruits like banana and açai are classics in regional cuisines.

Similar to red meats and poultry, fish are rich in high-quality proteins, and many vitamins and minerals. Because of the low fat content and because they have a high proportion of healthy fats (unsaturated fat), fish, as well as vegetables, are excellent substitutes for red meats.

EGGS

Unlike fish, eggs, especially chicken eggs, are relatively cheap and affordable in Brazil. They are extremely versatile and can be eaten boiled, scrambled, fried, or used as ingredients in omelettes, soufflés, and several other culinary preparations. They combine well with vegetables like carrots, cauliflower, spinach, chayote, broccoli, courgette, and maxize. They are also used with milk and sugar in the preparation of cakes, puddings, and pastries.

Similar to meat and fish, eggs are rich in high-quality proteins, minerals, and vitamins, especially B complex, and are also a good substitute for red meat.

For diverse reasons, some people choose not to consume animal foods; they are known as vegetarians. The restriction may be only for meat or might include eggs and milk or even all foods of animal origin.

Although the consumption of meat and other foods of animal origin or any other food group is not absolutely essential for a healthy diet, the restriction of any food obliges one to pay greater attention when choosing a food combination that will be part of the diet. The level of restriction will establish the level of attention required and need of advice from a dietician.

Specific vegetarian guidance, as well as specific guidelines for other types of food restriction, such as for milk or wheat consumption, are not addressed in these Guidelines. However,

the general recommendations to give priority to fresh and minimally processed foods, and avoid ultra-processed foods, apply to everyone, including vegetarians.

WATER

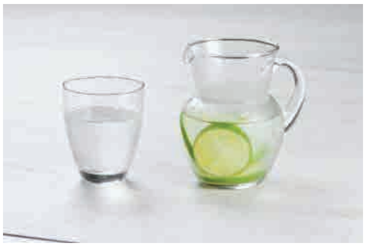

Water with lemon

Water is essential for the maintenance of life. Without water, humans cannot survive for more than a few days. Three-quarters of the body weight of infants, and more than half of the weight of adults, is water.

As with food, the amount of water that is needed daily is very variable and depends on several factors like age and weight, the amount of physical activity, and the climate and temperature. For some people, two litres of water per day may be sufficient; others will require three or four litres or even more, as in the case of athletes.

An important fact is that humans can efficiently regulate their daily water balance so that, throughout the day, the water intake corresponds to water that has been used or eliminated by the body.

The daily water balance is controlled by sophisticated sensors located in the brain and in different parts of the body. These sensors induce the sense of thirst and induce drinking of water when intake is not sufficient to replenish water used or eliminated. It is very important to pay attention to the first signs of thirst and to drink plenty of water.

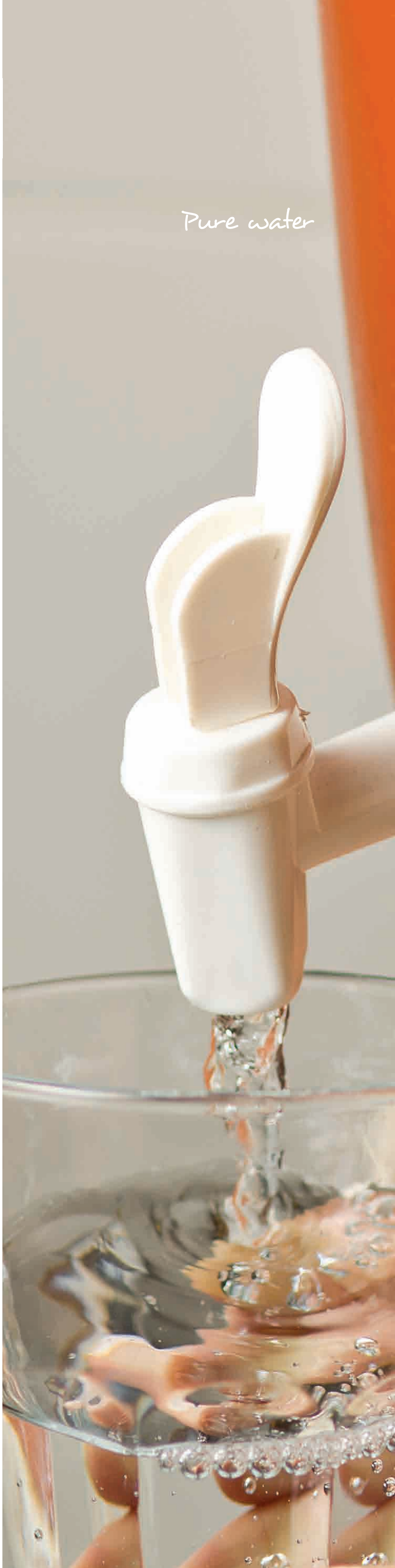

Pure water

Water drunk should be from water as such, and also as contained in natural or minimally processed foods and culinary preparations made with these foods. All the water used for drinking and used in culinary preparations needs to be fit for human consumption; free of microorganisms and chemicals that might endanger human health. The water provided by the public water supply should meet these criteria. When in doubt, water should be filtered and boiled before being drunk. .

Pure water (or, as preferred by some people, “seasoned” with lime slices or mint leaves) is the best option. Brazilians also consume water in the form of coffee and tea, in which case sugar should be reduced to a minimum or not added at all.

To protect the environment, non-disposable utensils such as glass cups or mugs should be used when drinking water, coffee, or tea. When away from home, it is wise to keep at hand bottles or canteens with fresh water.

Most natural or minimally processed foods have high water content. Milk and most fruits contain between 80% and 90% water. Vegetables typically have more than 90% of their weight in water. Pasta, potatoes, and cassava contain about 70% water after cooking. A plate of rice and beans is made up of two-thirds water. When diets are based on these foods, they provide about half the total amount of water needed.

Unlike natural or minimally processed foods, processed and ultra-processed foods generally contain little water and one of the reasons is that they must last longer on shelves. This is the case with packaged salty snacks and biscuits, which usually contain less than 5% water. Ultra-processed foods such as soft drinks and various types of sweetened drinks are almost all water but contain sugar or artificial sweeteners and various additives, and should be avoided.

FINALLY

With the combination of beans, grains, roots, tubers, flour, pasta, vegetables, fruits, nuts, milk, meat, eggs, coffee, tea, and water, readers of these Guidelines may indefinitely multiply the examples of meals presented in this chapter, creating healthy, diverse, and tasty alternatives for breakfast, lunch, dinner, and small meals.

A healthy diet requires some attention concerning the safety of the food consumed. Foods that are not properly sanitised, dirty cutlery, insects, and people themselves can be sources of contamination. To ensure the nutritional quality and avoid risks of infection or poisoning, food must be selected, maintained, and handled appropriately. Guidance is given at the end of this chapter.

CARE IN SELECTION, CONSERVATION, AND FOOD HANDLING

How to select

Food should be purchased from markets, retail shops, butchers, and fishmongers that are clean, organised and in a good state, and that offer good quality options.

Fruits and vegetables should not be chosen or eaten if they are spoiled, mouldy, have strange colours, or altered texture.

Fresh fish should be refrigerated, with well-bonded scales with no cuts, and have rosy gills and bright clear eyes. Frozen fish should be properly packed and stored at appropriate temperatures. Avoid acquiring those that have accumulated ice or water on the package, as they may have been frozen and thawed again.

Meat should not be acquired if it has darkened or greenish colour, unpleasant odour, or altered consistency. Fresh meat is bright red (or light coloured in the case of pork and poultry), has firm texture, and well-adhered and light-coloured fat.

Packaged foods must be within the validity period, packaging must be sealed and free from dents, holes, or bulging areas, and contents should not show changes in colour, smell, or consistency.

How to conserve

Long-lasting foods (rice, corn, beans, flours, oils, sugar, salt, milk powder and some kinds of fruits and vegetables) should be stored in a cool dry place at room temperature away from sunlight. Foods that spoil more easily should be refrigerated (meat, eggs, milk, cheese, butter, most fruits and vegetables) or

frozen (raw meats and poultry). Pre-prepared or leftover dishes and meals should be refrigerated.

How to handle

Care is needed when handling and preparing food. To reduce the risks of contamination, wash hands before handling food and avoid coughing or sneezing on them, avoid consuming raw meats and eggs, wash fruits and vegetables under running water and for greater safety disinfect them appropriately, and keep food protected in packaging or containers.

Kitchens should be clean, airy, and organised. Refrigerators, the stove, cabinets, the floor and the walls should be cleaned regularly, with food cleared out of the way.

As well as safety considerations, kitchens that are clean, light and organised are more efficient and also attractive places to be and to socialise.

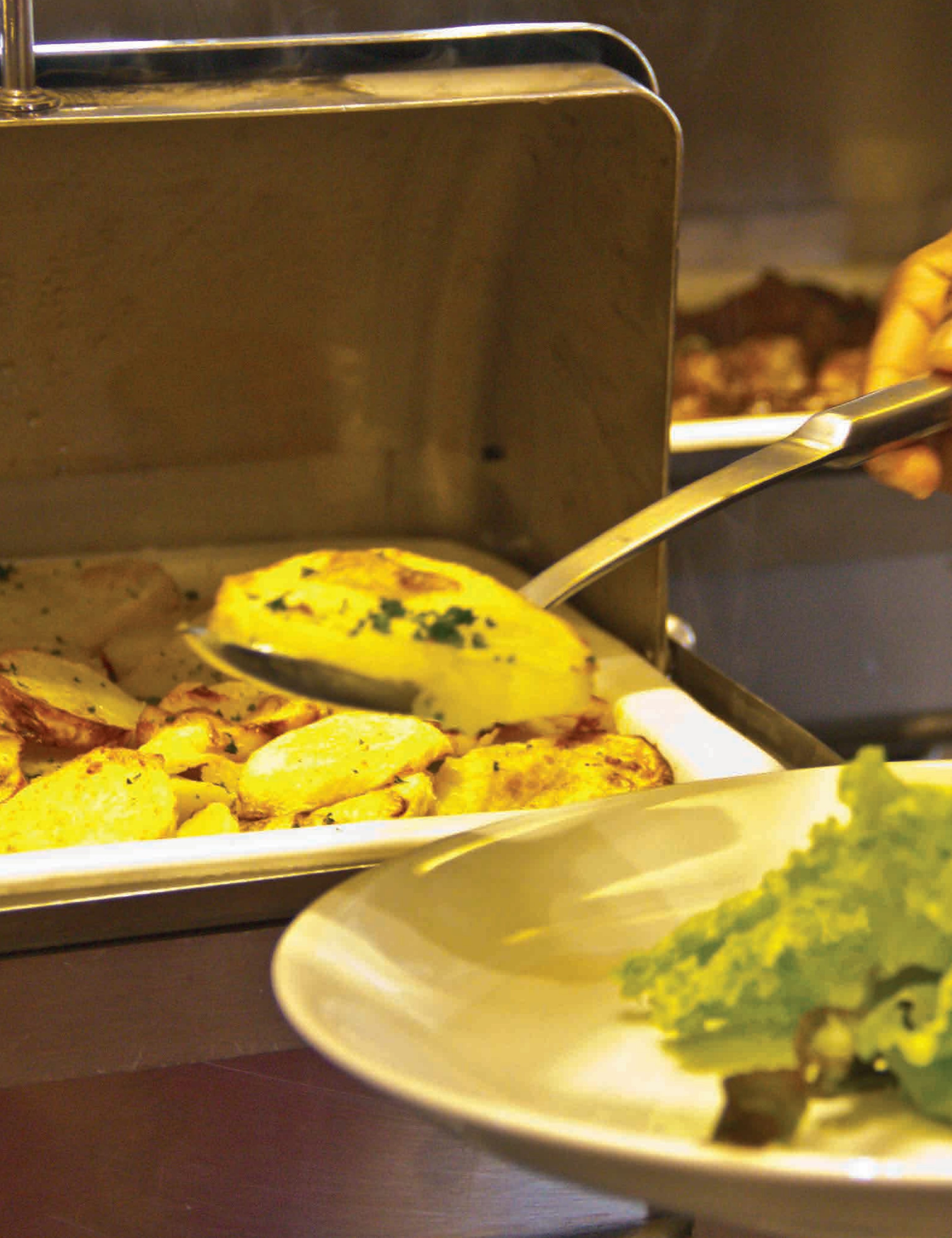

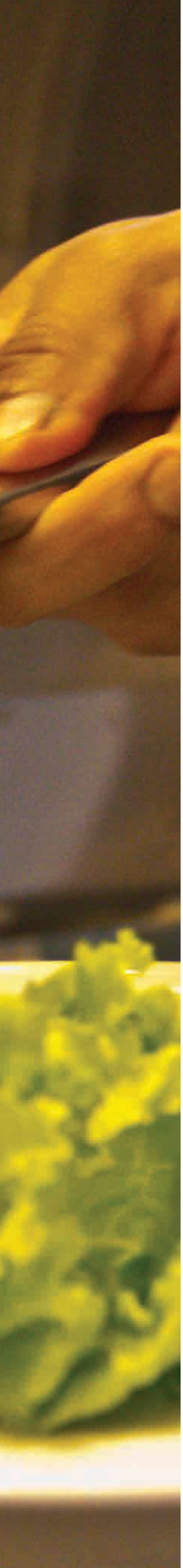

CHAPTER 4.
MODES OF EATING

The previous chapter has shown how to combine foods to make healthy, satisfying and enjoyable meals for breakfast, lunch, and dinner, and also for small meals. This chapter is about the context of eating. Three aspects are considered: the time and attention devoted to eating, the environment where it occurs, and the sharing of meals. Following the recommendations of this chapter will result in several benefits. These include improved digestion and use of foods, more efficient control of what and how much food is consumed, enhanced family and social life, and, in particular, more pleasure in eating.

EATING REGULARLY AND CAREFULLY

Always when possible, eat daily meals at similar times. Avoid 'snacking' between meals. Eat slowly, with full attention, and enjoy eating without engaging in another activity.

Having meals every day at the same time and consuming them with attention and without being in a hurry, favours the digestion of food and also avoids overeating. The biological mechanisms that regulate appetite are complex, and depend on several stimuli that take some time to signal that enough has been consumed. It follows that eating regularly, slowly, and carefully is the natural way to control how much is eaten.

Often, the need to eat frequently, or to snack often or to 'graze', is stronger in the presence of food, especially when such food is presented as particularly attractive. This is particularly evident when the source of stimulation is ultra- processed products such as confectionery, sweet or salty packaged snacks or other products whose ingredients and formulation give them very intense flavour. It is better not to have these products at hand, when at home, at work, or at school. Good choices of food to have at hand are fresh or dried fruits and unsalted nuts.

Some simple steps can prevent eating quickly or irregularly.

The more time that is taken to chew food, the greater the attention to the act of eating, and the longer it takes to eat a meal. In doing so, all the pleasure afforded by the different flavours and textures provided by natural or minimally processed foods combined with culinary preparations as meals, is enjoyed all the more.

Eating a salad or a soup or broth before the main course of a meal is another way to give the body the time needed for food to be absorbed, which also inhibits overeating.

EATING IN APPROPRIATE ENVIRONMENTS

Always prefer to eat in clean, comfortable, and quiet places, and where there is no stimulus to consume unlimited amounts of food.

The quality of the environment of eating influences what is eaten and in what quantity, and how much it is enjoyed. Scents, sounds, light, comfort, and cleanliness are all important.

Clean, quiet, and comfortable places encourage attention to the act of eating mindfully and slowly, enable meals to be fully appreciated, and decrease overeating.

Distractions such as using cell phones or watching television while eating are unhelpful and unhealthy. So is eating while working, standing, walking, or driving.

It is always best to avoid being faced with lots of food or by large portions. At home or when eating out, it is also best to be satisfied with one substantial helping of food. Good advice to avoid overeating is to have one serving only, or at least wait a while before taking a second serving, which is often more than is needed. When eating out, prefer 'per kilo' buffet-style self-service restaurants where payment is by weight, and avoid places where unlimited food is offered for a set price. Avoid 'fast food' outlets. These are often noisy. They are organised for quick turnover of customers, and they promote and serve ultra-processed foods, often in super-sizes.

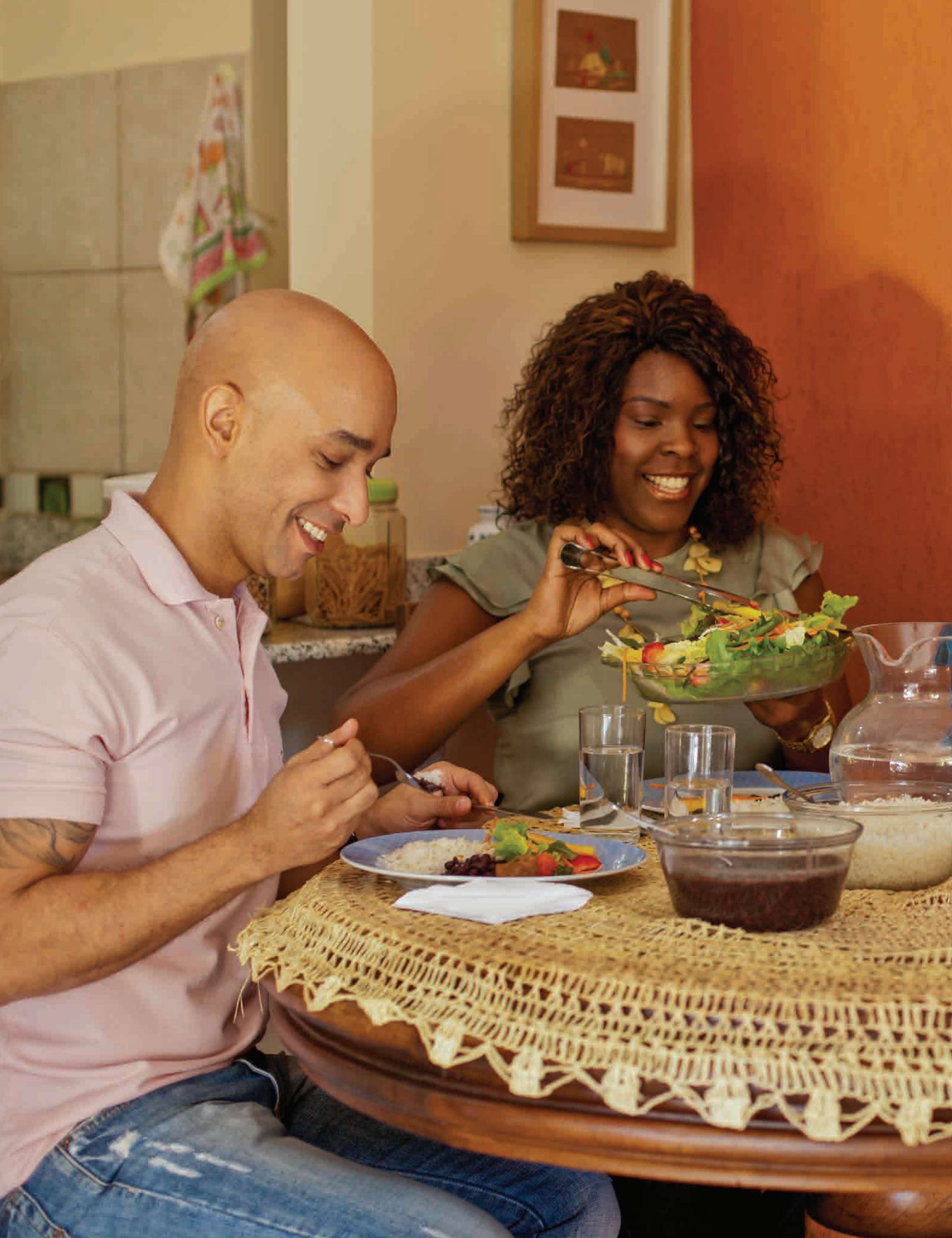

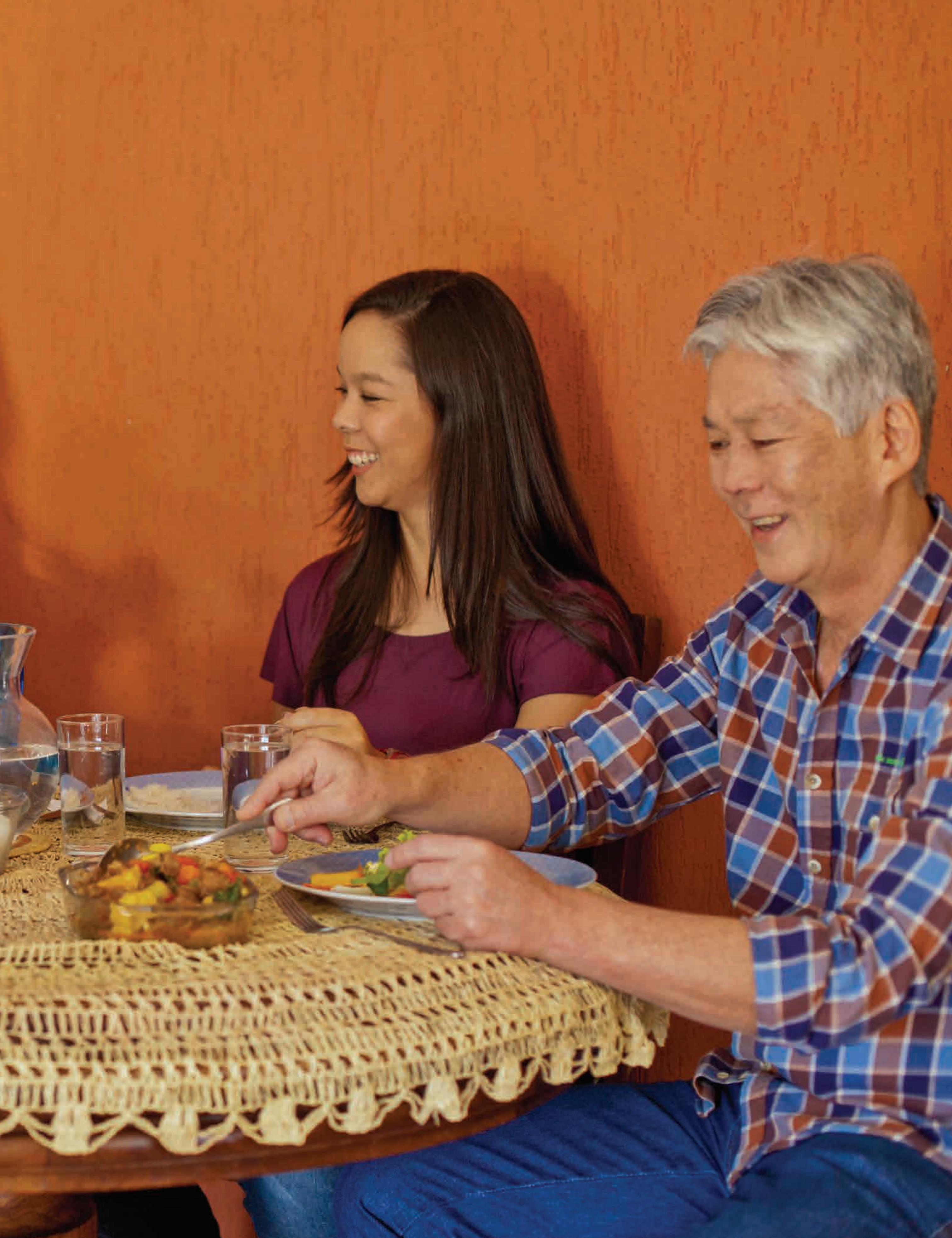

EATING IN COMPANY

Prefer eating together with family, friends, or colleagues. At home, share in acquisition, preparation, cooking and arrangements before and after eating.

Humans are social beings. Eating together is ingrained in human history, as is the sharing and division of responsibility for finding, acquiring, preparing, and cooking food. Eating together, with everything that is involved with eating, is part of the evolution and adaptation of humanity and the development of culture and civilisation. Eating together is a natural, simple yet profound way to create and develop relationships between people. Thus, eating is a natural part of social life.

The sharing of meals at home is a precious and important time for family members and others who may share their lives together, to cultivate and strengthen their ties, to like one another more, to catch up, to exchange views on shared issues, to celebrate successes, to sympathise with difficulties, and to plan for the future. For children and adolescents, they are vital opportunities to acquire good habits and to learn to value the importance of sharing, by means of regular meals in appropriate environments. For adults of all ages, shared meals consolidate co-existence, sympathy, and mutual support.

Eating in company when away from home, including at work or school, helps colleagues and friends to get acquainted and to exchange experiences. It encourages teamwork, enables people to get to know the nature of others, increases the sense of belonging, and enhances the performance of work or school tasks.

Occasions such as weddings, birthdays, and other celebrations marked by special meals, are memorable times for people who like one other and want to be together.

Eating in company usually inhibits hurried eating. It also stimulates people to eat in more pleasant and suitable environments,

because dining tables and place settings are required. Making the arrangements for a shared meal should be enjoyable, like the pleasure of sharing and explaining a favourite recipe.

Thankfully it is still common to share meals in Brazil. Daily meals are usually prepared for the whole family. Friends from work or school prefer to eat in company. Special shared meals on weekends and celebrations are valued because of the coexistence they provide. This tradition is a precious part of national culture, which needs to be protected and strengthened. All the more so, because all over the world, food cultures based on shared meals are being eroded by the increasing manufacture, promotion and consumption of ultra-processed foods.

At home, aside from eating together, it is best of all for all family members to share in some, most or all of the activities involved before, during and after the shared meal itself. These include planning what will be eaten, food acquisition and purchase, the preparation and cooking of meals, clearing up afterwards, discussing what went well and what not so well, learning lessons from experience, and contributing to making the dining room an especially pleasant place.

When all family members work as team members, understanding and sympathy grow. Modern life is marked by increasing demands and the chronic lack of time, which are, nowadays, common to men and women. Therefore, sharing in responsibility is also fair, when otherwise one or only some family members have been taking the whole responsibility. The involvement of children and adolescents in food shopping and preparing meals teaches them about food, and lets them learn about new foods, new ways to prepare foods and where they come from and how they are produced. Learning good habits around table and when eating with the family and sharing responsibilities, all help children and adolescents to grow up well.

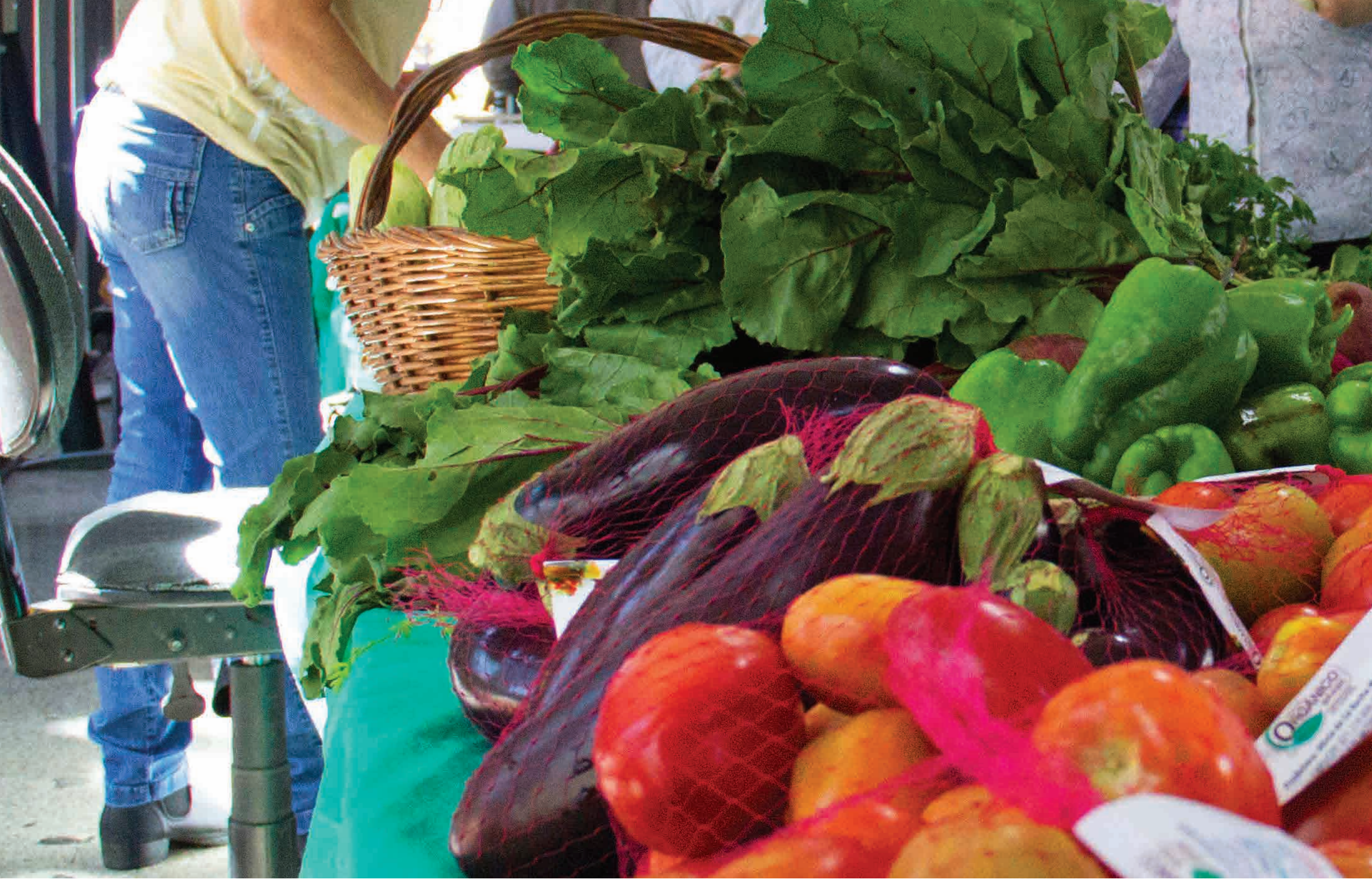

FINALLY

The three recommendations, brought together at the end of this chapter, are easier to follow when practiced together. Regularity and adequate time for meals require an appropriate environment, which is easier and more natural when people eat together. Appropriate environments help increase concentration in the act of eating. Eating in company prevents eating too quickly. These recommendations help you to enjoy the foods you eat and the pleasures provided by the diet.

Obstacles in the way of following the recommendations presented here and in previous chapters are examined, and ways to overcome them discussed, in the next chapter.

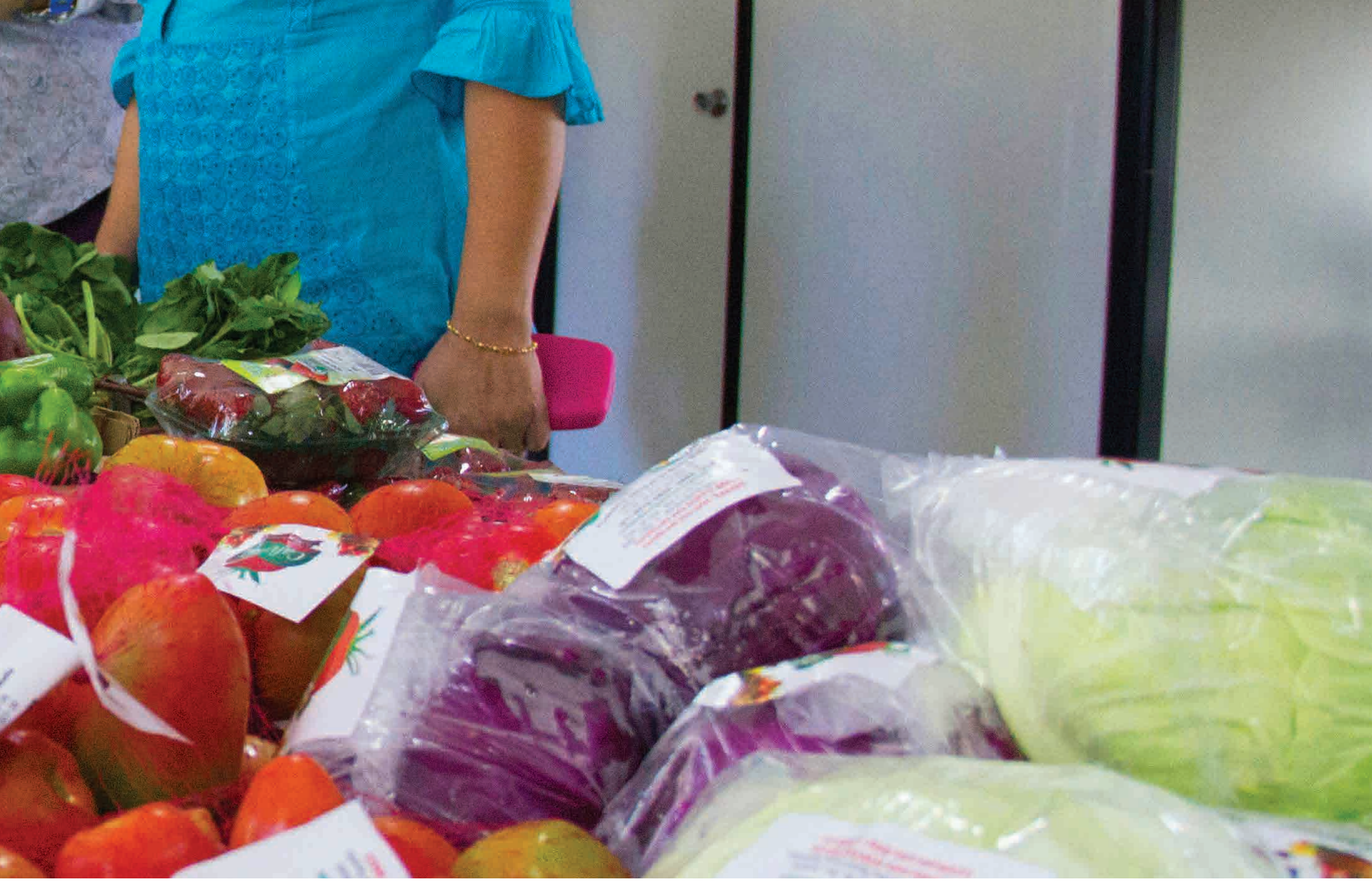

MODES OF EATING

Eating regularly and carefully

Always when possible, eat daily meals at similar times. Avoid 'snacking' between meals. Eat slowly, with full attention, and enjoy eating without engaging in another activity.

Eating in appropriate environments

Always prefer to eat in clean, comfortable, and quiet places, and where there is no stimulus to consume unlimited amounts of food.

Eating in company

Prefer eating with family, friends, or colleagues. At home, share in acquisition, preparation, cooking, and arrangements before and after eating.

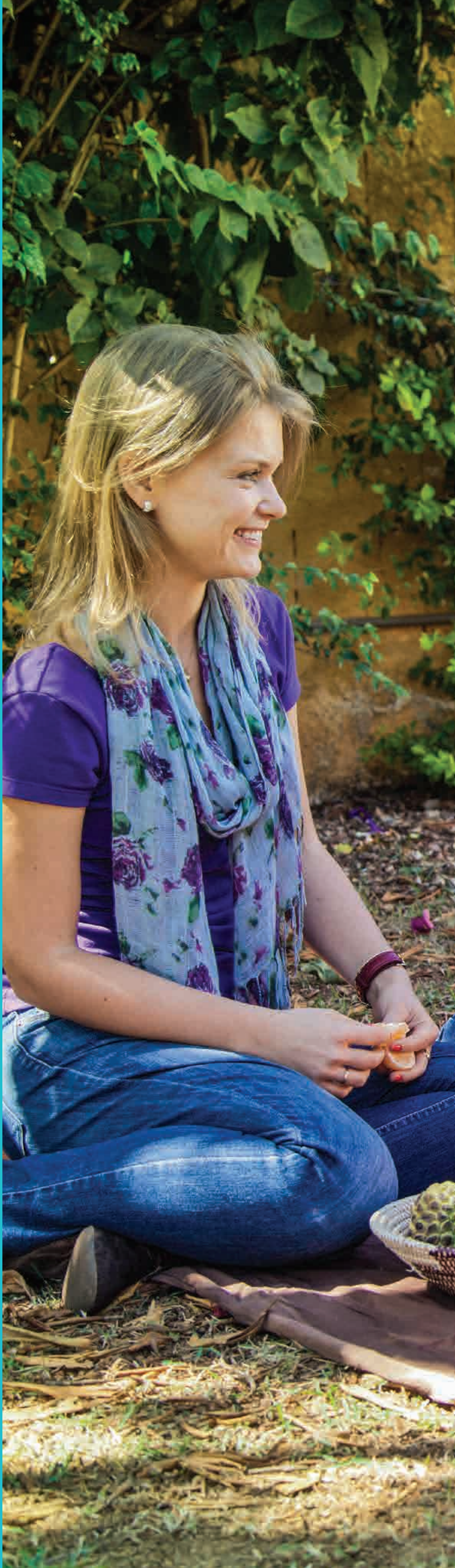

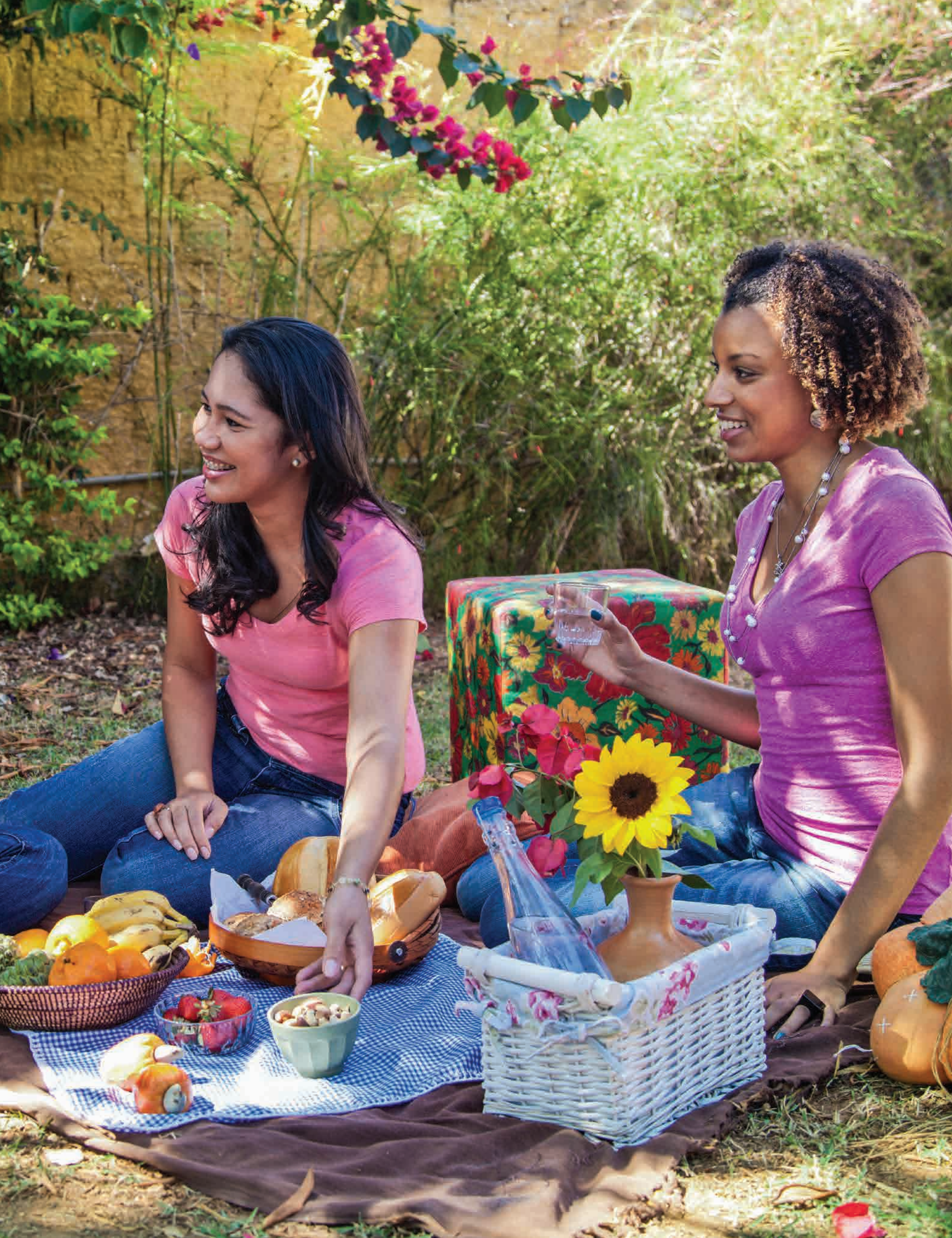

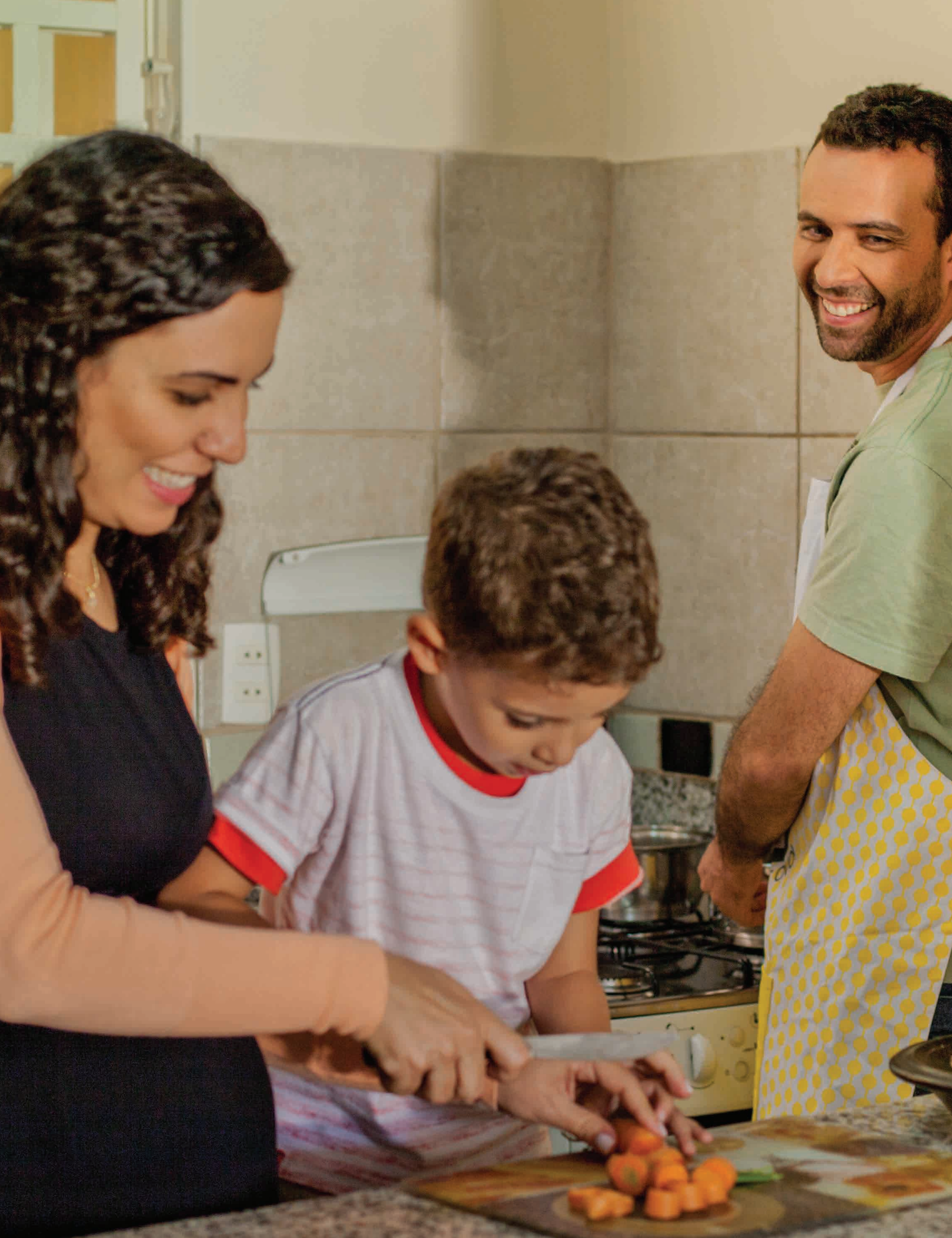

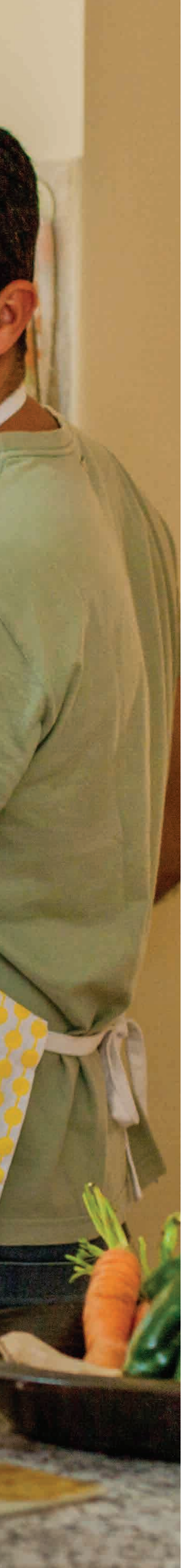

CHAPTER 5. UNDERSTANDING AND OVERCOMING OBSTACLES

Throughout these Guidelines, great importance is given to being realistic and feasible. This rightly also involves being flexible. Thus, instructions, especially of amounts of food, are not used, and instead, terms such as ‘prefer’ and ‘most of the time’ are used. There are practically endless combinations and amounts of foods that make up healthy diets.

A special feature of these Guidelines is that their recommendations are based on the actual diets of a substantial proportion of Brazilian families, whose diets are based on natural or minimally processed foods and dishes and meals made with these foods. So the recommendations are not just theoretical. They are indeed realistic.

Nevertheless, following all the recommendations will not always be easy or straightforward.

This chapter identifies six general obstacles to following the recommendations on the choice of food (chapter 2), the combination of foods in the form of dishes and meals (chapter 3), and modes of eating (chapter 4). These obstacles are of information, supply, cost, skills, time, and advertising.

The chapter continues by giving information, ideas and advice on how these obstacles can be overcome. Some people may some of the obstacles easy to overcome. Other people will have problems with some or even most of the obstacles. Overcoming all of them is likely to involve

considerable commitment, patience and persistence, one reason being that some cannot be overcome personally. Sometimes what is needed is for people to accept and act on the fact that food, diet and nutrition is vital for their own health, and also that of the other people in their lives. This implies finding more time and giving more attention to food and eating. This often implies changes in habits. At other times what is needed, are public policies and regulatory actions that make Brazil nationally, municipally and locally an environment that in effect supports these Guidelines. Here it is of great value that the Brazilian constitution specifies that it is the duty of the State to guarantee the human right to food, and with it, sovereignty and food and nutrition security.

This chapter addresses people personally, and also and vitally, as citizens. This is essential for the adoption of enlightened public policies and effective regulatory actions that will benefit all Brazilians. People can act as citizens themselves, and as members of neighbourhood, community, work and school groups, as users of public services, and also within political parties or civil society organisations. All such commitment and advocacy supports Brazil as a participatory democracy with a civil society committed to securing and strengthening regulations and other public initiatives that protect public health and the common good.

The Brazilian government, working with civil society, has already agreed policies and undertakings of great social importance that have contributed to remove obstacles in the way of the recommendations of these Guidelines. These include increasing the income of the poor, ensuring universal access to basic education and health services, the support and protection of breastfeeding, technical assistance and financial support to family agriculture, the creation of public facilities that offer affordable meals and foods, and healthier meals in schools. But there is a long way to go before all Brazilians can adopt all the recommendations in these Guidelines.

INFORMATION

There is a lot of information on diet and health, but there are few reliable sources

There is more and more information and recommendations about food, diet, nutrition and health on television and radio, in newspapers and magazines, and on the Internet and social media. However, most of their usefulness is questionable. With valuable exceptions, they tend to emphasise specific foods propagated as ‘super foods’ and to ignore the importance of varying and combining foods from different food groups. As such, they induce fads and depreciate traditional and healthy foods and dietary practices. Most advice confuses healthy eating with weight loss dieting regimes. Sometimes informative notices are actually a veiled form of advertising for ultra-processed foods.

What you can do

First, use these Guidelines as a reliable source of information and advice about diet and health and well-being. Their content is based on solid knowledge coming from a variety of scientific studies on food and nutrition, including from studies undertaken in Brazil that are representative of the Brazilian population, and also on the valuable knowledge contained in traditional dietary patterns that have been developed, perfected, and transmitted across generations.

Personally, or as a parent, friend and colleague, you can do more. After reading these Guidelines, discuss the information and recommendations they contain with the people in your life, including the health professionals you consult. If you yourself are a healthcare professional, a community worker, a social worker, an educator or a human resource trainer, please incorporate these Guidelines and the information and recommendations they contain in your own work and the advice you give to all those you work with.

As a citizen, you can do still more. For example, as a parent, at the parents and teachers association at your child's school, you can propose that food and health themes should be given a high priority in the school curriculum so that the pupils learn about food, nutrition and health, and learn where healthy food comes from. As a parent, worker or health professional you can make sure that information from these Guidelines including its Ten Steps to a Healthy Diet is put on notice boards. Be an advocate, on behalf of your family, friends, colleagues and community. In any community group or civil society organisation that you belong to or can join, propose that the information, recommendations and implications of these Guidelines be part of the organisation's work and advocacy, in partnership with other relevant national, municipal and local organisations.

SUPPLY

Ultra-processed foods are on sale everywhere, promoted by advertisements and discounts on all media. By contrast, natural or minimally processed foods get little publicity and some are not even available close to people's homes.

Until recently, most people's diets were made up from food purchased from specialist shops like grocers, greengrocers and butchers, and from municipal, small and street markets and vendors, or from meals at local restaurants and bars. Some was purchased or acquired direct from producers, and people in the countryside produced some of their own food. Only a few items purchased were ultra-processed foods.

Now the environment in which food is sold, bought and consumed is transformed. Literally thousands of branded ultra-processed foods are available in many types of outlet, ranging from hypermarkets, supermarkets, shopping malls, mini-markets, convenience stores, and other places where a great amount of people pass by, such as subway stations and bus stations and food chain outlets, and places where food was once not much sold, such as pharmacies, gas stations, news-stands, cinemas, and schools and hospitals. Street vendors also sell ultra-processed products. In all locations, these products are advertised and promoted with fashionable design and

posters of models, pop stars, athletes, and other celebrities, with discounts, gifts, and all types of promotions.

For natural or minimally processed foods, the environment has also become transformed. Many small food retailers are no longer in business. Natural or minimally processed foods are sold in well-stocked supermarkets that quite often are a distance from where people live and work. Many of the most prominent items in supermarkets in the centre aisles and at the end of aisles and checkouts are ultra-processed products. Ultra-processed products marketed to children are placed at their eye level. Less profitable items like rice and beans, especially when sold in economical bulk, are placed inside or back aisles. Smaller supermarkets and convenience stores often have limited or poor stocks of vegetables and fruits. Supermarkets that are at a distance from home or work demand shopping expeditions by car once or twice a week, which discourages purchases of perishable foods such as vegetables, fruits, and fresh meat and fish.

What you can do

As with the mindful eating recommended in the previous chapter, the first advice is to be mindful when shopping for food and eating food away from home.

For a start, avoid shopping for food in places where only or mostly ultra-processed products are marketed, and avoid eating at fast food outlets. Plan your shopping at supermarkets: make a shopping list, and buy only what is on the list unless there are additional natural and minimally processed foods for sale which are really good choices, like fruits in season.

Support and find bargains at speciality shops, municipal and farmers' markets, street vendors, and other places selling fresh or minimally processed foods, including those produced by organic and agro-ecological methods. In some cities vendors roam the streets in trucks, selling vegetables and fruits which they buy from producers and supply centres. Join collective purchasing groups with neighbours or colleagues to get good prices for fresh foods.

By supporting producers and traders who sell natural or minimally processed foods, including those that are produced organically and agro-ecologically, you contribute to the survival and expansion of this vital sector of the farming economy.

Wherever you live, you can grow some of your own food, in your garden or back yard, on balconies, shelves outside where you live, and in pots inside. Becoming your own gardener and producer of vegetables, fruits or even just of some aromatic herbs, increases the sense of value of food. Discover how delicious food can be grown economically and organically, without the use of chemicals. Share information and ideas with neighbours. Involve children.

Good choices when eating out are the 'per kilo' restaurants found everywhere in Brazil which serve freshly prepared dishes buffet-style, where you make your own choice and the price depends on the weight of the food. Many cities also have public facilities called 'popular restaurants' and 'community kitchens', that serve

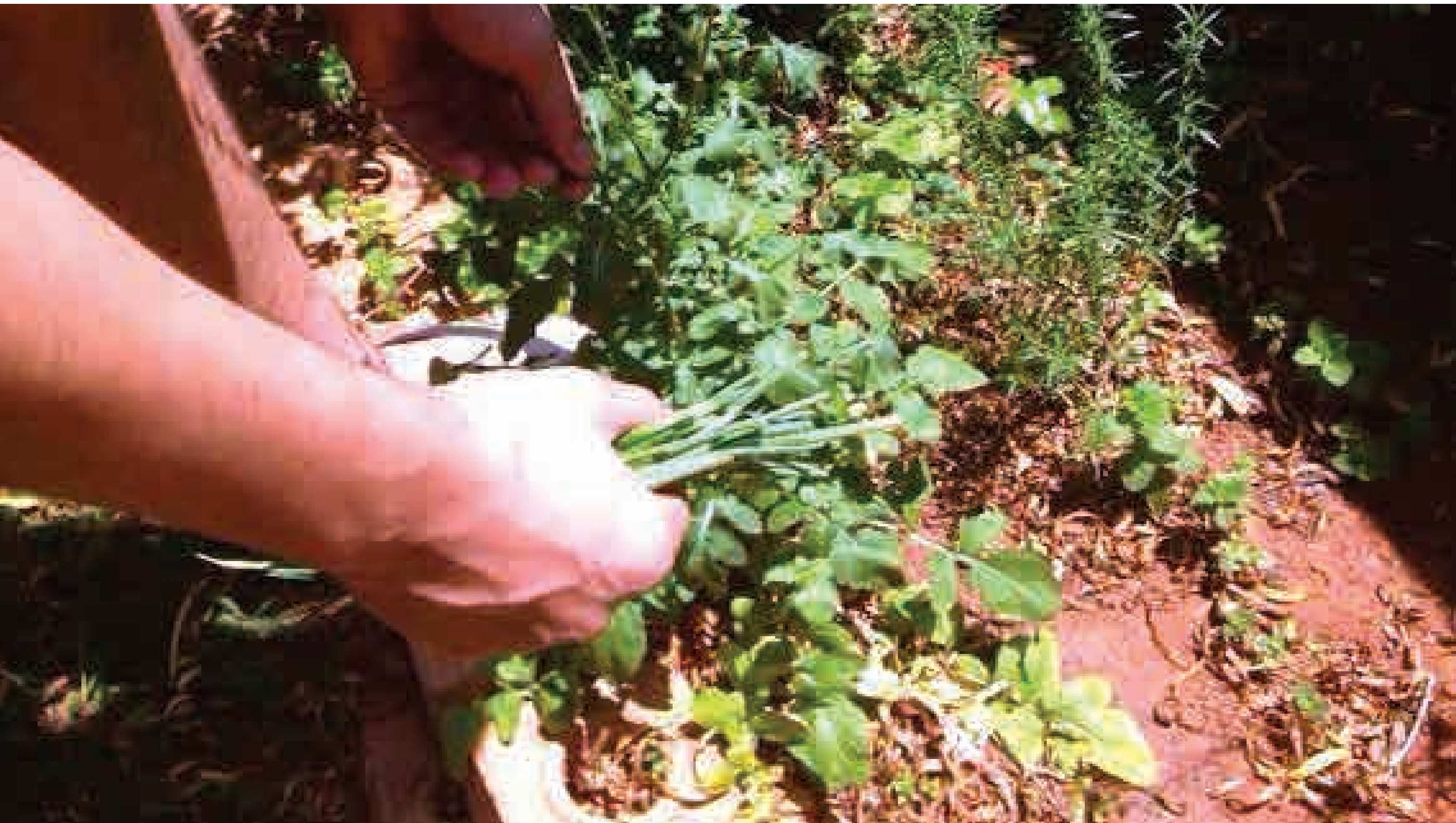

affordable, varied and healthy meals. Another good habit is to take home-made dishes to work or to school.

As a citizen, you can do more. You can encourage the neighbourhood association in your community to get the municipal authorities to create 'popular restaurants' and 'community kitchens'. At work, you and colleagues can press for canteens serving freshly prepared meals and for space in which people can prepare and store fresh food. You can participate in municipal councils that oversee the conduct of the National School Feeding Programme and its integration with family agriculture. As a member of a group or organisation, you can press for natural and minimally processed foods and freshly prepared meals, and not ultra-processed foods, to be available, sold, made and served in schools, hospitals and other institutions.

As a citizen, you can also support the movement to create and develop community gardens to produce organic foods. These gardens, created in city squares, streets and other locations, and within schools, community centres, and health units and other public spaces, encourage interaction, strengthen the community, and produce healthy food. You can go further and as a member of an organisation press the municipal authorities to support urban and peri-urban agriculture projects that encourage organic food production in unused areas in and around cities, including for example planting fruit trees in public spaces.

COST

Although some natural and minimally processed foods are not cheap, the total cost of diets based on natural or minimally processed foods is still lower in Brazil than the cost of diets based on ultra-processed foods.

People often think that it costs a lot to eat healthily and that natural or minimally processed foods are much more expensive than ultra-processed products. Such an impression is reinforced by 'premium' high prices of various ultra-processed products 'fortified' with vitamins and other nutrients or marketed as good for weight loss or which indicate other health claims. However, these products are not healthy.

It is true that perishable foods like some vegetables, fruits, and also fish, have relatively high prices. But these foods are and should be consumed with other natural or minimally processed foods that have lower prices, such as rice, beans, potatoes, cassava, and other staple foods that are part of Brazil's culinary traditions. Also, many vegetables are not expensive, and seasonal vegetables and fruits are best purchased when they are most abundant, have lowest prices, and have most flavour. It is also possible to buy vegetables and fruits in bulk, from producers and wholesale shops.

In general and as a whole, the impression that healthy diets necessarily cost more than unhealthy diets is mistaken. Calculations based on Brazilian household budget surveys show that diets based on fresh and minimally processed foods, and dishes and meals made with these foods and culinary ingredients, are cheaper than diets made of ultra-processed foods, as well as being healthier.

It is true that in other countries, where ultra-processed foods already dominate food supplies, these have become increasingly cheaper than natural or minimally processed foods. This could happen in Brazil, in which case what is now mostly only an apparent obstacle would become real.

What you can do

To save money when buying vegetables and fruits, you should prefer varieties that are in season, because these always have the lowest price. Buying these foods in places where there are fewer intermediaries between the farmer and the final consumer, such as 'sacolões' (public markets specialising in fruits and vegetables), can also reduce costs. Better yet is to buy directly from producers, either at farmers' markets, or through collective purchasing groups. In this way, organic varieties can become quite affordable.

The expansion of the production of natural or minimally processed food, particularly those originating from agro-ecological agriculture, depends on increased demand. With the increased demand for these foods, there will be a corresponding increase in the number of producers and traders, and consequently, price reductions.

Economical options for fresh prepared meals away from home are the per kilo restaurants where customers make their own choice of dishes on offer. At work or school or when travelling, inexpensive options are to bring fresh food and dishes from home.

As a citizen, acting collectively, much can be done. You can press municipal authorities to install public facilities that sell natural or minimally processed foods and freshly made meals at affordable prices. As a member of a community group or civil society organisation you can also advocate and campaign for fiscal and other statutory public policies that protect local farmers and the prices of their produce so that these remain absolutely and relatively cheap, and that make ultra-processed foods relatively expensive.

COOKING SKILLS

Cooking and other culinary skills are no longer being shared between generations. This favours consumption of ultra-processed foods.

Natural or minimally processed foods, when not eaten by themselves, are selected, prepared, seasoned, cooked, combined with other foods and ingredients, and made into dishes and meals. These culinary skills have been devised, developed and perfected in all societies, and adjusted and improved from generation to generation. They make natural or minimally processed foods into attractive, delicious, satisfying and sometimes memorable meals.

In contrast, ultra-processed foods are characteristically made ready-to-consume, or sometimes require nothing more than heating. This is a reason why in Brazil and many other countries, knowledge and practice of culinary skills is often not being passed from older to younger generations. Young people are increasingly not able or willing to prepare meals. The acts of preparing, combining, and cooking food and making meals as

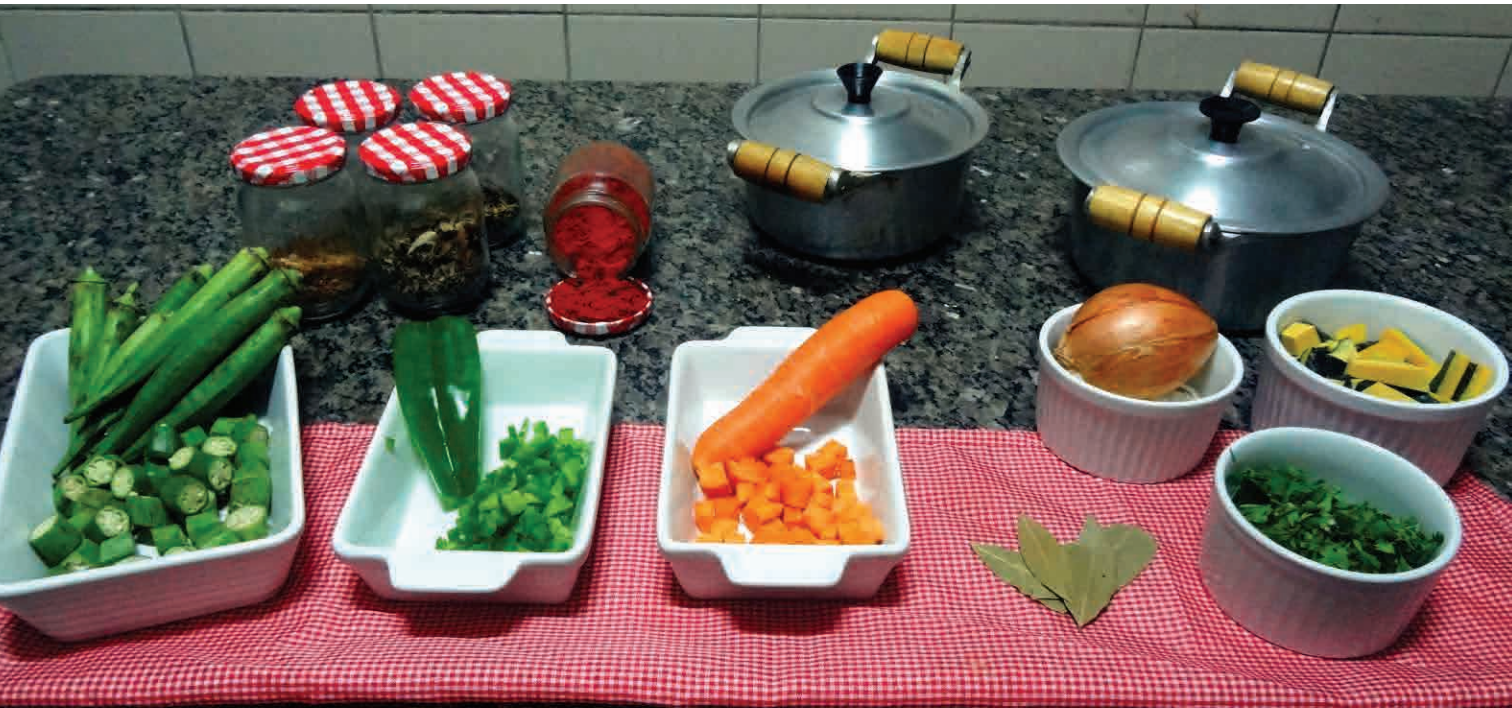

a cultural and social practice are often becoming devalued. Two more reasons are the multiplication of daily tasks, and the incorporation of women into formal employment. Another reason is the constantly increasing availability and incessant advertising of ultra-processed foods. Manufacturers suggest that the processes used to make their products are effectively the same as those used at home, which is not the case. They also suggest that making meals at home is a waste of time better used more productively, which, given the central value of meals personally and within families and communities, as well as their health benefits, is also not factual.

Societies in which few people know how to cook, are for this reason alone bound to be ones in which most foods consumed are ready to eat or drink ultra-processed foods. This process is accelerated by formulation and marketing of these products as 'irresistible'. Food science and technology is constantly developing new products that have hyper-attractive appearance, smell, taste and texture. This is a major obstacle to following the recommendations of these Guidelines

What you can do

If you know how to cook, and to prepare and present meals, develop your knowledge and share it with everybody you live with, especially children and young people – boys and girls.

If you do not have culinary skills, learn them. Spend time assisting family members and friends as they prepare food and cook meals. Go shopping with them, try making some dishes, get recipes from family, friends, and colleagues, read books, check the Internet, and eventually take courses. Start cooking!

Culinary skills, like all other, improve when practiced. You may well be impressed by the progress you make, and other family members will be impressed too. Whenever you can, cook in company with others to make the occasion social. Shared pleasure is redoubled.

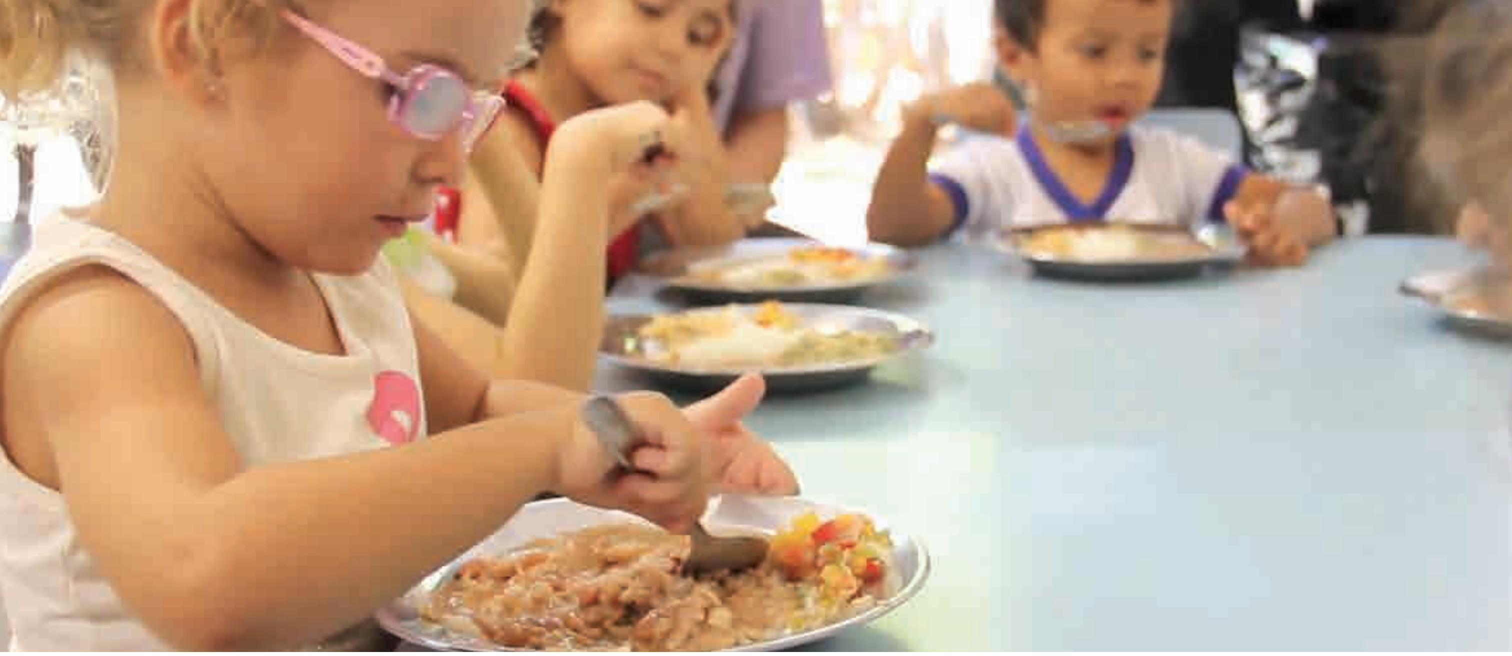

If you are really pressed for time, or live in a place where cooking is difficult, or at first feel inhibited, make clear that you appreciate and value the art of cooking, and encourage people around you to cook, especially young people. If you are involved with health promotion, include cooking themes in professional meetings and presentations and discussions with the public.

There is a great deal also that you can do as a citizen. In parent-teacher meetings, press for cooking and all culinary skills to be included in the formal school curriculum. Valorise the culinary heritage and the gastronomic traditions of Brazil and your region. Engage with civil society organisations dedicated to taking pride in Brazilian traditions, history and heritage.

TIME

The recommendations in these Guidelines are likely to take additional time

Consumption of dishes and meals based on natural or minimally processed foods combined with culinary ingredients, which is the central recommendation of these Guidelines, presupposes the

selection and acquisition of food, its preparation, seasoning, and cooking, the presentation of the meal, and clearing up afterwards. Obviously this all takes time.

These Guidelines also recommend that daily meals be regular, in agreeable surroundings, and that they be consumed mindfully, without doing something else at the same time apart from enjoying being in company. All this takes time too.

Modern life is demanding. More and more people have to work long hours. Most women are now formally employed. Travel to and from work can take a long time and be stressful. So lack of time is liable to be an obstacle to following the recommendations in these Guidelines.

What you can do

Bear in mind that the lack of culinary skills makes the preparation of meals based on natural or minimally processed foods much more time consuming. By improving your cooking techniques, you can greatly reduce the time spent on food preparation. For example, the time to prepare a delicious pasta dish with tomato sauce and natural seasonings is only five minutes longer than the time spent heating water to dissolve one package of 'instant noodles' loaded with fat, salt, and additives. The recommendation to acquire or improve culinary skills and share them with whom you live is also relevant here.

Culinary skills are not just about cooking. They also involve planning, shopping, organisation of kitchen stores, preparing ingredients, and planning what will be eaten in the days ahead.

Some foods, such as beans, that require longer time to prepare, can be cooked in larger amounts on one occasion, frozen, and used throughout the week. Certain dishes such as soups, omelettes, and rice with sautéed vegetables take little time for preparation and may be preferable on specially busy days. Vegetables can be cleaned and dried in advance for use throughout the week.

The time consumed in the preparation of meals based on natural or minimally processed foods is decreased with the development of culinary skills. But time is still needed, and this may be too much for one person, considering all the activities from buying and selection of food to cleaning and cooking. Hence the recommendation above, that family and household members share responsibility for all household activities related to food acquisition and preparation of meals.

More time is also needed to follow the recommendations for the act of eating itself, to eat meals regularly, leisurely, to enjoy the pleasure of the sight, aroma, texture, and taste of food as prepared, and to share this pleasure with family, friends, or colleagues.

Sometimes, as already mentioned, the removal of obstacles in the way of recommendations involves reflecting on the value of food and giving it more importance.

Giving a greater value to food, diet and nutrition makes it easier to follow all the recommendations in these Guidelines, and is especially helpful with the act of eating. Here, guiding thoughts are 'Eating and all that is involved in eating is not a burden, it is a privileged time of pleasure', and 'Assess all the ways that time is used and decide what can make way for food and eating'.

This does not mean though, that everybody can find enough more time just by making personal choices. As with overcoming other obstacles, what works best is a combination of actions personally, as a family member, and as a citizen. Spending less time in the house being entertained, is a family as well as personal decision. Finding ways to cut time taken to and from work, which should include more investment in public transport, is a personal decision which can involve employers, and is a civil action.

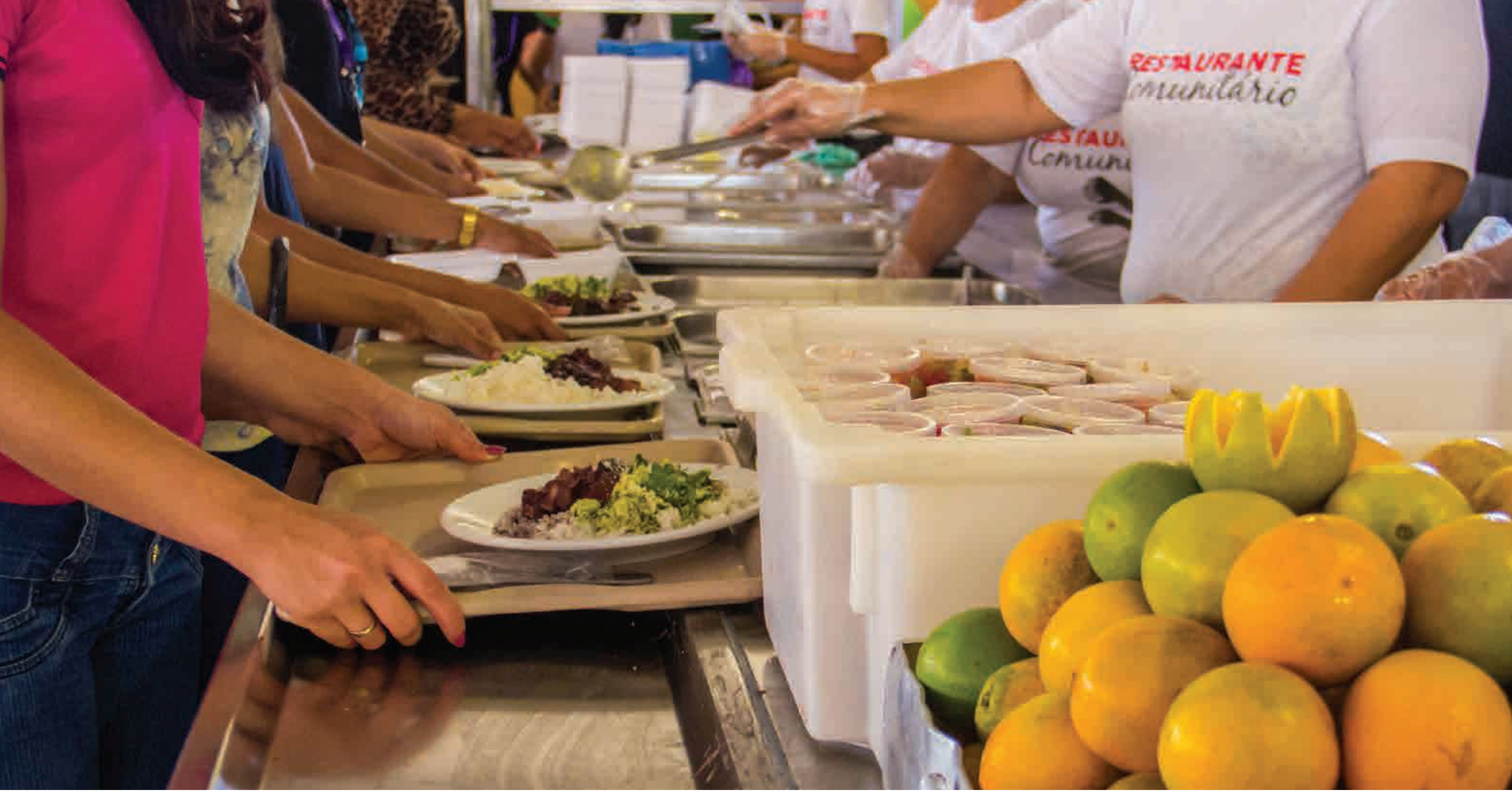

ADVERTISING

The advertising of ultra-processed products dominates commercial advertising of food; it often conveys incorrect or incomplete information about diet and health and mainly affects children and youngsters

Ultra-processed foods are promoted and advertised incessantly, on television and radio, newspapers and magazines, the internet, social media, at point of sale, and on packaging, and with discounts and give-aways. Much of this propaganda is aimed at children and young people.

More than two-thirds of commercials aired on Brazilian television are for food products sold in fast food chains, 'snack packs', cookies, cakes, breakfast cereals, candies and other sweets, soft drinks, sweetened juices, and powdered drinks, which are all ultra-processed products. Most of these commercials are targeted directly at children and adolescents. The stimulus for daily consumption in large quantities is incessant.

On the basis of what they see in commercials, children, adolescents, and the general population are led to believe that ultra-processed products collectively are superior to other foods or that they will make people happier, attractive, strong, 'super-healthy' and socially acceptable, or even that the 'energy' (calories) in them is needed for growing, active life, or sports.

Commercials, advertisements, offers, promotions, and packaging are compelling and seductive for adults. They are even more so for children. Children are in the process of developing, and by themselves, cannot understand many of the elements of the adult world. Increasingly, children are the target audience for ultra-processed product advertising. This is because they influence family purchasing choices and also because they are forming habits that could be lifelong.

Advertising directed at children using what most attracts them, such as heroes, celebrities, music, toys, games, and collectibles, surround them at home, in the street, in shops, at school, in public spaces, parks, and restaurants, and make intensive use of television, the internet and social media, to which they are most attracted.

What you can do

The biggest concern here is advertising focused on children. Parents and educators must explain that the function of advertising is essentially to increase the sale of products, and not to inform or educate people. Limiting the amount of time children spend watching television and using computers is a way to reduce their exposure to advertisements, and at the same time, make them more active. Actions speak louder than words. Adults should also follow the guidance they give to children.

Acting as a citizen, you can do more. You can insist that the school you attend or work at, as well as your children's school remain free of advertisements of any kind. You can also ask

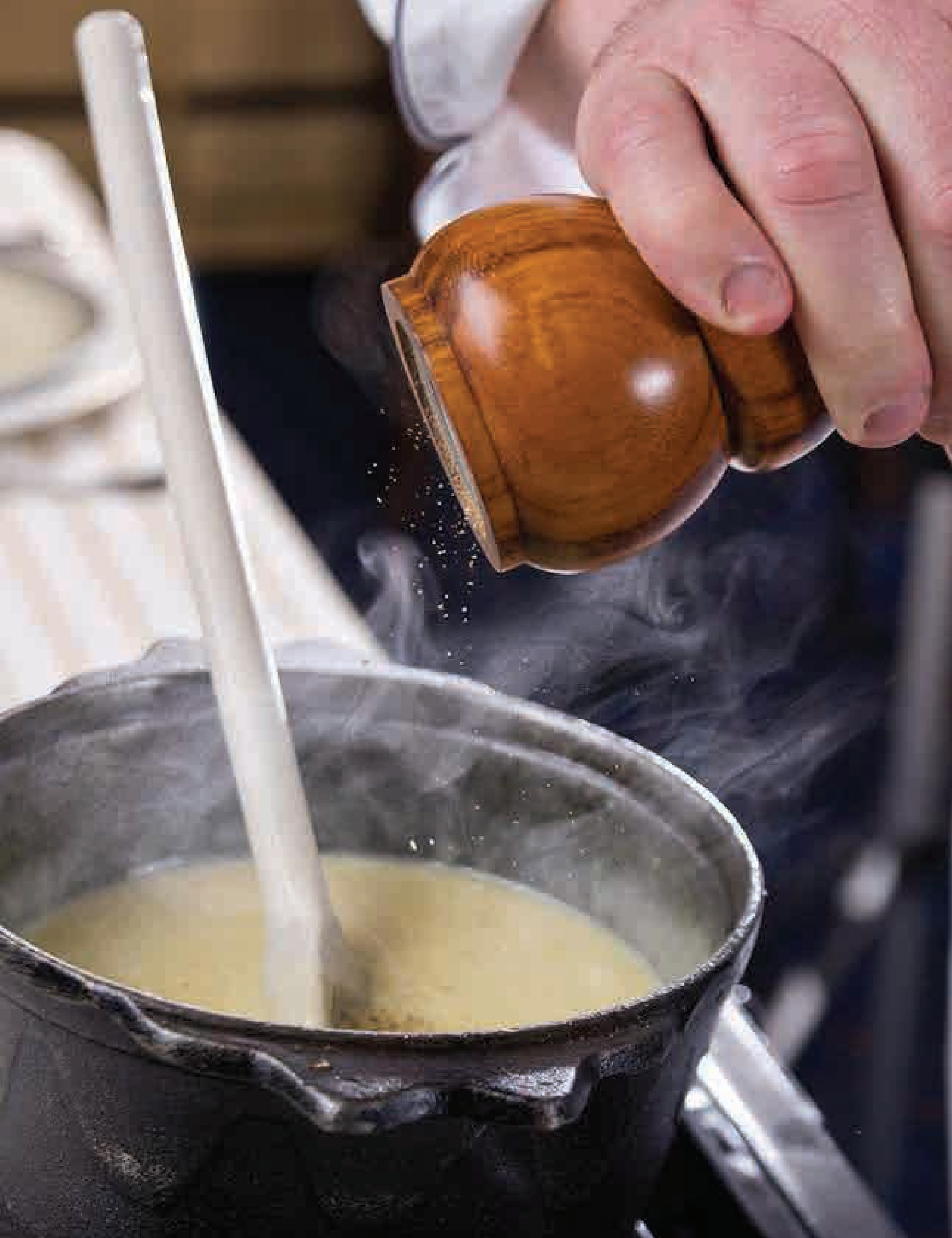

that schools offer discussions about the role of advertising so that students become empowered to understand how these work and to resist them. As a member of civil society, you can propose the engagement of civil society organisations in campaigns for food advertising regulation and legislations to protect children and adolescent rights. As a voter, you can talk to your representative in the Congress about the need for bills that protect the population, especially children and adolescents, from food advertising exposure.

To be effective as a citizen, you should know that Brazilian legislation protects consumers from excesses of advertisements. According to the Consumer Protection Code, it is illegal to mislead through advertisements by either conveying false information, or omitting information about characteristics and properties of products and services. Moreover, all advertisements that take advantage of the deficient judgment and lack of experience of a child are considered abusive. (Regulations that describe characteristics of abusive advertising are included in these Guidelines in the section “Further reading’).Various official public bodies, such as Procon, the Public Prosecutor, the Public Defender’s Office, the Ministry of Justice, and the Ministry of Education, can be pressed and encouraged to take legal action whenever instances of non-compliance with the law are noted.

FINALLY

There certainly are obstacles in the way of following the recommendations of these Guidelines by Brazilian population. This chapter has given six examples: scarcity of reliable information, problems of supply of healthy foods, the relatively high cost of some perishable foods, the loss of culinary skills, lack of time, and incessant advertising and promotion of ultra-processed foods, especially to children and young people.

Overcoming these obstacles is often not easy. In many cases, public policies and regulatory actions are necessary to make

the environment more conducive to the adoption of the recommendations. However, overcoming obstacles often demands that people reassess the importance that food has or may have in their lives. Thus, the recommendations in this chapter are aimed at people as individuals, members of families, and citizens, and as members of communities and civil society organizations that work for the common good.

The obstacles to the adoption of the recommendations in these Guidelines are assembled at the end of this chapter, along with some suggestions to overcome them.

UNDERSTANDING AND OVERCOMING OBSTACLES

INFORMATION

There is a lot of information on diet and health, but there are few reliable sources

Read, use, publicise, and discuss the findings and recommendations of these Guidelines with your family, friends and colleagues, and engage with community groups or civil society organisations that can promote healthy eating. Be an advocate. Make sure that the Guidelines and their Ten Steps to Healthy Eating are promoted in all places to which you have access.

SUPPLY

Ultra-processed foods are on sale everywhere, promoted by advertisements and discounts on all media. By contrast, natural or minimally processed foods get little publicity, and some are not even available in locations close to people's homes

Shop mindfully. Avoid places that sell or serve mainly or only ultra-processed products. In supermarkets take and use a shopping list. Support farmers' markets, municipal markets, specialist retailers, and other places that sell varieties of natural and minimally processed foods, and prefer food produced by ecological methods. When you can, grow some of your own food, even if only herbs. Join groups to buy healthy food in bulk. As a citizen join organisations that press for healthy food production and sale in cities and rural areas.

COST

Although some natural and minimally processed foods are not cheap, the total cost of diets based on natural or minimally processed foods is still lower in Brazil than the cost of diets based on ultra-processed foods.

Be aware that many staple minimally processed foods are cheap. These can be bought in bulk. Prefer vegetables and fruits that are in season

and are locally produced. When eating out, choose self-service per kilo restaurants that charge by weight. Press municipal authorities to install public facilities that sell natural or minimally processed foods at affordable prices. Campaign for policies that support local food farmers and growers and that protect the prices of their produce.

COOKING SKILLS

Cooking and other culinary skills are no longer being shared between generations. This favours consumption of ultra-processed foods.

Develop, practice, and share your culinary skills and value the art of preparing and cooking food. Press for the inclusion of cooking and other culinary skills as part of formal school curriculum. Engage with civil society associations committed to promotion of the Brazilian cultural heritage, gastronomy, and regional and local cuisines.

TIME

The recommendations in these Guidelines are likely to take additional time.

To optimise your time, plan in advance what you need to buy, organize the pantry, set in advance the menu for the week, develop your cooking skills and make all your family members share the responsibility for domestic activities related to food. Work should be shared among the family. Find out what tasks family members most enjoy. Simple everyday meals do not take much time. Appreciate that exchanging time taken for home entertainment like watching television, for time together before and after meals and at table, brings deeper satisfaction and improves family life, especially for children.

ADVERTISING

The advertising of ultra-processed products dominates commercial advertising of food; it often conveys incorrect or incomplete information about diet and health and mainly affects children and youngsters.

Explain to children and young people that the essential purpose of advertising is to increase product sale, and not to inform or educate people. Study the Brazilian legislation, which protects consumer rights, and denounce those who do not follow it.

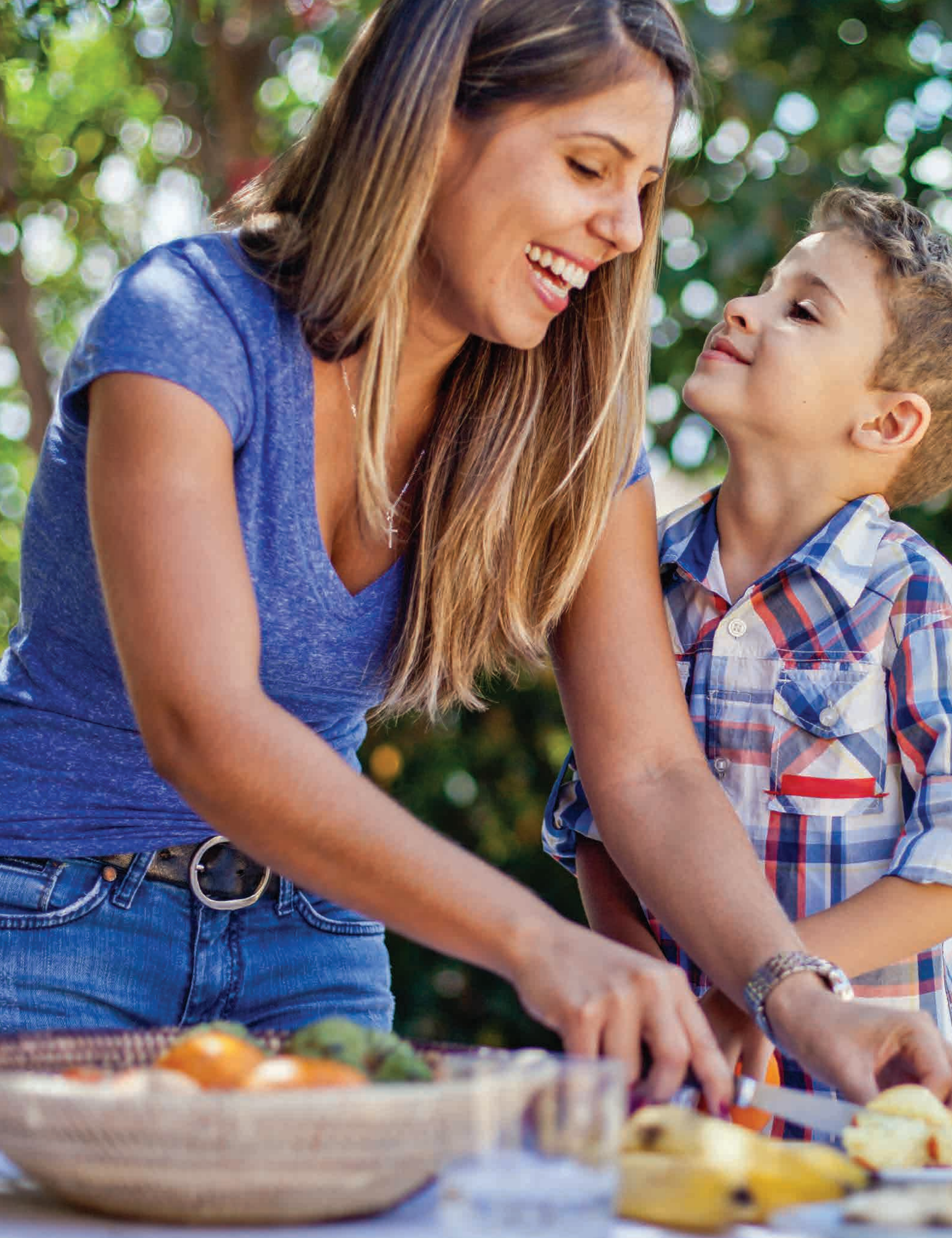

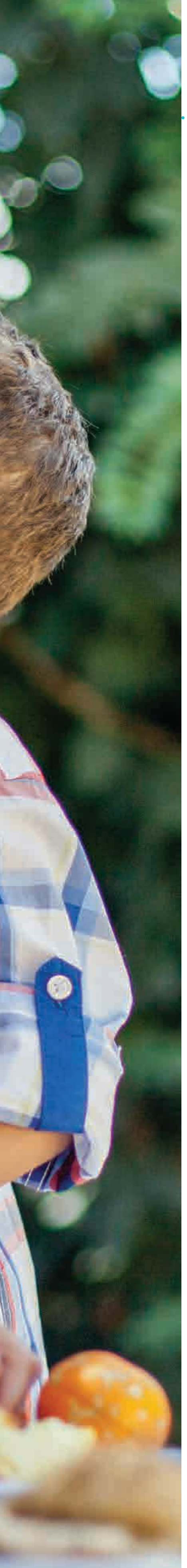

TEN STEPS TO HEALTHY DIETS

1 MAKE NATURAL OR MINIMALLY PROCESSED FOODS THE BASIS OF YOUR DIET

Natural or minimally processed foods, in great variety, and mainly of plant origin, are the basis for diets that are nutritionally balanced, delicious, culturally appropriate, and supportive of socially and environmentally sustainable food systems. Variety means foods of all types — cereals, legumes, roots, tubers, vegetables, fruits, nuts, milk, eggs, meat — and diversity within each type — such as beans and lentils, rice and corn, potato and cassava, tomatoes and squash, orange and banana, chicken and fish.

2 USE OILS, FATS, SALT, AND SUGAR IN SMALL AMOUNTS WHEN SEASONING AND COOKING NATURAL OR MINIMALLY PROCESSED FOODS AND TO CREATE CULINARY PREPARATIONS

As long as they are used in moderation in dishes and meals based on natural or minimally processed foods, oils, fats, salt, and sugar contribute to diverse and delicious diets without making them nutritionally unbalanced

3 LIMIT CONSUMPTION OF PROCESSED FOODS

The ingredients and methods used in the manufacture of processed foods — such as vegetables in brine, fruits in syrup, cheeses and breads — unfavourably alter the nutritional composition of the foods from which they are derived. In small amounts, processed foods can be used as ingredients in dishes and meals based on natural or minimally processed foods.

4 AVOID CONSUMPTION OF ULTRA-PROCESSED FOODS

Because of their ingredients, ultra-processed foods such as salty fatty packaged snacks, soft drinks, sweetened breakfast cereals, and instant noodles, are nutritionally unbalanced. As a result of their formulation and presentation, they tend to be consumed in excess, and displace natural or minimally processed foods. Their means of production, distribution, marketing, and consumption damage culture, social life, and the environment.

5 EAT REGULARLY AND CAREFULLY IN APPROPRIATE ENVIRONMENTS AND, WHENEVER POSSIBLE, IN COMPANY

Make your daily meals at regular times. Avoid snacking between meals. Eat slowly and enjoy what you are eating, without engaging in another activity. Eat in clean, comfortable and quiet places, where there is no pressure to consume unlimited amounts of food. Whenever possible, eat in company, with family, friends, or colleagues: this increases the enjoyment of food and encourages

eating regularly, attentively, and in appropriate environments. Share household activities that precede or succeed the consumption of meals.

6 SHOP IN PLACES THAT OFFER A VARIETY OF NATURAL OR MINIMALLY PROCESSED FOODS

Shop in supermarkets and municipal and farmers markets, or buy directly from producers or other places, that sell varieties of natural or minimally processed foods. Prefer vegetables and fruits that are locally grown in season. Whenever possible, buy organic and agro-ecological based foods, preferably directly from the producers.

7 DEVELOP, EXERCISE AND SHARE COOKING SKILLS

If you have cooking skills, develop them and share them, especially with boys and girls. If you do not have these skills — men as well as women —acquire them. Learn from and talk with people who know how to cook. Ask family, friends, and colleagues for recipes, read books, check the internet, and eventually take courses. Start cooking!

8 PLAN YOUR TIME TO MAKE FOOD AND EATING IMPORTANT IN YOUR LIFE

Plan the food shopping, organise your domestic stores, and decide on meals in advance. Share with family members the

responsibility for all activities related to meals. Make the preparation and eating of meals privileged times of conviviality and pleasure. Assess how you live so as to give proper time for food and eating.

9 OUT OF HOME, PREFER PLACES THAT SERVE FRESHLY MADE MEALS

Eat in places that serve fresh meals at good prices. Self-service restaurants and canteens that serve food buffet-style charged by weight are good choices. Avoid fast food chains

10 BE WARY OF FOOD ADVERTISING AND MARKETING

The purpose of advertising is to increase product sales, and not to inform or educate people. Be critical and teach children to be critical of all forms of food advertising and marketing.

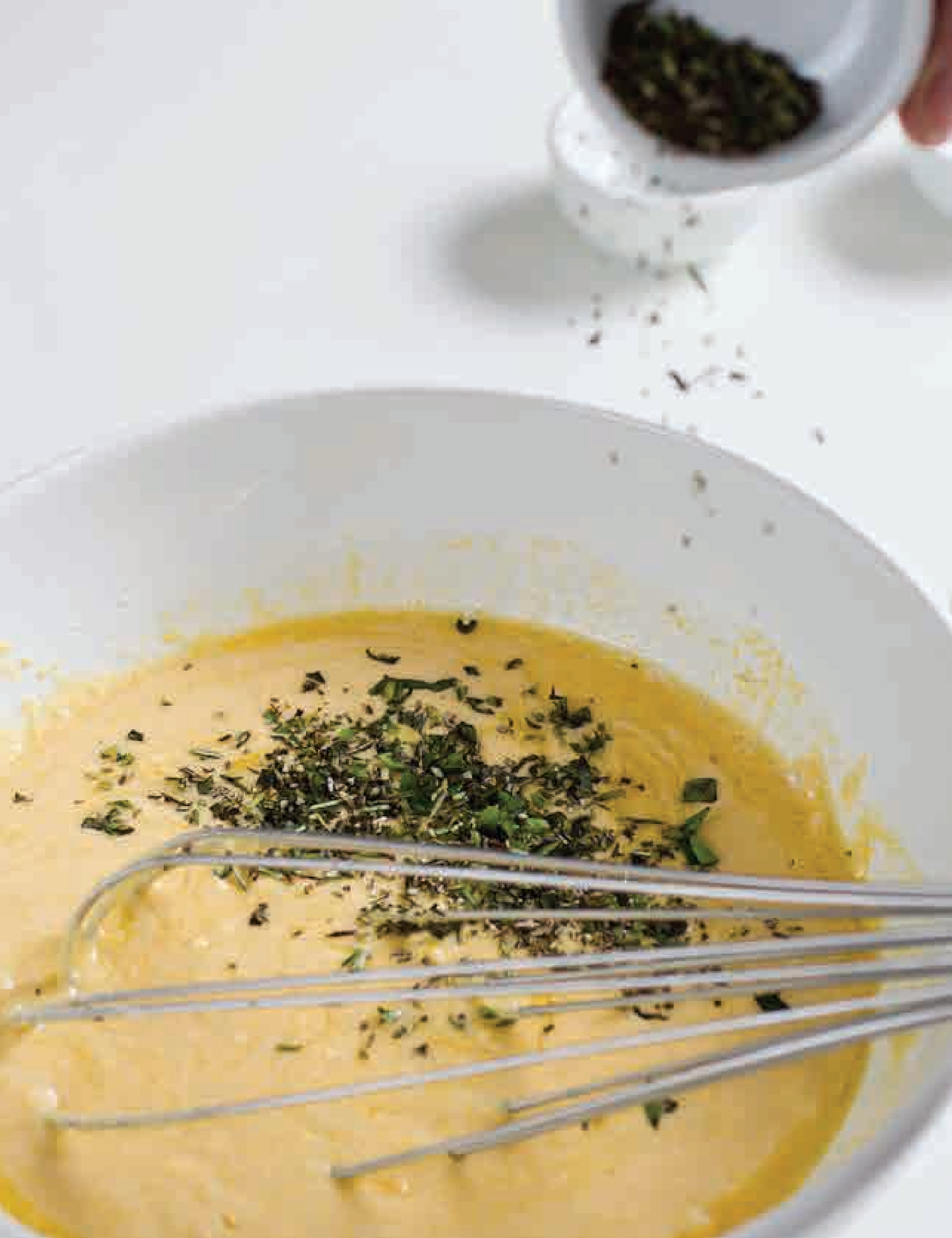

HOW TO KNOW MORE

This section includes suggestions for further reading to develop the contents of these Guidelines, chapter by chapter.

CHAPTER 1 – PRINCIPLES

SCRINIS, G. Nutritionism: the Science and Politics of Dietary Advice. New York: Columbia University Press, 2013.

> *This book develops a thesis in line with the comprehensive approach to the relationship between food, diet, nutrition and health used in these Guidelines, in particular, not identifying foods according to the nutrients contained in them.*

CONTRERAS, J.; GRACIA, M. **Alimentação, Sociedade e Cultura**. Rio de Janeiro: Editora Fiocruz, 2011.

FISCHLER, C. Commensality, society and culture. **Social Science Information**, [S.l.], v. 50, p. 528-548, 2011. Disponível em: <http://ssi.sagepub.com/content/50/3-4/528.full.pdf+html>.

> *The social and cultural dimensions of diets and their influence on people's health and well-being are depicted with great conviction in these two publications.*

LANG, T.; BARLING, D; CARAHER, M. **Food Policy**: Integrating Health, Environment and Society. Oxford: Oxford University Press, 2009.

> *Chapter 6 of this book examines the relationship between production and consumption of foods and the physical environment, with evidence supporting the recommendations in these Guidelines concerning the impact of food choices on ecology and biodiversity. Chapters 7 and 8 examine the relationship between social and cultural dimensions of food and health, and well-being.*

PETRINI, C. **A centralidade do alimento**. Published by the Slow Food movement. Available on: <www.slowfoodbrasil.com/documents/the-central-the-food-carlo-petrini.pdf>.

An integrated approach to the environmental, social, and cultural dimensions of diets and their relationship to health and well-being, by the Slow Food founder Carlo Petrini.

FOOD AND AGRICULTURE ORGANIZATION OF THE UNITED NATIONS. **Sustainable diets and biodiversity**: directions and solutions for policy, research and action. Rome, 2010. Disponível em: <www.fao.org/docrep/016/i3004e/i3004e.pdf>.

Shows, with examples and figures, that the dominant industrial food system and the eating patterns associated with it are not sustainable, despite all the techno-scientific advances of the modern agriculture and food industry. Its important message is: nutritionally balanced eating patterns help to reduce the ecological impact of food production and consumption, and promote biodiversity.

WORLD HEALTH ORGANIZATION; FOOD AND AGRICULTURE ORGANIZATION OF THE UNITED NATIONS. **Preparation and use of food-based dietary guidelines:** Report of a joint FAO/WHO consultation Nicosia, Cyprus. Geneva, 1996. Disponível em: <www.fao.org/docrep/X0243E/X0243E00.htm>.

Chapter 2 of this report outlines the scientific evidence relevant to the construction of food guides, including knowledge generated by health, nutrition, and food researchers, and also by the social, behavioural, and environmental sciences.

COMISSÃO NACIONAL SOBRE DETERMINANTES SOCIAIS DA SAÚDE (Brasil). **As causas sociais das iniquidades em saúde no Brasil.** Rio de Janeiro: Editora Fiocruz, 2008. Disponível em: <http://bvsms.saude.gov.br/bvs/publicacoes/causas_sociais_iniquidades.pdf>.

BRASIL. Ministério do Desenvolvimento Social e Combate à Fome. Secretaria Nacional de Segurança Alimentar e Nutricional. **Marco de referência de educação alimentar e nutricional para as políticas públicas**. Brasília, 2012. Disponível em: <www.fasi.edu.br/files/biblioteca/nut/Marco_referencia_Textocompleto_educaoalimentar.pdf>.

BRASIL. Ministério da Saúde. Secretaria de Atenção à Saúde. Departamento de Atenção Básica. **Política Nacional de Alimentação e Nutrição**. Brasília, 2012. Disponível em: <http://189.28.128.100/nutricao/docs/geral/pnan2011.pdf>.

The first report is an overview of the Brazilian population's health status, emphasising health inequities generated by social determinants. The second report states the need for food and nutrition education, together with public policies that ensure safe and sustainable food supplies in adequate quantity and quality for all. The third is a detailed account of the implementation of these policies in Brazil.

BRASIL. Decreto nº 7.794, de 20 de agosto de 2012. Institui a Política Nacional de Agroecologia e Produção Orgânica. **Diário Oficial [da] República Federativa do Brasil**, Brasília, DF, v. 149, n. 162, 21 ago. 2012. p. 4.

The National Plan on Agroecology and Organic Production (Planapo) is a public policy of the Federal Government that aims to expand and carry out actions to guide the sustainable rural development.

BRASIL. Decreto nº 11.346, de 15 de setembro de 2006. Cria o Sistema Nacional de Segurança Alimentar e Nutricional – Sisan com vistas em assegurar o direito humano à alimentação adequada, e dá outras providências. **Diário Oficial [da] República Federativa do Brasil**, Brasília, DF, v. 143, n. 179, 18 set. 2006. p. 1.

BRASIL. Decreto nº 7.272, de 25 de agosto de 2010. Regulamenta a Lei nº 11.346, de 15 de setembro de 2006, que cria o Sistema Nacional de Segurança Alimentar e Nutricional – Sisan com vistas a assegurar o direito humano à alimentação adequada, institui a Política Nacional de Segurança Alimentar e Nutricional – PNSAN, estabelece os parâmetros para a elaboração do Plano Nacional de Segurança Alimentar e Nutricional, e dá outras providências. **Diário Oficial [da] República Federativa do Brasil**, Brasília, DF, v. 147, n. 164, 26 ago. 2010. p. 6.

The first publication refers to the Organic Law on Food Security and Nutrition which institutionalized the responsibility of the government in promoting the right of all people to regular and permanent access to food, in quality and quantity. The second report is the decree which regulated this law and established the National Policy on Food and Nutrition Security.

CHAPTER 2 – THE CHOICE OF FOOD

MOZZAFFARIAN, D.; LUDWIG, D. Dietary Guidelines in 21st Century: a time for food. **JAMA**, [S.l.], v. 304, p. 681-682, 2010.

WILLETT, W. C.; SKERRET, P. J. **Eat, Drink, and Be Healthy**: The Harvard Medical School Guide to Healthy Eating. New York: Free Press, 2005.

The first publication is a paper by two Harvard University researchers. They describe the limitations of seeing the relationship of food with health solely in terms of the nutritional composition of foods, particularly when the main diet-related conditions and diseases are obesity, diabetes, cardiovascular disease, cancer, and other chronic diseases. They also state that the protective effects of food come from food as a whole and not from specific nutrients in foods, or from supplementation with nutrients. They advocate dietary guidelines that favour natural and or minimally processed foods and discourage consumption of highly processed products. This is the approach

taken in these Guidelines, and also in the book (also from Harvard) that includes guidelines for the US population.

LUDWIG, D. Technology, diet, and the burden of chronic disease. **JAMA**, [S.l.], v. 305, p. 1352-1353, 2011. Disponível em: <http://jama.jamanetwork.com/article.aspx?articleid=896031>.

The author of this paper also advocates food guides that focus on natural or minimally processed foods. He refers to ultra-processed products and outlines the mechanisms linking them with obesity and other chronic diseases.

MOODIE, R. et al. Profits and pandemics: prevention of harmful effects of tobacco, alcohol, and ultra-processed food and drink industries. **The Lancet**, [S.l.], v., 381, n. 9867, p. 670-679, Feb. 2013. Disponível em: <www.thelancet.com/journals/lancet/article/PIIS0140-6736%2812%2962089-3/fulltext>.

A paper by an international group of researchers in this leading medical journal. It connects ultra-processed products, alcoholic drinks, and tobacco, and states that their increase in production and consumption is the main cause of the current global epidemic of chronic diseases.

MOSS, M. **Salt Sugar Fat**: how the Food Giants Hooked Us. New York: Random House, 2013.

This book explains in detail why ultra-processed food products contain excessive salt, sugar, and fats.

MONTEIRO, C. A. et al. Ultra-processed products are becoming dominant in the global food system. **Obesity Reviews**, [S.l.], v. 14, Suppl. 2, p. 21-28, Nov. 2013. Disponível em: <http://onlinelibrary.wiley.com/doi/10.1111/obr.12107/pdf>.

This paper examines the levels and trends in marketing of ultra-processed food products in 79 countries, including Brazil. It shows that these products already dominate the food supplies of high-income countries and that, in middle-income countries such as Brazil, the sales growth is rapid and liable to lead to dominance in the near future.

MARTINS, A. P. B. et al. Participação crescente de produtos ultraprocessados na dieta brasileira (1987-2009). **Revista de Saúde Pública**, São Paulo, v. 47, p. 656-665, 2013.

This paper examines the evolution of household consumption of natural and minimally processed foods, culinary ingredients, and processed and ultra-processed products in Brazil. The results document that the presence of ultra-processed products in the Brazilian diet has been growing in metropolitan areas since the 1980s. In five years, this percentage increased five points (from 20.8% in 2002–2003 to 25.4% in 2008–2009) with a corresponding reduction

in perishable and minimally processed foods and culinary ingredients. The increase in ultra-processed products was common to all social groups.

SCHLOSSER, E. **Fast Food Nation:** what the All-American Meal is Doing to the World. New York: Houghton Mifflin, 2001.

PETRINI, C. **Slow Food**: princípios da nova gastronomia. São Paulo: Senac, 2009.

The first book addresses the ill effects of a food system based on ultra-processed products, including the weakening of food culture, the deterioration of the physical environment, and the depletion of natural resources such as energy and water. The second book advocates integration of the production, preparation, and consumption of food. It sees consumers as "co-producers" who can steer food system towards a more equitable and sustainable future. The consumer-producer identified in the book appreciates the symbolic value of foods and culinary ingredients and is a champion of the sharing of meals, conviviality and commensality.

FOOD AND AGRICULTURE ORGANIZATION OF THE UNITED NATIONS. **World Livestock 2011**: Livestock in food security. Rome, 2011. Disponível em: <www.fao.org/docrep/014/i2373e/i2373e.pdf>.

FOOD AND AGRICULTURE ORGANIZATION OF THE UNITED NATIONS. **Livestock's long shadow**: environmental issues and options. Rome, 2006. Disponível em: <www.fao.org/docrep/010/a0701e/a0701e00.HTM>.

Two reports that analyse the impact of livestock on the environment worldwide, contrasting different production systems. They show the urgency of reducing these impacts, in the context of projections showing greatly increased production and consumption of animal food up to 2050. They recommend only moderate amounts of meat in diets

CARNEIRO, F. F. et al. **Dossiê ABRASCO**: um alerta sobre os impactos dos agrotóxicos na saúde. Rio de Janeiro: ABRASCO, 2012. Parte 1.

This dossier published by the Brazilian Association of Public Health (ABRASCO) registers the concerns with the upward climb of pesticide use in Brazil and the contamination of the environment and people, with severe impacts on public health.

AGÊNCIA NACIONAL DE VIGILÂNCIA SANITÁRIA (Brasil); UNIVERSIDADE DE BRASÍLIA. **Rotulagem nutricional obrigatória**: manual de orientação aos consumidores. Brasília, 2005. Disponível em: <www.anvisa.gov.br/alimentos/rotulos/manual_consumidor.pdf>.

This publication presents the Brazilian legislation on food labelling and discusses how already existing mandatory information on packaged foods labels, as the list of ingredients, may contribute to product choice.

CHAPTER 3 – FROM FOODS TO MEALS

IBGE. **Pesquisa de Orçamentos Familiares 2008–2009**: avaliação nutricional da disponibilidade domiciliar de alimentos no Brasil. Rio de Janeiro: IBGE, 2010. Disponível em: <www.ibge.gov.br/home/estatistica/populacao/condicaodevida/pof/2008_2009_aval_nutricional/pof20082009_avaliacao.pdf>.

This report by the Brazilian Institute of Geography and Statistics describes changes in purchase habits in Brazilian households between 1974 and 2009. It shows that natural or minimally processed foods are being displaced by ultra-processed food products.

IBGE. **Pesquisa de Orçamentos Familiares 2008–2009**: análise do consumo alimentar pessoal no Brasil. Rio de Janeiro: IBGE, 2011. Disponível em: <www.ibge.gov.br/home/estatistica/populacao/condicaodevida/pof/2008_2009_analise_consumo/pofanalise_2008_2009.pdf>.

This second IBGE report shows the results of the first national survey on individual food consumption conducted in Brazil in 2008–2009. Its database was extensively used in the analyses done for these Guidelines that describe the main characteristics of Brazilian diets and, in particular, identify those based on meals freshly made natural or minimally processed foods.

WORLD HEALTH ORGANIZATION. **Diet, nutrition and the prevention of chronic diseases**: report of a Joint WHO/FAO Expert Consultation. Geneva, 2003. (Technical Report, 916) Disponível em: <http://whqlibdoc.who.int/trs/who_trs_916.pdf>.

This UN report gives global recommendations for intake including of fats, various types of fat, carbohydrate, free sugars, protein, sodium, dietary fibre, and also fruits and vegetables. These recommendations guided analysis of the food intake of Brazilians conducted on the basis of the 2008–2009 survey.

WORLD CANCER RESEARCH FUND; AMERICAN INSTITUTE FOR CANCER RESEARCH. **Food, Nutrition, Physical Activity, and the Prevention of Cancer**: a Global Perspective. Washington DC: AICR, 2007. Disponível em: <www.dietandcancerreport.org/cancer_resource_center/downloads/Second_Expert_Report_full.pdf>.

This comprehensive report includes recommendations on energy density and red meat consumption adopted by these Guidelines.

SAWKA, M. N.; CHEUVRONT, S. N.; CARTER, R. Human Water Needs. **Nutrition Reviews,** [S.l.], v. 63, Supplement 1, p. S30-S39, 2005. Disponível em: <www.nap.edu/books/0309091691/html>.

The first publication summarises the findings and recommendations of the 2004 US Institute of Medicine report for the intake of water and electrolytes by healthy people throughout their life cycle, taking physical activity and heat exposure into account. The findings are a basis for the advice in these Guidelines.

AGÊNCIA NACIONAL DE VIGILÂNCIA SANITÁRIA (Brasil). **Guia de alimentos e vigilância sanitária. Brasília**, [200?]. Disponível em: <www.anvisa.gov.br/alimentos/guia_alimentos_vigilancia_sanitaria.pdf>.

AGÊNCIA NACIONAL DE VIGILÂNCIA SANITÁRIA (Brasil). **Orientações aos Consumidores**. Disponível em: <http://anvisa.gov.br/alimentos/consumidor/index.asp>.

Two reports of the Brazilian National Health Surveillance Agency (ANVISA) that provide information and guidance on food safety including on selecting, handling, and storing food.

WORLD HEALTH ORGANIZATION; FOOD AND AGRICULTURE ORGANIZATION OF THE UNITED NATIONS. **Preparation and use of food-based dietary guidelines:** report of a joint FAO/WHO consultation, Nicosia, Cyprus. Geneva, 1996. Disponível em: <www.fao.org/docrep/X0243E/X0243E00.htm>.

This UN report recommends that dietary guidelines should always take into account the social, cultural, economic, and environmental factors that affect food availability and eating patterns. It recommends that food guides should be based on actual current dietary patterns rather than numerical targets related to isolated nutrients, with recommendations on the major population health problems. It also states that a variety of dietary patterns protect health and well-being.

CASCUDO, L. C. **História da alimentação no Brasil**. São Paulo: Editora Global, 2004.

FERNANDES, C. **Viagem gastronômica através do Brasil**. 2. ed. São Paulo: Editora Senac São Paulo; Editora Estúdio Sonia Robatto, 2001.

These two books are histories and celebrations of Brazilian cuisine. Luís da Câmera Cascudo's book, first published in 1967, is an outstanding account

of the history of Brazilian cuisines, their indigenous African and Portuguese origins, and the influence of more recent migrations from Europe and other parts of the world. He gives meticulous details of typical Brazilian dishes with their flavours, colours, and aromas. The book also addresses the social and cultural aspects of the culinary arts, including the symbolism of food, the characteristics of meals, commensality, and table manners. Caloca Fernandes' book is a delicious gastronomic journey through all the regions of Brazil. (Specific books with recipes on day-to-day cooking are included below, in the suggested readings for Chapter 5).

BRASIL. Ministério da Saúde. Secretaria de Atenção à Saúde. Departamento de Atenção Básica. **Alimentos regionais brasileiros**. 2. ed. Brasília, 2014.

An extensive list of many types of Brazilian fruits, vegetables, legumes, tubers, cereals, and herbs from all Brazil's regions, with recipes for dishes and meals. Highlights the rich diversity of Brazilian food.

CHAPTER 4 – MODES OF EATING

GARCIA, R. W. D. Reflexos da globalização na cultura alimentar: considerações sobre as mudanças na alimentação urbana. **Revista de Nutrição**, Campinas, v. 16, n. 4, p. 483-492, 2003.

A paper on current food culture highlighting how industrial food supplies have changed urban dietary patterns, in the context of globalization.

STROEBELE, N.; DE CASTRO, J. M. Effect of ambience on food intake and food choice. **Nutrition**, [S.l.], v. 20, p. 821-838, 2004. Disponível em: <www.sciencedirect.com/science/article/pii/S0899900704001510#>.

WANSINK, B. **Mindless Eating:** why we eat more than we think. New York: Bantam, 2006.

COHEN, D. A; FARLEY, T. A. Eating as an automatic behavior. **Preventing Chronic Disease**, Atlanta, v. 5, p. 1-7, 2008. Disponível em: <www.cdc.gov/pcd/issues/2008/jan/07_0046.htm>.

These three publications provide a solid scientific basis supporting the recommendations in these Guidelines on eating regularly and attentively and how to avoid environments that encourage excessive consumption of dietary energy.

BRILLAT-SAVARIN, J-A. **A fisiologia do gosto**. São Paulo: Companhia das Letras, 1995.

POLLAN, M. **Cozinhar**: uma história natural da transformação. São Paulo: Instrínseca, 2014.

The first book is the classic by Jean Anthelme Brillat-Savarin, first published in French in 1825. The second, published almost 200 years later, is by the celebrated US author Michael Pollan. Both celebrate the essential pleasure in all human actions related to food, the choice of food, and the preparation of dishes, from enjoying food to socialising with family, friends and colleagues.

CHAPTER 5 – UNDERSTANDING AND OVERCOMING OBSTACLES

Understanding obstacles

STUCKLER, D. et al. Manufacturing epidemics: the role of global producers in increased consumption of unhealthy commodities including processed foods, alcohol, and tobacco. **PLoS Med**, [S.l.], v. 9, n. 6, p. e1001235, 2012. Disponível em: <www.plosmedicine.org/article/fetchObject.action?uri=info%3Adoi%2F10.1371%2Fjournal.pmed.1001235&representation=PDF>.

MONTEIRO, C. A.; CANNON, G. The impact of transnational "Big Food" companies on the South: a view from Brazil. **PLoS Med**, [S.l.], v. 9, n. 7, p. e1001252. Disponível em: <www.plosmedicine.org/article/fetchObject.action?uri=info%3Adoi%2F10.1371%2Fjournal.pmed.1001252&representation=PDF>.

The obstacles to the adoption of healthy eating represented by aggressive advertising of ultra-processed food products are described in detail in these two papers, the first from a global perspective, and the second with an emphasis on Brazil.

ORGANIZAÇÃO PAN-AMERICANA DA SAÚDE. **Recomendações da consulta de especialistas da Organização Pan-Americana da Saúde sobre a promoção e a publicidade de alimentos e bebidas não alcoólicas para crianças nas Américas.** Washington, DC, 2012. Disponível em: <www2.paho.org/bra/index.php?option=com_docman&task=cat_view&Itemid=423&gid=997&orderby=dmdate_published&ascdesc=DESC)>.

CAIRNS, G. et al. Systematic reviews of the evidence on the nature, extent and effects of food marketing to children: a retrospective summary. **Appetite**, [S.l.], v. 62, p. 209-215, 2013.

These two publications are about food advertising and marketing aimed at children. The first describes the vulnerability of children to advertising,

highlighting the predominance of ultra-processed food products in advertisements and the systematic use of all media. It recommends that countries in the Americas should enact policies that reduce exposure of children to the promotion of unhealthy food products. The second publication, a review article, confirms the predominance of ultra-processed food products in advertising aimed at children, highlighting its harmful effects on food preferences, purchasing habits, consumption patterns, and various health disorders related to food.

MOUBARAC, J-C. et al. International differences in cost and consumption of ready-to-consume food and drink products: United Kingdom and Brazil, 2008-2009. **Global Public Health**, [S.l.], v. 8, p. 845-856, 2013.

This paper is about the influence of prices on the purchasing of foods in Brazil and in the UK. It shows that, in the UK, preparing meals at home costs more than purchasing ultra-processed ready-made food products, whereas in Brazil, home-prepared food costs less. The relatively lower prices of ultra-processed food products in the UK is an explanation of why these products dominate the national UK diet, but as yet not that of Brazil.

SHAPIRO, L. **Something from the oven**. New York: Penguin, 2004.

This book covers US post-war history. It challenges the common notion that the displacement of foods and culinary preparations used in home cooking of meals by ready-to-consume ultra-processed food products was because of the increased paid employment of women out of the home, who have no time to cook. Laura Shapiro shows that the change was largely driven on industry marketing strategies which convinced people that ready-made products meant that there was no longer any need to cook and that the time could be used for more interesting activities. The relationships between the value of food, the time and care needed to prepare meals, and the many benefits that centre on cooking, are also addressed by Michael Pollan in his book Cooked, listed above.

Overcoming obstacles

Publication of the General Coordination on Food and Nutrition, Ministry of Health.

Available in:<http://dab.saude.gov.br/>.

The link is to all relevant publications of the Brazilian Ministry of Health. These include information and guidance on breastfeeding, the nutrition of children until 2 years of age, healthy eating for older people, and regional foods.

BRASIL. Ministério do Desenvolvimento Social. Segurança Alimentar. **Rede equipamentos**. Disponível em: <www.mds.gov.br/segurancaalimentar/equipamentos>.

This link is to information on public polices to reduce national rates of food insecurity and to promote access to adequate and These include information and guidance on popular restaurants, community kitchens, support of family farming, food banks, and popular markets.

SANTOS, M. V. et al. Os restaurantes por peso no contexto de alimentação saudável fora de casa. **Revista de Nutrição**, Campinas, v. 24, p. 641-649, 2011. Disponível em: www.scielo.br/pdf/rn/v24n4/v24n4a12.pdf52732011000400012>.

This paper describes Brazil's system of 'per kilo' restaurants, which serve freshly made dishes buffet-style, charged by weight at fair prices.

HARTMANN, C.; DOHLE, S.; SIEGRIST, M. Importance of cooking skills for balanced food choices. **Appetite,** [S.l.], v. 65, p. 125-131, 2013.

CASTRO, I. R. R. et al. A culinária na promoção da alimentação saudável: delineamento e experimentação de método educativo dirigido a adolescentes e a profissionais das redes de saúde e de educação. **Revista de Nutrição**, Campinas, v. 20; p. 571-588, 2007. Disponível em: <www.scielo.br/pdf/rn/v20n6/a01v20n6.pdf>.

These papers are on the relationship between culinary skills and eating healthily. The first paper contains evidence that ability to cook leads to eating more vegetables including greens, and less ultra-processed food products. The second describes the benefits of education in the range of culinary skills on promotion of healthy eating.

BRASIL. Resolução nº 163, de 13 de março de 2014. Dispõe sobre a abusividade do direcionamento de publicidade e de comunicação mercadológica à criança e ao adolescente. **Diário Oficial [da República Federativa do Brasil]**, Brasília, DF, 4 abr. 2014. Seção I, p. 4.

This 2014 Decree from the Brazilian National Council for Children and Adolescents identifies targeting all advertisements including for food products, to children and adolescents, as abusive. It characterises advertisements aimed at children and lists the places where this practice is considered illegal under the Consumer Protection Code.

POLLAN, M. **Regras da comida:** um manual da sabedoria alimentar. São Paulo: Editora Intrínseca, 2010.

WATERS, A. **A arte da comida simples**: lições e receitas de uma deliciosa revolução. Rio de Janeiro: Editora Agir, 2011.

LOBO, R. **Panelinha**: receitas que funcionam. 5. ed. São Paulo: Editora Senac, 2012.

These three books, by Michael Pollan, Alice Waters and Rita Lobo, a small selection from many others, include practical suggestions that will assist readers to incorporate the recommendation and advice of these Guidelines in their daily lives.

ANNEX A
PROCESS OF ELABORATION OF THE NEW EDITION OF THE "DIETARY GUIDELINES FOR THE BRAZILIAN POPULATION"

This new edition has been developed by the Ministry of Health (MS) in partnership with the Center for Epidemiological Research in Nutrition and Health of the University of São Paulo (NUPENS/USP) with the support of the Brazilian Pan American Health Organization Office (PAHO/Brazil). In order to promote the collective construction and to promote broad participation and discussion of the contents of this document, the General Coordination of Food and Nutrition (CGAN) has organized this elaboration process in six major steps that contributed to the final version of new edition of the Dietary Guidelines for the Brazilian Population:

STEP 1 – LISTENING WORKSHOP

The first step of the process included a workshop, held in November 2011, at the School of Public Health of the University of São Paulo, with participants from all over Brazil. The participants included professionals from health, education, social welfare and agriculture sectors, university professors, leaders of professional councils and professional associations and members of public policy social control councils and consumer protection organizations.

Working groups discussed the following questions: what should a guide or a reference material contain in order to effectively contribute to better food choices by the population? Have you ever used the 2006 Dietary Guidelines for the Brazilian population? How? Do you consider the language and the proposed structure appropriate? The group discussions were reported in a final plenary bringing up the different views and observations resulting from the group debates. The results of this workshop guided the elaboration of the first draft of the new Guidelines

STEP 2 – FORMULATION OF THE FIRST DRAFT

The first draft of the new Guidelines was elaborated by a team of representatives of CGAN, PAHO and NUPENS, between November 2011 and July 2013.

STEP 3 – EVALUATION WORKSHOP

The draft was evaluated in a second workshop, held in August 2013, at the School of Public Health of the University of São Paulo, with the participation of health managers, health professionals, civil society representatives and researchers.

The group was divided in four working groups to answer the following questions about each chapter of the draft:

Did the chapter omit any important content? Is there any irrelevant content or any content that should be removed? Should something be said differently? What would you highlight as strengths and weaknesses of this chapter? What is your overall assessment of the new version of the Dietary Guidelines for the Brazilian population?

The group discussions were reported and discussed in a final plenary. The results of this workshop guided the development of a second version of the document.

STEP 4 – FORMULATION OF THE SECOND DRAFT

The second draft was elaborated, between September and December 2013, by the same team that wrote the first draft. This new draft, after evaluation and approval by the Ministry of Health, was submitted to a public consultation that started on February 10th, 2014.

STEP 5 – PUBLIC CONSULTATION

The second version of the food guide was published in an official public consultation website platform of the Ministry of Health and became available to receive contributions from February 10th to May 7th, 2014.

During this period, several meetings were held throughout Brazil, aiming to foster the discussion of the content of the food guide draft, to welcome different perceptions, to stimulate the disclosure of the consultation and to encourage contributions and suggestions through the public consultation platform.

Among these meetings:

- State workshops promoted by the Ministry of Health with the support of State Health Secretariats (SES) Coordination/Technical references of Food and Nutrition, which were responsible for organizing the event and inviting the participants. 27 workshops (in 26 states and in the Federal District) were performed with the participation of about 30 people per workshop, including

healthcare professionals of SUS, educational and social assistance professionals and representatives of civil society organizations. During the workshops, the participants were asked to previously read the document and the meeting was divided into three phases: 1º) presentation of the second draft; 2º) group discussion, chapter by chapter; 3º) presentation of the summary discussions of each group and plenary discussion. The final product was a consolidated report about all issues discussed, which subsequently was inserted at the public consultation platform by the State Food and Nutrition Coordinators/References, in order to formalize the workshop considerations.

- Meetings with regional nutritionist professional councils and local universities: the regional and state councils organised meetings and invited nutritionists and local public and private universities representatives for discussing the second draft of the food guide.

- Other meetings: the Ministry of Health also discussed the second draft in other spaces and with other social actors such as: the Food and Nutrition for Public Health Taskforce from the Brazilian Public Health Association (GT ANSC/ABRASCO), the National Health Council, the Directive Board of the National Food and Nutritional Security Council (Consea), Federal and Regional Nutritionist Professional Councils (CFN/CRN) and RedeNutri (social network for food and nutrition policy professionals) through online discussions.

- During the three months of consultation, 3,125 contributions from 436 individuals/institutions had been received as described below:

PROFILE OF USERS	NUMBER OF USERS	NUMBER OF CONTRIBUTIONS
Educational Institutions	201	278
Individuals	102	1227
Secretariats, departments and coordinators at federal, state and municipality level	58	350
Food and Nutrition and Food and Nutrition Security councils and organizations and nonprofit institutions.	53	1027
Industry, associations and food syndicates.	17	230
Others	5	13
**Total**	**436**	**3125**

The fifth step was concluded on May 2014 by CGAN/MS, through de compilation of all contributions from the public consultation. Based on this compilation, the elaboration of the final draft was started.

STEP 6 – FINAL VERSION OF THE NEW DIETARY GUIDELINES

The final version of the new edition of the Guidelines was developed, based on compiled contributions of public consultation from June to July 2014 by the technicians and researchers from CGAN/MS, PAHO and NUPENS/USP.

The participatory work adopted throughout the preparation of this new version the Guidelines was a key element to receive the suggestions of a number of people and institutions, and to address various actors and sectors of society interested in health promotion and the promotion of adequate and healthy eating for the Brazilian population.

Considering the diversity of Brazilian regional realities and different population groups to whom this guide is intended, the Ministry of Health will also develop other communication strategies to disseminate its contents, including the preparation of other materials such as manuals, brochures and videos.

PARTICIPANTS

LISTENING WORKSHOP

Aline Cristino Figueiredo – Brazilian Health Surveillance Agency (ANVISA)

Ana Carolina Feldenheimer da Silva – Ministry of Health (Brazil)

Ana Claudia Marquim Firmo de Araújo - Brazilian Health Surveillance Agency (ANVISA)

Ana Maria Cervato-Mancuso – University of São Paulo (USP)

Ana Maria Dianezi Gambardella – University of São Paulo (USP)

Ana Paula Bortoletto Martins - Center for Epidemiological Research in Nutrition and Health, University of São Paulo (NUPENS/USP)

Anayde Lima - Slow Food São Paulo

Anelise Rizzolo de Oliveira Pinheiro – University of Brasília (UNB)

Antônio Fagundes – Ministry of Health (Brazil)

Beatriz Aparecida Edméa Tenuta Martins - Federal Board of Nutritionist (CFN)

Carla Susana Rodrigues - Ministry of Agriculture, Livestock and Food Supply (MAPA, Brazil)

Carlos Augusto Monteiro - Center for Epidemiological Research in Nutrition and Health, University of São Paulo (NUPENS/USP)

Carmem Cemires Cavalcante Costa - State Secretariat of Health of Ceará (SES/CE)

Carolina Belomo de Souza – Ministry of Health (Brazil)

César Nunes Nascimento - Integral Health Institute (ISI/DF, Brazil)

Clara Freire de Araújo - Ministry of Health (Brazil)

Daniela Silva Canella - Center for Epidemiological Research in Nutrition and Health, University of São Paulo (NUPENS/USP)

Deurides Ribeiro Navega Cruz – Ministry of Health (Brazil)

Diogo Thimoteo da Cunha - Federal University of São Paulo (UNIFESP)

Elisabetta Gioconda Iole Giovanna Recine – University of Brasilia (UNB)

Elke Stedefeldt – Federal University of São Paulo (UNIFESP)

Fabio da Silva Gomes - National Cancer Institute of Brazil (INCA Brazil)

Geisa Firmino Torres de Medeiros – PETROBRAS (Brazil)

Geoffrey John Cannon - World Cancer Research Fund (RJ, Brazil)

Janaína Calu Costa - Center for Epidemiological Research in Nutrition and Health, University of São Paulo (NUPENS/USP)

Janine Giuberti Coutinho - Pan American Health Organization (PAHO, Brazil)

Jean-Claude Moubarac - Center for Epidemiological Research in Nutrition and Health, University of São Paulo (NUPENS/USP)

Larissa Galastri Baraldi - Center for Epidemiological Research in Nutrition and Health, University of São Paulo (NUPENS/USP)

Lorena Gonçalves Chaves – Ministry of Education (Brazil)

Luana Caroline dos Santos – Federal University of Minas Gerais (UFMG)

Luciana Ferreira Campos Vasconcelos – PETROBRAS (Brazil)

Luísa Maria Oliveira Pinto - State Secretariat of Health of Ceará (SES/CE)

Luiza Lima Torquato – University of Brasília (UNB)

Macarena Urrestarazu Devincenzi – Federal University of São Paulo (UNIFESP)

Maluh Barciotte - Center for Epidemiological Research in Nutrition and Health, University of São Paulo (NUPENS/USP)

Manuela de Sá Pereira Colaço Dias - Consumer Protection Association (PROTESTE, Brazil)

Marcia Samia Pinheiro Fidelix – Brazilian Association of Nutrition (ASBRAN)

Maria Antonieta de Barros Leite Carvalhaes - Paulista State University (UNESP)

Maria Fabiana Ferro Guerra - Social Service of Commerce (SESC/SP, Brazil)

Mariana Carvalho Pinheiro – Ministry of Health (Brazil)

Mariana de Araujo Ferraz - Brazilian Institute for Consumer Protection (IDEC, Brazil)

Mariana Helcias Côrtes Gonzaga Sagastume - Ministry of Social Development and Fight against Hunger (MDS, Brazil)

Mariana Martins Pereira - State Secretariat of Health of Distrito Federal (SES-DF)

Marina Vianna Ferreira – University of São Carlos (UFSCAR)

Milene Gonçalves Massaro Raimundo - Secretary of Agriculture and Food Supply for the State of São Paulo (SAA-SP, Brazil)

Nildes de Oliveira Andrade - National Health Council (CNS, Brazil)

Péricles Macedo Fernandes - Ministry of Agriculture, Livestock and Food Supply (MAPA, Brazil)

Rafael Moreira Claro - Center for Epidemiological Research in Nutrition and Health, University of São Paulo (NUPENS/USP)

Regina Fonseca - The eight Millennium Development Goals (São Paulo, Brazil)

Renata Bertazzi Levy - Center for Epidemiological Research in Nutrition and Health, University of São Paulo (NUPENS/USP)

Silvia Vignola - Brazilian Institute for Consumer Protection (IDEC, Brazil)

Suely Feldman Bassi - Municipal Secretariat of Health of São Paulo (SMS-SP)

Susana Moreira Padrão – State University of Rio de Janeiro (UERJ)

Tatiane Nunes Pereira – University of São Paulo (USP)

Teresa Cristina Guimarães Magalhães - State Secretariat of Health of Bahia (SES/BA)

Thalita Antony de Souza Lima - Brazilian Health Surveillance Agency (ANVISA)

Theresa Cristina de Albuquerque Siqueira – Ministry of Health (Brazil)

Vanessa Fernandes Davies - Brazilian Social Services for Industry (SESI/SC)

Viviane Laudelino Vieira – University of de São Paulo (USP)

WORKSHOP PARTICIPANTS ASSESSMENT OF THE PROPOSED NEW GUIDELINES

Adriana Bouças Ribeiro – State Secretariat of Health of São Paulo (SES/SP)

Aline Cristine Souza Lopes - Federal University of Minas Gerais (UFMG)

Aline Cristino Figueiredo – Brazilian Health Surveillance Agency (ANVISA)

Amélia Augusta de Lima Friche - Federal University of Minas Gerais (UFMG)

Ana Maria Cervato Mancuso – University of São Paulo (USP)

Ana Maria Dianezi Gambardella – University of São Paulo (USP)

Elizabeth Maria Bismarck-NasrIII – Municipal Secretariat of Health of São José dos Campos (SMS/SP)

Christiane Gasparini Araujo Costa – Institute of Studies, Formation and Assistance in Social Policies (POLIS/SP)

Elaine Leandro Machado – Ministry of Health (Brazil)

Elisabetta Gioconda Iole Giovanna Recine – University of Brasília (UNB)

Estelamaris Tronco Monego – Federal University of Goiás (UFG)

Georges Schnyder - Slow Food Brazil

Greice Bordignon – Social Service of Industry (SESI)

Isa Maria de Gouveia Jorge – Federal Board of Nutritionist (CFN)

Janine Giuberti Coutinho – Ministry of Social Development (MDS, Brazil)

Karla Lisboa Ramos – Pan-American Health Organization (PAHO, Brazil)

Lorena Gonçalves Chaves – Fund for Educational Development (FNDE, Brazil)

Luciana Azevedo Maldonado – State University of Rio de Janeiro (UERJ)

Luisa Maria Oliveira Pinto – State Secretariat of Health of Ceará (SES/CE)

Maísa Beltrame Pedroso – Municipal Secretariat of Health of Rio Grande do Sul (SES/RS)

Márcia Samia Pinheiro Fidelix – Brazilian Nutrition Association (ASBRAN)

Maria Janaína Cavalcante Nunes – Municipal Secretariat of Health of Goiás (SES/GO)

Patrícia Azevedo Feitosa – Municipal Secretariat of Health of Acre (SES/AC)

Pedro Cruz - Federal University of Paraíba (UFPB)

Regicely Aline Brandão Ferreira – Municipal Secretariat of Health of Mauá (SMS MAUA)

Regina Barros Goulart Nogueira - Brazilian National Council on Food and Nutrition Security (CONSEA)

Regina Maria Ferreira Lang - Federal University of Paraná (UFPR)

Renata de Araújo Ferreira – Brazilian Health Surveillance Agency (ANVISA)

Silvia do Amaral Rigon – Brazilian National Council on Food and Nutrition Security (CONSEA)

PARTICIPANTS OF THE WORKING GROUP FOR CONSIDERATION OF SUGGESTIONS FROM THE PUBLIC CONSULTATION

Bruna Pitasi Arguelhes – Ministry of Health (Brazil)

Fernanda Rauber – Ministry of Health (Brazil)

Gisele Ane Bortolini – Ministry of Health (Brazil)

Kelly Poliany de Souza Alves – Ministry of Health (Brazil)

Kimielle Cristina Silva – Ministry of Health (Brazil)

Lorena Toledo de Araújo Melo – Ministry of Health (Brazil)

Mara Lucia dos Santos Costa – Ministry of Health (Brazil)

Renata Guimarães Mendonça de Santana – Ministry of Health (Brazil)

Sara Araújo da Silva – Ministry of Health (Brazil)

ISBN 978-85-334-2242-1
9 788533 422421

DISQUE SAÚDE
136
Ouvidoria Geral do SUS
www.saude.gov.br

Virtual Health Library of the Ministry of Health
www.saude.gov.br/bvs

Organização Pan-Americana da Saúde
Organização Mundial da Saúde
Escritório Regional para as Américas
USP
PNAN
SAÚDE MAIS PERTO DE VOCÊ
SUS
Ministry of Health
BRAZILIAN GOVERNMENT
BRASIL
